# Ion‑Mediated Structural Engineering of Hydrogel Interfaces for Tunable Mechanical and Analyte Diffusion Properties in Electrochemical Biosensors

**DOI:** 10.1002/adma.202515767

**Published:** 2026-03-05

**Authors:** Dongwook Lee, Soo A Kim, Beom‐Jun Shim, Yurim Lee, Tae Young Kim, Sunghyun Park, Yeontaek Lee, Hyeong Gyu Choi, Kayoung Son, Su Bin Han, Keun‐Young Yook, Seo Jung Kim, Won‐Yong Lee, Jungmok Seo, Jayoung Kim

**Affiliations:** ^1^ Department of Chemistry Yonsei University Seoul Republic of Korea; ^2^ Center for Nanomedicine Institute for Basic Science (IBS) Seoul Republic of Korea; ^3^ School of Electrical and Electronic Engineering Yonsei University Seoul Republic of Korea; ^4^ Department of Medical Engineering Yonsei University College of Medicine Seoul Republic of Korea; ^5^ Graduate School of Medical Science, Brain Korea 21 Project Yonsei University College of Medicine Seoul Republic of Korea; ^6^ Department of Battery Engineering Yonsei University Seoul Republic of Korea; ^7^ Department of Pediatrics, Severance Children's Hospital Yonsei University College of Medicine Seoul Republic of Korea; ^8^ Department of Materials Science and Engineering Yonsei University Seoul Republic of Korea

**Keywords:** electrochemical biosensors, hydrogels, implantable biosensors, ionic‐crosslinking, wearable biosensors

## Abstract

Advanced hydrogel interfaces exhibiting finely tuned mechanical characteristics and porosity are essential in wearable and implantable biosensors, mitigating tissue–device mismatches and controlling target analyte transport in biofluids. This work presents an ion‐mediated structural engineering approach designed to meticulously regulate the porous architecture and mechanical robustness of poly(vinyl alcohol)–alginate hydrogels (PAH) through straightforward ionic modulation, effectively addressing inherent trade‐offs between mechanical strength and analyte diffusion. Utilizing three complementary ionic mechanisms—salting‐out, calcium ion chelation, and sequence‐directed biomineralization—hydrogels with tailored porous microstructures are fabricated. The resulting hydrogels exhibit pore sizes ranging from 65 nm to 2.5 µm, mechanical moduli of 50–140 kPa, and controlled analyte diffusion behaviors. Leveraging this structural tunability, two exemplary glucose biosensors are demonstrated: a highly porous hydrogel‐integrated wearable biosensor designed for rapid and sensitive glucose monitoring in sweat, and a densely structured hydrogel‐integrated implantable biosensor optimized for robust and continuous glucose tracking in interstitial fluid. This innovative methodology elucidates critical interconnections between the hydrogel's ion‐mediated microstructural architecture, its mechanical robustness and tunable diffusion characteristics, and the resulting biosensing performance optimized for wearable and implantable applications, thereby advancing the design paradigm for next‐generation personalized biosensor interfaces.

## Graphical abstract

An ion‐mediated structural engineering strategy enables versatile modulation of PVA–Alginate hydrogel microstructure. We systematically examine how different ion combinations affect hydrogel mechanical properties and analyte diffusivity. Applying this novel strategy to hydrogel as the outer membrane in an electrochemical glucose biosensor allows precise tailoring of the linear dynamic range to match desired analyte concentration ranges.

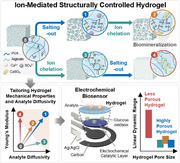

## 1 Introduction

The rapid evolution toward personalized precision medicine and preventive healthcare demands advanced technologies capable of real‐time and continuous monitoring of biological signals [1, 2, 3, 4]. Electrochemical biosensors, which convert biological analytes into quantitative electrical signals, have emerged as essential tools owing to their remarkable sensitivity, rapid response times, facile miniaturization, and seamless integration capabilities [5, 6, 7, 8]. These distinctive advantages highlight their significant potential in sophisticated biomedical applications and next‐generation health monitoring systems. However, the practical implementation of wearable and implantable biosensors is significantly hindered by inherent mechanical and biochemical mismatches at the interfaces between rigid, dry electronic components and soft, hydrated biological tissues [9, 10]. Such mismatches frequently cause increased interfacial impedance, instability in signal acquisition, and reduced reliability of electrochemical biosensors. Consequently, engineering robust bio‐interfaces is crucial, as the quality directly governs the biosensor's long‐term performance, signal fidelity, and functional reliability.

Hydrogels have recently garnered considerable attention as promising interface materials due to their high water content, tissue‐mimicking mechanical flexibility, and excellent biocompatibility [11, 12]. Such compatibility reduces local strain concentration, mitigates foreign‐body responses, such as inflammation and fibrosis, and supports long‐term integration at the tissue interface [13, 14]. In addition, the intrinsic porous structure of hydrogels facilitates biomolecular transport and signal transduction, thereby enhancing sensitivity and accuracy even in complex biological matrices [15, 16]. With pore sizes spanning from nanometers to micrometers, hydrogels create extensive molecular transport pathways, allowing controlled modulation of analyte diffusion [17, 18, 19]. By varying synthesis parameters—cross‐linking density, polymer concentration, and reaction time—their microstructural features, including pore size, morphology, and distribution, can be finely tuned to suit specific biosensing applications. This tunability is crucial for tailoring analyte diffusion and optimizing mechanical properties, thereby improving biosensor stability, reliability, and performance in personalized healthcare. In electrochemical biosensors, analytes diffuse to the electrode surface, where they undergo electrochemical reactions, generating measurable electrical signals [20]. When hydrogels are used as interfaces in electrochemical wearable and implantable biosensors, they must satisfy both mechanical robustness and molecular diffusion characteristics suited to the specific biosensing application. In enzymatic electrochemical biosensors, substrate diffusion through the hydrogel strongly influences sensitivity and the linear dynamic range, underscoring the need for controlled structural tuning [21, 22, 23].

Despite these advantages, traditional hydrogel preparation methods, such as chemical crosslinking or sacrificial templating methods, often face a trade‐off between mechanical strength and molecular diffusion [24]. Enhancing mechanical robustness necessitates denser polymer networks, which in turn limit molecular diffusion. Conversely, introducing porous structures to facilitate rapid analyte diffusion often compromises mechanical robustness. This trade‐off poses a critical challenge in designing biosensors suited to diverse biological environments. For example, wearable biosensors require rapid analyte diffusion and sufficient mechanical resilience to measure low‐concentration biomarkers in noninvasive biofluids under dynamic mechanical conditions—such as sweat glucose monitoring during exercise—where mechanical deformation (stretching, bending) and fluctuating analyte levels occur simultaneously [25, 26, 27]. On the other hand, implantable biosensors require controlled diffusion to prevent biosensor saturation in high‐concentration biofluids, coupled with exceptional mechanical strength to withstand insertion‐induced stresses [28, 29]. They must also incorporate antifouling properties to minimize biofouling‐induced signal deterioration and ensure long‐term stability [30]. In this context, it is essential to achieve a multifunctional interface that possesses optimal porosity to regulate analyte transport while simultaneously providing mechanical robustness and antifouling performance. According to prior studies, hydrogels developed using traditional structural tuning approaches have frequently failed to simultaneously meet these diverse and often competing requirements. This limitation highlights the need for alternative design strategies.

**FIGURE 1:**
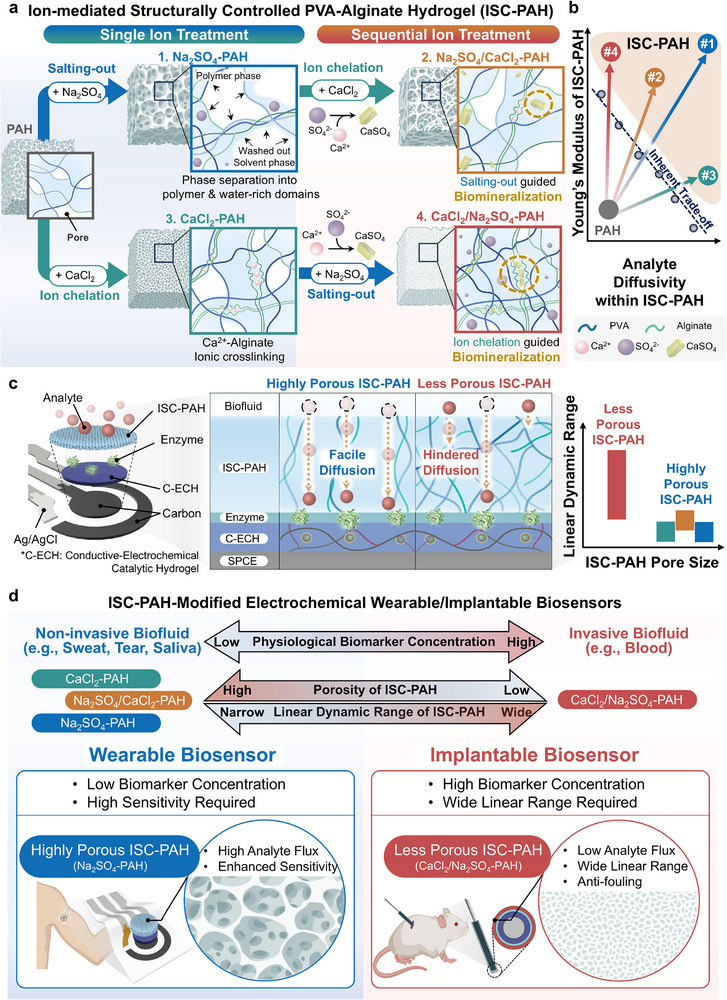
ISC–PAH enables decoupled and concurrent fine‐tuning of mechanical robustness and analyte diffusivity for wearable and implantable electrochemical enzymatic biosensors. (a) Schematic of PAH structural changes and polymer–ion interactions induced by ionic treatments: single‐ion treatments – Na_2_SO_4_–PAH (salting‐out) and CaCl_2_–PAH (ion chelation); sequential‐ion treatments – Na_2_SO_4_/CaCl_2_–PAH (salting‐out followed by ion chelation), CaCl_2_/Na_2_SO_4_–PAH (ion chelation followed by salting‐out). (b) Comparative plot showing the inherent trade‐off between analyte diffusivity and mechanical modulus, as well as the decoupled fine‐tuning enabled by ISC–PAH. (c) Exploded schematic of an enzymatic biosensor in which ISC–PAH serves as the outer diffusion‐control layer, showing structure‐dependent biomarker transport to the electrode interface and modulation of the linear dynamic range. (d) Ion treatment‐mediated fine‐tuning of ISC–PAH microstructure to tune the linear dynamic range to match physiological biomarker levels. Requirements for biomarker monitoring in noninvasive and invasive biofluids using wearable and implantable biosensors, along with configurations and features of a Na_2_SO_4_‐treated PAH‐modified wearable patch for biomarker monitoring in noninvasive biofluid, and a CaCl_2_/Na_2_SO_4_‐treated PAH‐modified implantable probe for biomarker monitoring in invasive biofluid.

In this study, we present an ion‐mediated structurally controlled poly(vinyl alcohol)–alginate hydrogel (ISC–PAH) that enables on‐demand tuning of pore architecture while reinforcing mechanical strength via control of ion species and treatment order in a single hydrogel composition (Figure 1a). By manipulating the identity and sequence of ionic treatments, ISC–PAH decouples properties that are typically coupled in conventional hydrogels, such as mechanical robustness and porosity‐driven analyte diffusivity, thus allowing independent and bidirectional tuning of both parameters (Figure 1b). The structural modulation of ISC–PAH relies on three complementary ion–polymer interactions: (i) *salting‐out* with biocompatible sulfate ions to induce phase separation of PVA chains and promote a hierarchical porous structure that can facilitate analyte transport while polymer crystallization preserves mechanical strength, (ii) *ion chelation* via Ca^2^^+^‐alginate coordination that reinforces the hydrogel network, and (iii) *biomineralization* through controlled CaSO_4_ crystal growth that can lock the initial structure, enabling sequence‐mediated structural modulation. The developed ISC–PAH achieved tunable diffusion properties solely through ionic modulation, without requiring any additional chemical modification or templating process, thereby providing direct control over analyte flux and diffusional behavior (Figure 1c). Owing to this structural tunability, ISC–PAH functions as a universal diffusion‐control interface adaptable to various enzyme‐based electrochemical biosensing environments. For wearable electrochemical biosensors, the highly porous ISC–PAH facilitates analyte transport and enables high‐sensitivity detection of low‐abundance biomarkers in sweat. In contrast, for implantable electrochemical biosensors, the low‐porosity ISC–PAH forms a dense and reinforced diffusion‐control layer that precisely limits analyte flux, resists fouling, and withstands insertion, enabling stable and long‐term monitoring within interstitial‐fluid environments (Figure 1d). Across configurations determined by ion identity and treatment sequence, tissue‐compatible mechanical properties are maintained, allowing this single hydrogel platform to be predictably tailored to meet the distinct requirements of wearable and implantable electrochemical sensing without additional chemistries or separate materials. Collectively, these findings demonstrate that ion‐sequence‐controlled ISC–PAH can be rationally engineered to satisfy diverse biosensing demands. Therefore, ISC–PAH represents a single‐material, tunable interface platform and a universal, scalable strategy for next‐generation electrochemical biosensing applications.

## 2 Result and Discussion

### 2.1 Ion‐Mediated Structural Engineering of Hydrogels

The ion‐mediated strategy was applied to systematically tailor the porous microstructure of freeze–thaw‐prepared pristine PAH composites by immersing them in selected salt solutions, enabling simultaneous control of analyte diffusion and structural stability without the need for separate chemical modifications. Among the wide range of multivalent cations and kosmotropic anions reported to influence hydrogel formation through ionic crosslinking or structural modulation, many of these exhibit cytotoxicity. For example, Sr^2^^+^ and Ba^2^^+^ raise concerns due to their potential toxicity and regulatory concerns, while Zn^2^^+^, Cu^2^^+^, and Fe^3^^+^ are associated with oxidative stress or coloration effects that limit their biomedical applicability [31]. In contrast, Ca^2^^+^ is the most established ionic crosslinker for alginate, providing stable and reproducible “egg‐box” junctions with proven biomedical safety [32, 33]. Moreover, C_6_H_5_O_7_^3−^ and SO_4_^2−^ are known to exhibit the strongest salting‐out effects. However, while C_6_H_5_O_7_^3−^ shows poor biocompatibility, SO_4_^2−^ is a biocompatible kosmotropic anion that effectively promotes polymer–polymer interactions and porosity through its salting‐out effect [34]. Based on these considerations, SO_4_^2−^ and Ca^2^^+^ were deliberately selected as the primary ions for this study. Three distinct ion‐mediated mechanisms were subsequently designed using these ions to modulate the hydrogel microstructure.

(1) *Salting‐out* was implemented by immersing pristine PAH in a Na_2_SO_4_ solution. According to the Hofmeister series, both citrate and sulfate ions are highly effective kosmotropes for inducing a salting‐out effect [35, 36]. High concentrations of kosmotropic SO_4_^2^^−^ ions preferentially interact with bound water molecules in the polymer hydration layer, competitively displacing them into the bulk phase and thereby strengthening hydrogen‐bonded interactions between adjacent polymer chains. This process ultimately drives the polymer–water system into the spinodal decomposition regime, causing spontaneous phase separation into polymer‐rich and solvent‐rich domains. Upon salt removal, the solvent‐rich phase transitions into an interconnected porous network, forming hierarchical porous structures that enhance analyte diffusion [36]. Although citrate ions are known to exhibit one of the strongest kosmotropic effects in the Hofmeister series, their cytotoxicity and propensity to form undesired chelation complexes with alginate led us to select SO_4_^2^^−^ for its superior biocompatibility and inclusion of only monovalent counter‐cations (Na^+^) to prevent unintended metal–alginate coordination.

(2) *Ion chelation* was achieved by immersing PAH in a CaCl_2_ solution, employing the well‐established egg‐box coordination mechanism [37, 38]. Divalent Ca^2^^+^ ions rapidly diffuse into the hydrogel matrix, selectively binding to carboxylate groups (–COO^−^) of guluronic acid (G‐block) segments within alginate chains to form discrete ionic crosslinking nodes, significantly enhancing structural stability. This coordination mechanism is based on interactions between multivalent cations, such as Fe^3^^+^, Zn^2^^+^, and specifically Ca^2^^+^ ions, and guluronic acid segments in alginate. While this crosslinking improves network integrity, the chaotropic nature of Ca^2^^+^ can also induce a local salting‐in effect, thickening the PVA hydration shell and potentially reducing the overall crystallinity of the PVA phase.

(3) *Biomineralization* was implemented via sequential ionic treatments with Ca^2^^+^ and SO_4_^2^^−^ ions, leading to in situ CaSO_4_ crystal formation, thereby forming hybrid organic–inorganic anchoring points within the hydrogel. In the CaCl_2_ → Na_2_SO_4_ sequence, pre‐established Ca^2^^+^–alginate ionic nodes act as high‐charge‐density nucleation sites, lowering the free energy barrier for CaSO_4_ crystallization [39, 40]. The resulting microcrystals occupy interstitial spaces between polymer chains, and Ca^2^^+^ ions at the crystal surface form additional coordination interactions with alginate –COO^−^ groups, creating a denser and mechanically reinforced network (ion chelation‐guided biomineralization). In contrast, the Na_2_SO_4_ → CaCl_2_ sequence first induces salting‐out‐driven pore formation prior to crystal growth, yielding a more open microarchitecture (salting‐out‐guided biomineralization).

In summary, the identity and sequence of ionic treatments act as key determinants of the resulting hydrogel microarchitecture, which is ultimately stabilized by CaSO_4_‐based hybrid organic–inorganic anchoring points formed during sequential ion introduction (Figure S1). Leveraging this versatility, we systematically investigated four distinct ISC–PAH variants: (i) Na_2_SO_4_–PAH, (ii) CaCl_2_–PAH, (iii) CaCl_2_/Na_2_SO_4_–PAH, and (iv) Na_2_SO_4_/CaCl_2_–PAH, to explore how ionic treatment pathways influence hydrogel microstructure and subsequent biosensor performance. This ion–polymer interaction framework establishes a robust and generalizable strategy for tailoring hydrogel architecture in biosensing and other biomedical applications that require concurrent control of mass transport and mechanical integrity.

### 2.2 Characterization of Porosity and Diffusivity in ISC–PAHs

**FIGURE 2:**
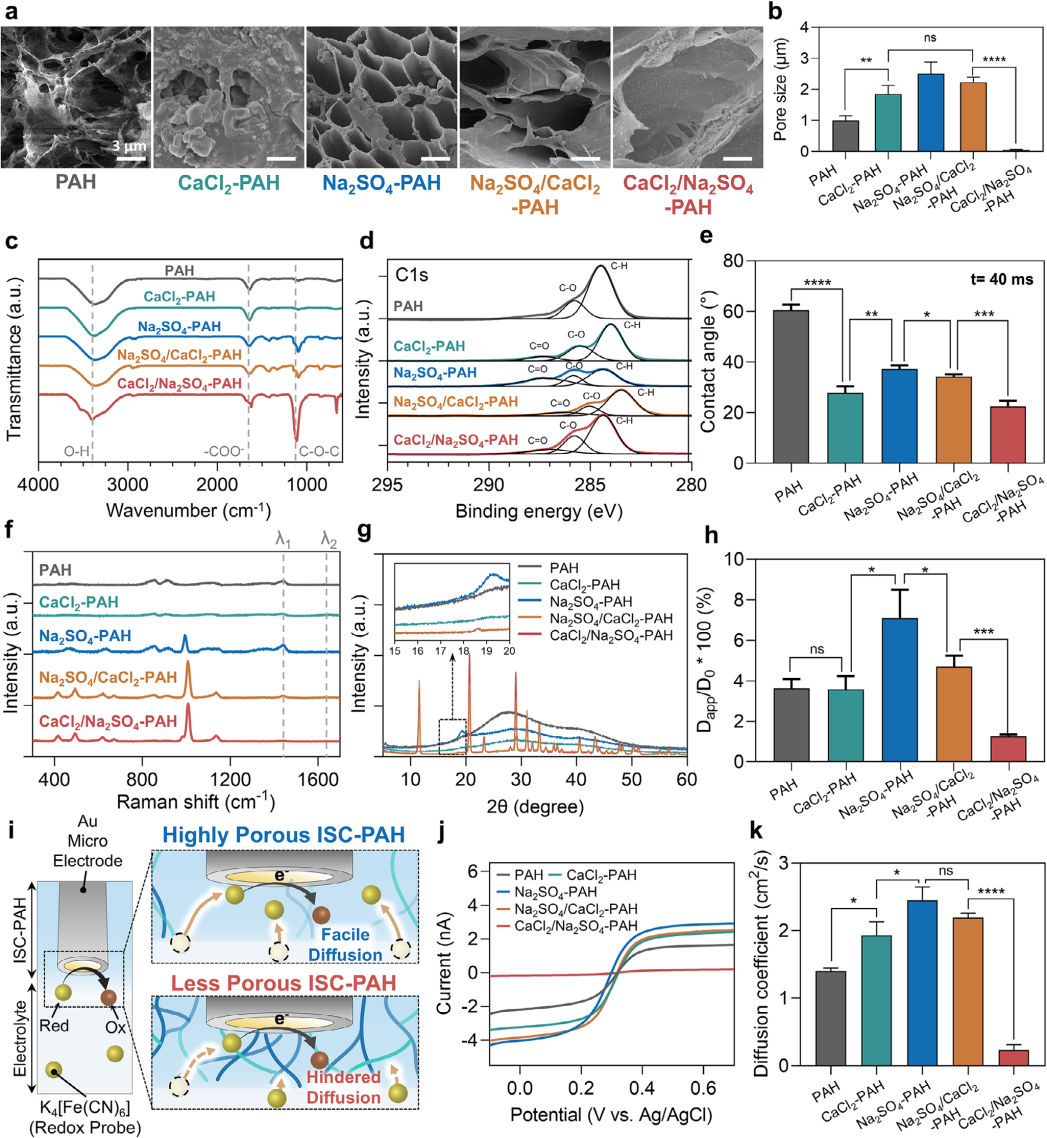
Microstructure and diffusion properties of analytes within pristine PAH and ISC–PAHs. (a) SEM images showing variations in microstructure under different ion treatments. (b) Corresponding average pore sizes determined by porosimetry. Data are presented as mean ± SD, *n* = 3. (c) FTIR spectra, (d) XPS spectra, (e) time‐dependent contact angle images and contact angle of ISC–PAHs at 40 ms. Data are presented as mean ± SD, *n* = 3. (f) Raman spectra of pristine PAH and ISC–PAHs. (g) XRD patterns. (h) Relative diffusion coefficients (*D*_app_/*D*_0_*100) of 4 kDa FITC–dextran in pristine PAH and ISC–PAHs, calculated using the Soumpasis model. *D*_app_ denotes the apparent diffusion coefficient, and *D*_0_ represents the free diffusion coefficient in solution. (i) Schematic illustration depicting the evaluation of the diffusion behavior of a redox probe across pristine PAH and ISC–PAHs using electrochemical measurements. (j) LSV curves obtained using Au microelectrodes (diameter: 12.5 µm) embedded within hydrogel cubes in a 5 mM K_3_Fe(CN)_6_/K_4_Fe(CN)_6_ solution, and (k) apparent diffusion coefficients were calculated from the steady‐state limiting currents. Data are presented as mean ± SD, *n* = 3. (*p < 0.05, **p < 0.01, ***p < 0.001, ****p < 0.0001). ns, not significant.

The pore architecture of pristine PAH and ISC–PAHs was characterized by scanning electron microscopy (SEM) and porosimetry, revealing distinct morphologies and pore size distributions under various ionic treatment conditions (Figure 2a,b). The pristine PAH displayed a compact network with an average pore size of 1.08 µm. When treated with Na_2_SO_4_, a pronounced salting‐out effect generated a highly porous morphology, increasing the average pore size to approximately 2.50 µm. Separately, Ca^2^^+^ treatment created uniform ionic crosslinks with alginate within the hydrogel network, yielding an average pore size of 1.85 µm—approximately 71.3% larger than that of the pristine PAH.

To create a diffusion‐hindered architecture, Ca^2^^+^ treatment was followed by SO_4_^2^^−^ treatment in a sequential process. In this condition, Ca^2^^+^ formed a uniform ion‐chelation network with alginate, producing evenly distributed nucleation sites for subsequent biomineralization. Subsequent SO_4_^2^^−^ exposure promoted controlled CaSO_4_ crystal growth from these sites, resulting in a densely packed structure with an average pore size of 65 nm—a reduction of approximately 94% compared to pristine PAH. Rod‐shaped CaSO_4_ crystals were observed by SEM, consistent with successful in situ biomineralization. In the reverse sequence (SO_4_^2^^−^→Ca^2^^+^; Na_2_SO_4_/CaCl_2_–PAH), the hydrogel retained a porous network (≈2.23 µm), with CaSO_4_ forming primarily as thin surface coatings rather than within the internal network. This outcome reflects the salting‐out effect establishing porosity first, followed by surface‐localized crystal growth upon Ca^2^^+^ exposure. These observations confirm that the order of ionic treatments strongly influences nucleation, crystal distribution, and the resulting microstructure in biomineralized hydrogels.

To verify this difference stem from distinct crystallization behavior of CaSO_4,_ we performed time‐resolved SEM to compare the spatiotemporal dynamics of CaSO_4_ formation between CaCl_2_/Na_2_SO_4_–PAH and Na_2_SO_4_/CaCl_2_–PAH, highlighting the critical influence of ion treatment sequence on nucleation and crystal growth (Figures S2 and S3). In the Ca^2+^ → SO_4_^2−^ condition, hydrogels were first treated with Ca^2+^. Upon subsequent SO_4_^2^^−^ exposure, time‐resolved SEM showed gradual and homogeneous CaSO_4_ crystallization. Initially (0–30 min), the surface remained smooth with sparse nuclei, whereas between 1 and 12 h, microcrystals progressively grew and interconnected, forming a stabilized microcrystalline network by 24 h. This diffusion‐controlled crystallization is driven by Donnan‐regulated Ca^2^^+^ distribution and uniform supersaturation throughout the gel, leading to simultaneous nucleation and uniform growth [39, 40]. Rather than random ion mixing, this behavior arises from ion–polymer interactions that spatially localize Ca^2^^+^, enhancing crystallization control. In contrast, SO_4_^2−^ → Ca^2+^ showed a markedly different crystallization behavior. Salting‐out with SO_4_^2−^ first created a porous, dehydrated network. Upon Ca^2^^+^ exposure, SEM revealed rapid formation of large, faceted CaSO_4_ crystals in localized regions (30 min–1h), indicating burst nucleation. These crystals continued to grow but remained heterogeneously distributed. Even after 24 h, unmineralized domains coexisted with oversized aggregates. This spatial heterogeneity results from localized supersaturation spikes at reaction–diffusion fronts, where SO_4_^2^^−^ retained within pores rapidly reacts with diffusing Ca^2^^+^. This localized reaction generates transient supersaturation, inducing uneven nucleation and local Ca^2^^+^ depletion, thereby producing heterogeneous crystal growth. These findings highlight the critical role of ion sequence in controlling mineralization dynamics.

Following the SEM and porosimetry analyses of ion‐dependent porous structure variations in ISC–PAHs, Fourier‐transform infrared spectroscopy (FT‐IR) was employed to investigate molecular‐level interchain interactions underlying these structural changes (Figure 2c). Across all samples, the spectra displayed the broad O*─*H stretching band (∼3290 cm^−1^), the alginate carboxylate asymmetric stretch (∼1590‒1610 cm^−1^), and the C*─*O*─*C stretching band (1000–1200 cm^−^^1^) [41, 42]. The C*─*O*─*C region, corresponding to the semicrystalline domains of PVA within the PAH network, exhibited the most pronounced dependence on the ionic treatment. To quantify these variations, the integrated C*─*O*─*C band area was normalized to the COO*^─^* stretching peak area (1600–1650 cm^−1^), displaying area ratios (*R* = A_C_ *_─_*_O_ *_─_*_C_/A_COO_^−^). Comparison of the C*─*O*─*C band area ratios revealed distinct differences across the ion‐treated samples. CaCl_2_–PAH exhibited an area ratio comparable to that of the pristine PAH, indicating minimal structural reorganization. In contrast, both Na_2_SO_4_–PAH and Na_2_SO_4_/CaCl_2_–PAH showed approximately a tenfold increase, while CaCl_2_/Na_2_SO_4_–PAH exhibited the highest ratio—about 26 times greater than that of the pristine PAH (Table S1). These quantitative results demonstrate that the ionic sequence critically dictates polymer crystallization and network densification. Specifically, in Na_2_SO_4_–PAH, the salting‐out effect promoted PVA chain alignment and crystallization, markedly increasing the peak area ratio. In CaCl_2_–PAH, however, the chaotropic nature of Ca^2^^+^ ions disrupted the hydrogen‐bonded water structure, hindering PVA crystallization and leading to only a modest increase [39]. In CaCl_2_/Na_2_SO_4_–PAH, initial Ca^2^^+^ chelation established nucleation sites, and subsequent SO_4_^2^^−^ treatment facilitated uniform CaSO_4_ crystal growth throughout the polymer matrix, strongly restricting chain mobility and yielding the most significant enhancement in structural ordering. On the other hand, in Na_2_SO_4_/CaCl_2_–PAH, once the porous salting‐out architecture was formed by the initial sulfate treatment, the subsequent Ca^2^^+^ step mainly served to preserve the existing structure via CaSO_4_ mineralization, rather than further densifying it.

X‐ray photoelectron spectroscopy (XPS) analysis was conducted to confirm surface‐level ionic incorporation and coordination, supporting the findings in the FT‐IR spectra (Figure 2d). Pristine PAH exhibited dominant nonpolar C*─*C/C*─*H peaks (≈284.8 eV), moderate polar C*─*O peaks (≈286.4 eV), and O*─*C═O peaks (≈288.5 eV). Na_2_SO_4_–PAH showed increased C*─*O intensity and reduced C*─*H peaks, consistent with enhanced crystallinity induced by salting‐out. CaCl_2_–PAH displayed minimal spectral changes, reflecting the influence of chaotropic Ca^2^^+^ ions in disrupting crystallization. The CaCl_2_/Na_2_SO_4_–PAH exhibited increased polar C*─*O (≈ 286.4 eV) and O*─*C═O (≈288.5 eV) peaks with a concurrent decrease in nonpolar C*─*C/C*─*H (≈284.8 eV), indicating a greater exposure of hydrophilic functional groups (*─*OH and *─*COO^−^) on the surface. Ca 2p spectra further substantiated Ca^2+^ incorporation and mineralization, showing a well‐defined Ca 2p doublet at ≈347.2 eV (2p_3/2_) and ≈350.6 eV (2p_1/2_), consistent with CaSO_4_ (Figure S4). Na_2_SO_4_/CaCl_2_–PAH, in comparison, exhibited intermediate polar peak intensities in C1s spectra, suggesting partial reversal of the initial crystallinity enhancement due to surface‐oriented biomineralization. This structural rearrangement was further supported by static water contact angle measurements (Figure S5). The CaCl_2_/Na_2_SO_4_–PAH exhibited the lowest initial contact angle of 22.6° ± 2.1° measured at 40 ms after droplet deposition, which rapidly decreased and nearly approached 0° by 600 ms, indicating extremely fast water spreading and superior surface wettability, compared with 60.4° ± 2.3° for pristine PAH measured at 40 ms (Figure 2e). The combined XPS and contact angle results clearly demonstrate that Ca^2^^+^‐induced polymer chain rearrangement and biomineralization significantly increase surface polarity, thereby facilitating the formation of a stable bound‐water layer on the hydrogel interface. This hydration layer acts as a physical and energetic barrier that prevents direct contact of proteins, such as bovine serum albumin (BSA) with the surface through hydration‐mediated steric and entropic repulsion [43, 44, 45, 46]. In addition, the ionic crosslinking and embedded CaSO_4_ nanocrystals impart mechanical integrity that stabilizes this hydrated interface. Consequently, the CaCl_2_/Na_2_SO_4_–PAH maintains sensor performance over repeated insertion cycles and long‐term operation by effectively suppressing biofouling‐induced signal deterioration. This mechanism aligns with previous reports that hydrophilic polymer networks with strong hydration capacity minimize interfacial free energy and resist nonspecific protein adsorption.

Moreover, X‐ray diffraction (XRD) analysis was performed to characterize ion‐treatment‐induced crystallinity in the ISC‐PAH matrix. The observed peaks clearly showed distinct structural changes in the hydrogel crystal structure (Figure 2f). The results confirmed enhanced PVA crystallinity in Na_2_SO_4_–PAH and reduced crystallinity in chaotropic Ca^2^^+^‐treated samples compared with pristine PAH [47]. In biomineralized samples, sharp diffraction peaks corresponding to CaSO_4_ crystals (e.g., at 2θ = 11.7°, 20.7°, etc.) were observed, confirming the successful formation of a new inorganic crystalline phase. This newly formed phase is expected to significantly improve the mechanical strength and structural stability of the hydrogel network [40, 48].

Building on the above phase and surface analyses, Raman spectroscopy was performed to elucidate the polymer–inorganic interactions formed by CaSO_4_ crystals within the ISC–PAH network (Figure 2g). The separation between the asymmetric and symmetric carboxylate stretching bands Δν (ν_as_ ‐ ν_s_, ν_as_ ≈ 1590–1610 cm^−1^, ν_s_ ≈ 1410–1425 cm^−1^), consistently decreased in the Ca^2+^‐treated groups, indicating a shift toward chelating/bridging Ca^2+^‐carboxylate coordination rather than monodentate binding. Concurrently, biomineralized samples exhibited a pronounced sulfate symmetric stretching band, ν_1_(SO_4_^2^^−^, ≈1008 cm^−^^1^), together with an asymmetric stretching feature (≈1100 cm^−^^1^); both were strongest for the CaCl_2_→Na_2_SO_4_ sequence, supporting uniform CaSO_4_ formation throughout the network. In addition, changes in the relative intensity and linewidth of the PVA backbone band (≈1140–1150 cm^−^^1^) point to increased chain alignment via salting‐out and reduced local chain mobility following mineralization‐induced network densification. In summary, the Raman results corroborate the conclusions from XRD and XPS at the level of molecular vibrations, providing qualitative and quantitative evidence that CaSO_4_–polymer interfacial coupling promotes interfacial hydration, mechanical stability, and, ultimately, enhanced antifouling performance.

To evaluate the functional impact of microstructural changes on intra‐hydrogel molecular diffusion characteristics, fluorescence recovery after photobleaching (FRAP) analysis was conducted using fluorescein isothiocyanate (FITC)–dextran as a model probe. Pristine PAH and various ISC–PAHs were tested with FITC–dextran of three different molecular weights (4, 20, and 40 kDa) to capture size‐dependent diffusion behaviors (Figure S6). These sizes represent a physiologically relevant range from small metabolites to medium‐sized proteins, allowing a comprehensive evaluation of diffusivity depending on molecular size. The results revealed that, except for Na_2_SO_4_–PAH, all samples exhibited restricted diffusion of 40 kDa FITC–dextran, indicating that their microstructures effectively limit the transport of large molecules above a critical size threshold. In contrast, Na_2_SO_4_–PAH showed no apparent diffusion barrier even for 40 kDa probes, reflecting that its open and porous network formed through salting‐out provides a sufficient diffusion pathway even for larger molecules. Conversely, CaCl_2_/Na_2_SO_4_–PAH exhibited hindered permeability even for 4 kDa FITC–dextran, suggesting that its compact, mineralized network provides pronounced resistance to mass transfer.

To quantitatively analyze these diffusion characteristics, the mobile fraction (MF) and half recovery time (τ_1/2_) for each sample were obtained from fluorescence recovery curves (Figure S7). Na_2_SO_4_–PAH showed the highest MF (>98%) at all probe sizes and the shortest τ_1/2_ in most cases. However, for the 40 kDa FITC–dextran, Na_2_SO_4_–PAH did not present the shortest τ_1/2_, likely due to its exceptionally high MF, which allows rapid but more gradual recovery dynamics compared to other samples. This behavior remains consistent with the highly porous and low‐density network structure of Na_2_SO_4_–PAH. Conversely, CaCl_2_/Na_2_SO_4_–PAH displayed a markedly lower MF (≈82.8%) and prolonged τ_1/2_ (≈5.84 s), confirming its strong restriction of small‐molecule diffusion. To enable cross‐comparison across hydrogels for 4 kDa FITC–dextran, the Soumpasis model for 2D circular bleaching geometry was applied to determine the apparent diffusion coefficient (*D*_app_). By normalizing *D*_app_ to the known free‐solution diffusion coefficient (*D*_0_) for 4 kDa FITC–dextran, the relative diffusivity metric (*D*_app_/*D*_0_*100) was calculated. The results revealed a clear structure‐dependent trend: Na_2_SO_4_–PAH exhibited the least hindered diffusion among the ISC–PAHs (≈7.1 ± 1.4%), whereas CaCl_2_/Na_2_SO_4_–PAH showed the greatest diffusional hindrance (≈ 1.26% ± 0.09%) (Figure 2h).

To further validate the diffusion characteristics identified by FRAP, electrochemical linear sweep voltammetry (LSV) was conducted using pristine PAH and four ISC–PAH variants as diffusion‐limiting layers. In these measurements, the redox probe (K_3_[Fe(CN)_6_]/K_4_[Fe(CN)_6_]) was used as the diffusional reporter within the hydrogel (Figure 2i,j and Figure S8). The steady‐state current (*I*_ss_) generated by stable hemispherical diffusion at the microelectrode interface is directly proportional to the diffusion coefficient (D) according to the equation: *I*_ss_ = 4nFDCr [49]. Anodic steady‐state currents from the redox probe were consistently observed for pristine PAH and all ISC–PAHs. Diffusion coefficients, derived from the measured steady‐state currents, followed the order: Na_2_SO_4_–PAH > Na_2_SO_4_/CaCl_2_–PAH > CaCl_2_–PAH > PAH > CaCl_2_/Na_2_SO_4_–PAH (Figure 2k). This sequence corresponded closely to the average pore sizes determined by SEM and porosimetry. These findings demonstrate that the ion‐mediated structural engineering strategy can effectively tailor the hydrogel microarchitecture, allowing controlled regulation of analyte diffusion rates at the interface—an essential attribute for designing tunable electrochemical biosensors.

### 2.3 Mechanical Properties and Biocompatibility of ISC–PAHs

**FIGURE 3:**
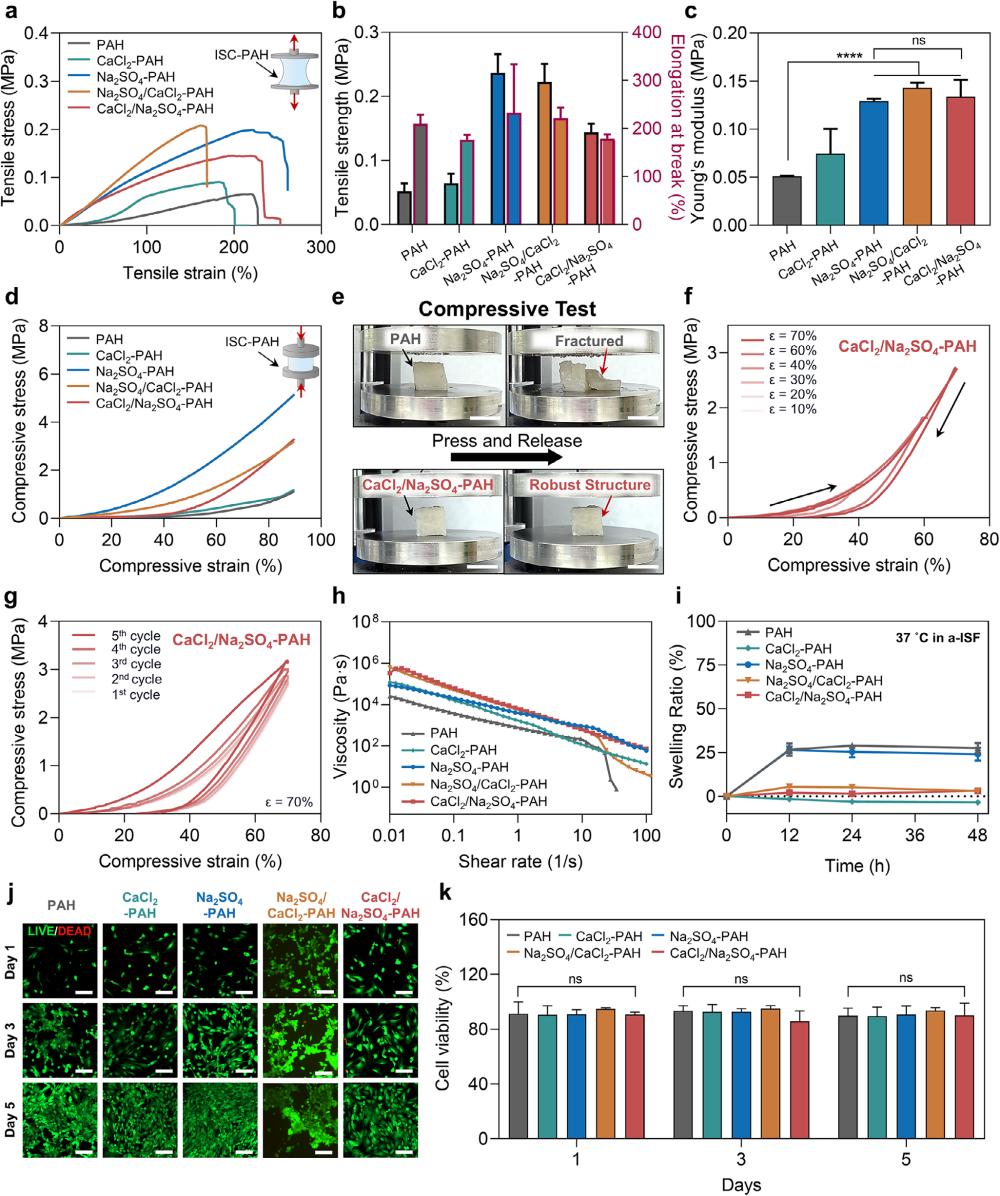
Mechanical properties and biocompatibility of pristine PAH and ISC–PAHs. (a) Tensile stress–strain curves. (b) Quantitative comparison of tensile strength and elongation at break. Data are presented as mean ± SD, *n* = 3. (c) Young's modulus obtained from tensile tests. Data are presented as mean ± SD, *n* = 3. (d) Compressive stress–strain curves obtained from uniaxial compression tests. (e) Photographs of PAH and CaCl_2_/Na_2_SO_4_–PAH under compressive loading (scale bar = 2 cm). (f) Cyclic compressive curves of CaCl_2_/Na_2_SO_4_–PAH at different strain levels (*ε* = 10%–70%). (g) Fatigue resistance over five consecutive compression cycles (*ε* = 70%). (h) Viscosity as a function of shear rate, showing shear thinning behavior. (i) Swelling ratio in artificial interstitial fluid (a‐ISF) over time at 37°C. Data are presented as mean ± SD, *n* = 3. (j) Live/dead fluorescence images of NIH‐3T3 cells encapsulated in the hydrogels at days 1, 3, and 5 (scale bar = 50 µm). (k) Quantitative cell viability (%) over 5 days, indicating no significant cytotoxicity. Data are presented as mean ± SD, *n* = 3. (*p < 0.05, **p < 0.01, ***p < 0.001, ****p < 0.0001); ns, not significant.

The mechanical properties of ISC–PAHs were enhanced alongside their diffusion characteristics through structural modulation via selective ion treatments, as evidenced by tensile, compressive, and rheological measurements. To assess strength and stretchability under uniaxial strain, tensile tests were conducted. All ISC–PAHs exhibited enhanced mechanical performance compared to the pristine PAH (Figure 3a and Figure S9). Notably, the Na_2_SO_4_–PAH, featuring a hierarchical porous structure generated by salting‐out, exhibited the highest tensile strength (≈237 kPa) and elongation at break (≈347%), indicating concurrent increases in strength and extensibility. This simultaneous enhancement in strength and extensibility can be attributed to the ion‐specific salting‐out mechanism that generates hierarchical porosity and microcrystalline domains within the PVA network.

Strongly kosmotropic SO_4_^2^^−^ ions withdraw hydration water from PVA chains, lowering solvent quality and triggering phase separation into polymer‐rich and water‐rich domains. In the polymer‐rich regions, dehydrated PVA chains align and crystallize into nanodomains that act as physical crosslinking points. The water‐rich domains are subsequently removed during rinsing, forming interconnected 1–10 µm pores that constitute a hierarchical network. These structural motifs collectively enhance mechanical robustness: the crystalline domains restrict chain slippage and act as reinforcing nodes, whereas the porous architecture redistributes stress and promotes crack deflection. Amorphous tie‐chains bridging crystallites further absorb strain, enabling large deformation before failure. This ion‐driven structuring accounts for the concurrent improvements in tensile strength and elongation observed for Na_2_SO_4_–PAH, where the hierarchical porous network and crystalline domains act synergistically to enhance both robustness and flexibility.

Moreover, the sequence of ionic treatments critically governs the resulting microstructure and mechanical behavior of the ISC–PAHs. In the CaCl_2_→ Na_2_SO_4_ sequence, Ca^2^^+^‐alginate coordination occurs first, establishing uniformly distributed ionic crosslinking nodes that guide CaSO_4_ nucleation within confined polymer domains. The resulting dense crystal deposition enhances stiffness and compressive strength but limits extensibility. Conversely, in the Na_2_SO_4_→CaCl_2_ sequence, salting‐out‐driven pore formation precedes biomineralization, creating a more open, hierarchical network in which CaSO_4_ preferentially deposits along pore walls, preserving chain mobility and improving deformability. This sequence‐dependent biomineralization mechanism highlights how programmed ion order enables precise tuning of rigidity and flexibility within a single hydrogel platform.

Both Na_2_SO_4_/CaCl_2_–PAH and CaCl_2_/Na_2_SO_4_–PAH also exhibited robust mechanical properties with tensile strengths of ≈222 and ≈144 kPa and elongations at break of ≈243% and 188%, respectively (Figure 3b), reflecting the reinforcing effect of biomineralization. Young's modulus values further supported these findings (Figure 3c): Na_2_SO_4_–PAH, Na_2_SO_4_/CaCl_2_–PAH, and CaCl_2_/Na_2_SO_4_–PAH measured 135 ± 9, 143 ± 5, and 134 ± 15 kPa, respectively, whereas pristine PAH and CaCl_2_–PAH were more compliant at 54 ± 6 and 67 ± 31 kPa. These moduli fall within the range typical of human soft tissues (typically 10–200 kPa) [11], indicating that ion‐mediated structural control strategy can produce mechanically compatible hydrogel interfaces. In addition, we compared the results of the present study with previously reported ion‐treated or structurally modified PAH systems (Table S2). This improvement in mechanical performance corresponds to the enhanced diffusivity profiles of ISC–PAHs, indicating that ion‐regulated structural modulation enables a partial decoupling between mechanical reinforcement and analyte transport—an effect that is uncommon in conventional porous hydrogels. The ISC–PAHs developed in this work exhibited a balanced enhancement in both toughness and extensibility, demonstrating simultaneous improvement of these typically competing properties. This unique behavior is attributed to the interplay between polymer chain mobility and crosslinking density, which generally governs the intrinsic trade‐off between mechanical strength and deformability in conventional hydrogels. Specifically, kosmotropic SO_4_^2^^−^ ions induce salting‐out‐driven crystallization of PVA chains and the formation of hierarchical porous and crystalline domains, which dissipate stress and allow large deformation. At the same time, Ca^2^^+^ ions establish reversible ionic crosslinking with alginate and promote in situ CaSO_4_ biomineralization, generating load‐bearing junctions that reinforce the network and prevent premature fracture. Consequently, this ion‐mediated structural modulation yields hydrogels with high toughness and extensibility while preserving a physiologically relevant Young's modulus.

Given that soft tissue interfaces in biological environments are subjected to complex mechanical stimuli, including compression and shear, compressive performance was also evaluated (Figure 3d). Under 90% compressive strain, Na_2_SO_4_–PAH, CaCl_2_/Na_2_SO_4_–PAH, and Na_2_SO_4_/CaCl_2_–PAH exhibited remarkably high compressive strengths of ≈5.17, 3.32, and 3.21 MPa, respectively, all exceeding that of pristine PAH (<1 MPa). Upon compression and release, pristine PAH and CaCl_2_–PAH fractured and failed to recover their original shape, while Na_2_SO_4_–PAH, CaCl_2_/Na_2_SO_4_–PAH, and Na_2_SO_4_/CaCl_2_–PAH maintained macroscopic integrity and elasticity (Figure 3e and Figure S10). This structural stability under large compressive deformation reflects high mechanical robustness, attributed to the combined effects of ionic coordination and salt‐induced physical crosslinking that create an energy‐dissipative yet resilient network architecture. While ion‐toughened hydrogels have been reported, continuous and bidirectional tuning of both porosity and reinforcement within a single formulation—achieved by programming ion identity and sequence—has not, to our knowledge, been demonstrated. This is accomplished here without additional chemistries or templates. The compressive stress–strain response of CaCl_2_/Na_2_SO_4_–PAH across strain levels from 10% to 70% exhibited nonlinear stiffening behavior, a hallmark of soft biological tissues, with no evidence of mechanical failure even at high strain (Figure 3f). Cyclic compression tests also revealed that CaCl_2_/Na_2_SO_4_–PAH maintained consistent stress–strain profiles across five consecutive cycles at 70% strain, with minimal hysteresis and no structural degradation (Figure 3g). In contrast, pristine PAH and CaCl_2_–PAH showed significantly lower stress responses and a tendency toward permanent deformation (Figure S11). Overall, these results confirm the excellent fatigue resistance and elastic recoverability, both of which are essential attributes for materials interfacing with dynamic biological tissues.

Rheological measurements further validated the flow behavior and viscoelasticity of ISC–PAH arising from the ion‐treatment process. All samples displayed shear‐thinning behavior, characteristic of viscoelastic networks (Figure 3h). Among them, CaCl_2_/Na_2_SO_4_–PAH displayed the highest zero‐shear viscosity, resulting from strong chain mobility restriction provided by the in situ biomineralized CaSO_4_ crystals serving as multi‐fixation points. Subsequent small‐amplitude oscillatory shear (SAOS) frequency sweeps (Figure S12) revealed that all Ca^2+^‐treated hydrogels increased the storage modulus (*G*′) relative to pristine PAH, consistent with the formation of ionic coordination points and reduced chain mobility. The CaCl_2_→Na_2_SO_4_ sequence exhibited the largest *G*′, whereas the Na_2_SO_4_→CaCl_2_ sequence displayed the lowest loss factor (tan δ, defined as *G*″/*G*′ at 1 Hz), indicating an elastic, densely crosslinked network. Notably, CaCl_2_→Na_2_SO_4_ also produced the highest tan δ, reflecting high stiffness combined with pronounced energy dissipation. These rheological characteristics suggest that the CaCl_2_→Na_2_SO_4_‐treated hydrogel forms a highly reinforced yet viscoelastic network, in which strong Ca^2^^+^‐carboxylate coordination and in situ CaSO_4_ crystallization limit chain mobility while enabling controlled energy dissipation. The elevated tan δ observed for this sequence likely originates from reversible Ca^2^^+^‐alginate bond exchange within the dense network and interfacial friction between polymer chains and newly formed CaSO_4_ microdomains, leading to localized stress relaxation rather than structural instability. Taken together, these findings indicate that selective ionic treatments enable targeted structural tailoring of ISC–PAHs, allowing modulation of mechanical strength and elasticity, and providing a promising strategy for designing robust and mechanically adaptive hydrogel interfaces. To further correlate the measured viscoelastic parameters with the molecular‐scale network architecture, the effective mesh size (*ε*) of each hydrogel was estimated from the storage modulus (*G*′) using the rubber elasticity theory (RET) (see Methods Section for details). The calculated *ε* exhibited a strong inverse correlation with *G*′ (Table S3), with values of approximately 16.5 nm for pristine PAH, 7.85 nm for Na_2_SO_4_–PAH, and 2.66 nm for CaCl_2_/Na_2_SO_4_‑PAH, in good agreement with SEM‑derived pore dimensions and FRAP‑determined diffusion coefficients, except for Na_2_SO_4_–PAH, which showed a smaller *ε* than expected due to its increased network stiffness arising from nanocrystalline reinforcement despite its high porosity. Accordingly, these results establish that the RET‑derived mesh size serves as a quantitative descriptor linking mechanical stiffness and molecular transport, bridging the structural hierarchy between network elasticity and diffusional dynamics.

In addition, to assess long‐term dimensional stability in physiologically relevant environments, the swelling behavior of the hydrogels was evaluated in DI water as a control, artificial sweat (a‐sweat), and artificial interstitial fluid (a‐ISF) at temperatures of 25°C and 37°C (Figure 3i and Figure S13). Overall, swelling behavior was strongly dependent on the ionic strength of the surrounding media, yet showed consistent trends across all conditions. CaCl_2_–PAH exhibited significantly reduced swelling or slight deswelling under all conditions; Na_2_SO_4_–PAH, in contrast, showed increased swelling. Sequentially ion‐treated hydrogels (Na_2_SO_4_/CaCl_2_–PAH and CaCl_2_/Na_2_SO_4_–PAH) displayed effectively suppressed swelling. For instance, Na_2_SO_4_–PAH showed a swelling ratio of approximately 24%, which is attributed to osmotic influx driven by residual unbound sulfate ions in a‐ISF at 37°C (Figure 3i and Figure S14a). In contrast, CaCl_2_–PAH exhibited slight deswelling due to reverse osmotic pressure and network densification through ion‐chelation binding (Figure S14b). Thus, Na_2_SO_4_/CaCl_2_–PAH and CaCl_2_/Na_2_SO_4_–PAH maintained a swelling ratio of less than 3% over a 48 h period, thereby functioning effectively as nonswellable hydrogels (Figure S14c). This feature is particularly advantageous for maintaining structural integrity and dimensional stability without volume expansion in aqueous environments. Biological safety, a critical requirement for wearable or implantable interface materials, was evaluated via a cytotoxicity test using NIH‐3T3 fibroblasts (Figure 3j and Figure S15). Live/Dead staining assays revealed negligible dead cell counts across all ISC–PAH samples, with cell density increasing steadily over time. Quantitative analysis confirmed that cell viability remained above 90% for all samples, with no statistically significant cytotoxicity observed (Figure 3k). These findings verify that ISC–PAHs are biologically safe, underscoring their suitability for use as practical biosensor interface materials.

### 2.4 Tailoring Electrochemical Glucose Biosensor Performance through ISC–PAHs

As previously demonstrated, ISC–PAH exhibits excellent mechanical stability, high biocompatibility, and tunable pore size, enabling regulation of analyte diffusion. When applied to enzyme‐based electrochemical glucose biosensors, these features allow stable enzyme immobilization and environment‐specific analyte diffusion control. To clearly investigate the impact of such structural tailoring on biosensor performance, enzymatic glucose biosensors—characterized by well‐defined Michaelis–Menten kinetics and incorporating an ISC–PAH diffusion‐modulating layer—were chosen as a model system owing to their clinical relevance, extensive utilization in biosensor research, and thoroughly characterized enzymatic reaction mechanisms.

**FIGURE 4:**
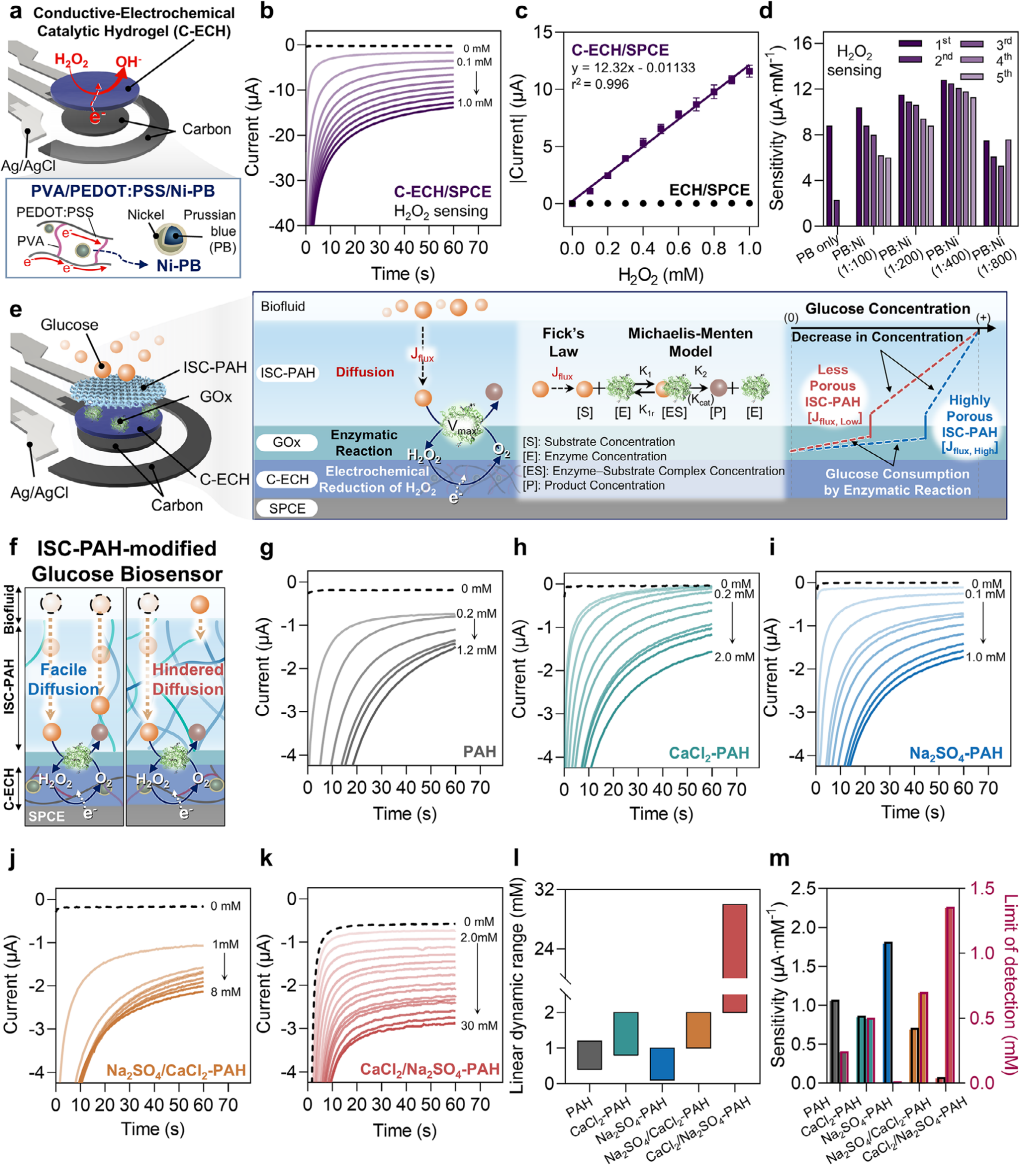
Performance evaluation of electrochemical enzymatic glucose biosensors modified with different ISC–PAH hydrogels. (a) Exploded schematic of C‐ECH‐modified SPCE. (b) Amperograms of C‐ECH/SPCE recorded from 0.1 to 1.0 mM hydrogen peroxide in 0.1 mM increments at an applied potential of −0.1 V (vs. Ag/AgCl). (c) Calibration plots for C‐ECH/SPCEs at varying hydrogen peroxide concentration. Data are presented as mean ± SD, *n* = 3. (d) Stability of the sensing response during repeated hydrogen peroxide detection as a function of the volumetric mixing ratio between Ni precursor and Prussian Blue nanoparticle dispersion. (e) Exploded schematic of an electrochemical enzymatic glucose biosensor with an ISC–PAH diffusion‐control layer, illustrating the multilayer structure (SPCE/C‐ECH/GO*_x_*/ISC–PAH) and the biosensing mechanism based on Fick's law and the Michaelis–Menten model. Glucose diffuses through the ISC–PAH layer (*J*_flux_) to the GO*_x_* layer, where it is oxidized to H_2_O_2_ and subsequently electrochemically reduced at the C‐ECH layer. The diagram illustrates the glucose concentration gradient from the bulk solution to the electrode interface, showing the decrease in concentration along the diffusion path and its consumption by the enzymatic reaction. (f) Comparative schematic of highly porous and less porous ISC–PAH layers, highlighting how differences in glucose diffusion affect the local glucose concentration (*C*_int_) and the associated biosensing mechanism. (g) Amperograms of pristine PAH‐modified glucose biosensors for glucose concentrations ranging from 0.2 to 1.2 mM with 0.2 mM increments, (h) CaCl_2_–PAH‐modified glucose biosensors for glucose concentrations ranging from 0.2 to 2.0 mM with 0.2 mM increments, (i) Na_2_SO_4_–PAH‐modified glucose biosensors for glucose concentrations ranging from 0.1 to 1.0 mM with 0.1 mM increments, (j) Na_2_SO_4_/CaCl_2_–PAH‐modified glucose biosensors for glucose concentrations ranging from 1 to 8 mM with 1 mM increments, and (k) CaCl_2_/Na_2_SO_4_–PAH‐modified glucose biosensors for glucose concentrations ranging from 2 to 30 mM with 2 mM increments. (l) Linear dynamic range, (m) sensitivity, and limit of detection of pristine PAH and ISC–PAH‐modified glucose biosensors obtained from CA measurements and calculated using calibration curves derived from the amperograms in (g–k) (*n* = 3). Each CA measurement was conducted at an applied potential of −0.1 V (vs. Ag/AgCl) for 60 s, following a 3 min incubation period to ensure the enzymatic reaction of glucose oxidase.

The glucose biosensing mechanism consists of three sequential steps: (i) glucose diffusion through the ISC–PAH outer layer to the enzyme interface, (ii) enzymatic oxidation of glucose catalyzed by glucose oxidase (GO*_x_*) to produce hydrogen peroxide (H_2_O_2_) [Equation (1)], and (iii) electrochemical oxidation or reduction of H_2_O_2_ at the electrode surface. For electrochemical detection of H_2_O_2_, we first developed a Conductive‐Electrochemical Catalytic Hydrogel (C‐ECH), designed to efficiently catalyze H_2_O_2_ electrochemical reduction [Equation (2)] and utilized as a catalytic layer (Figure 4a) [50].

(1) $$\mathrm{Glucose}+O_{2}\;\overset{\mathrm{GOx}}{\to }\;\mathrm{Gluconic}\;\mathrm{acid}+H_{2}O_{2}$$

(2) $$H_{2}O_{2}+2H^{+}+2e^{-}\;\to 2H_{2}O$$

The catalytic efficiency of the C‐ECH toward hydrogen peroxide detection was evaluated. The C‐ECH incorporated Nickel‐doped Prussian Blue (Ni‐PB) as a catalyst, while PVA and poly(3,4‐ethylenedioxythiophene):polystyrene sulfonate (PEDOT:PSS) provided mechanical flexibility and electronic conductivity (see Figures S16 and S17 for detailed information of C‐ECH). The electrochemical performance of the optimized C‐ECH was evaluated by an amperometric analysis, revealing significantly enhanced H_2_O_2_ detection compared to the hydrogel lacking PEDOT:PSS when modified on a screen‐printed carbon electrode (SPCE) (Figure 4b,c). This improvement is attributed to a synergistic effect between PEDOT:PSS and the Ni‐PB catalyst. Optimization studies identified that a PB dispersion:NiCl_2_ volume ratio of 1:400 yielded maximum sensitivity and stability (Figure 4d). Under these optimized conditions, the catalytic rate constant (*K*_cat_). of C‐ECH/SPCE for H_2_O_2_ reduction was found to be 12 s^−1^, approximately two to three times higher than previously reported values for PB‐modified, indicating a significantly faster turnover rate and consequently a more rapid electrochemical response (Figure S18) [51].

Given the high catalytic activity of C‐ECH toward H_2_O_2_, the electrochemical reduction step was unlikely to be rate‐limiting under our experimental conditions. The overall performance of the ISC–PAH‐modified glucose biosensor is therefore governed by the interplay between enzyme kinetics and substrate mass transport (Figure 4e). These characteristics directly influence the biosensor current (I), which follows the Michaelis–Menten relationship [52]

(3) $$I=\frac{I_{\max}\left[S\right]}{K_{m}+\left[S\right]}$$

where *I*_max_ is the substrate‐saturated current and *K*_m_ represents the enzyme–substrate affinity. Owing to the well‐established high catalytic turnover rate of GO*_x_*, the glucose concentration at the enzyme interface (*C*_int_) is often governed not by enzyme kinetics but by the rate of substrate diffusion through the surrounding layer. This transport of glucose through the ISC–PAH layer can be described by Fick's law [53], which defines the glucose diffusion flux (*J*_flux_):

(4) $$J_{\mathrm{flux}}=\frac{D_{\mathrm{eff}}\left(C_{\mathrm{bulk}}-C_{\mathrm{int}}\right)}{L}$$

where *D*_eff_ is the effective diffusion coefficient of the analyte within the layer, *C*_bulk_ is the bulk analyte concentration, *C*_int_ is the analyte concentration at the enzyme interface, and *L* is the thickness of the diffusion path. A reduction in *D*_eff_​ or an increase in *L* decreases *J*_flux_, lowering *C*_int_ at the enzyme interface. Consequently, the overall reaction rate in our biosensors can be determined by whichever is slower: the glucose diffusion flux through the ISC–PAH outer layer (*J*_flux_) or the maximum enzymatic turnover rate (*V*_max_). The *V*_max_ is determined by enzyme loading and intrinsic catalytic activity, which were maintained constant across all biosensor configurations by standardizing GO*_x_* loading and catalytic properties during fabrication [52]. Likewise, the use of an identical C‐ECH transducer layer ensured that *I*_max_ remained equivalent among biosensors. Under these controlled conditions, the observed differences in apparent *K*_m_, sensitivity, and linear range are most reasonably ascribed to variations in mass transport (*J*_flux_) induced by structural changes in the ISC–PAH layer.

Structural adjustments to ISC–PAH directly modulate the glucose concentration at the electrode interface (*C*_int_), enabling fine‐tuning of the apparent Michaelis–Menten constant (*K*_m,app_) to optimize biosensor sensitivity and linear dynamic range for specific analytical environments. A highly porous ISC–PAH decreases diffusion resistance, raises *C*_int_ of glucose, and lowers *K*_m,app_, resulting in high sensitivity in low‐substrate environments; conversely, a denser ISC–PAH increases diffusion resistance, lowers *C*_int_ of glucose, and raises *K*_m,app_, delaying enzyme saturation and extending the linear dynamic range—particularly advantageous at high glucose concentrations where oxygen depletion can occur. GO*_x_* was immobilized onto optimized C‐ECH/SPCE electrodes, followed by modification with ISC–PAH layers of differing porosity to systematically regulate *J*_flux_ of glucose. GO*_x_* loading and catalytic properties were standardized during fabrication to ensure that any observed performance differences arose solely from ISC–PAH‐mediated modulation of diffusion characteristics (Figure 4f). The resulting modified electrodes were comprehensively assessed to determine their glucose biosensing performance.

To assess whether ISC–PAH can function as an outer diffusion‐control layer to modulate sensitivity and linear range, ISC–PAH was coated onto GO*_x_*/C‐ECH/SPCE. Glucose sensing performance was then quantified via chronoamperometry. In low‐glucose environments, pristine PAH and ISC–PAHs with single‐ion (Ca^2+^ or SO_4_^2−^) treatments featuring tailored porous microstructures were employed as diffusion‐controlling layers (Figure 4g–i). Among these, Na_2_SO_4_–PAH‐modified glucose biosensor achieved the highest sensitivity (with a regression equation, *y* = 1.813x − 0.08624, *R* ^2^ = 0.995) with a linear range of 0.1–1 mM (Figure S19a). Varying the Na_2_SO_4_ treatment duration progressively increased sensitivity and shifted the linear range toward lower concentrations, with the effect saturating at 24 h (Figure S19b). In high‐glucose environments, ISC–PAHs with sequential ion treatments (Ca^2+^ and SO_4_^2−^) yielding dense networks were used as diffusion‐controlling layers. The glucose biosensor modified with CaCl_2_/Na_2_SO_4_–PAH showed reduced sensitivity but substantially extended linear sensing response ranging from 2 to 30 mM, whereas the biosensor modified with Na_2_SO_4_/CaCl_2_–PAH remained limited to the lower glucose concentration range (Figure 4j,k and Figure S19c). This difference arises due to the critical role of the ion treatment sequence during biomineralization. When Na_2_SO_4_ is introduced first, it promotes porous structure formation and increased analyte diffusion rates favorable for high sensitivity at lower concentrations. In contrast, applying CaCl_2_ first initiates immediate crosslinking and biomineralization, yielding a denser and more tightly crosslinked network that restricts diffusion. This diffusion control process enabled by dense networks formed after ion chelation and biomineralization effectively regulates glucose diffusion, thereby mitigating oxygen depletion at high glucose concentrations. The linear dynamic range was also observed to increase progressively with biomineralization time, reaching a plateau at 24 h (Figure S19d).

The porous Na_2_SO_4_‐treated ISC–PAH increased *D*_eff_, *J*_flux_, and *C*_int_ of glucose while lowering *K*_m,app_, yielding high sensitivity but a narrow low‐glucose range. In contrast, the dense CaCl_2_/Na_2_SO_4_‐treated ISC–PAH decreased *D*_eff_, *J*_flux_, and *C*_int_ of glucose while raising *K*_m,app_, reducing sensitivity but markedly extending the linear range for high‐glucose detection. This trend aligns with quantitative analysis of *K*_m, app_, which showed that the porous Na_2_SO_4_–PAH exhibited a low *K*_m, app_ of 1.49 mM, indicative of enhanced glucose diffusion, whereas the dense CaCl_2_/Na_2_SO_4_–PAH displayed a significantly higher *K*_m, app_ of 18.7 mM, reflecting restricted diffusion. These results indicate that apparent enzyme kinetics can be adjusted through structural control of the outer diffusion layer, which serves as a key mechanism for tailored biosensor design.

A comprehensive comparison of the glucose sensing performance across various ISC–PAH‐modified glucose biosensors revealed a broadening of the linear dynamic range with variation in the outer diffusion‐control membrane. The order was Na_2_SO_4_–PAH < PAH < CaCl_2_–PAH ≈ Na_2_SO_4_/CaCl_2_–PAH << CaCl_2_/Na_2_SO_4_–PAH (Figure 4l). Among the five ISC–PAH‐modified glucose biosensors, the Na_2_SO_4_–PAH‐modified glucose biosensor exhibited the highest sensitivity (1.813 µA mM^−^^1^) and the lowest limit of detection (LOD, 15.4 µM), whereas the CaCl_2_/Na_2_SO_4_–PAH‐modified glucose biosensor exhibited the lowest sensitivity (0.077 µA mM^−^^1^) and the highest LOD (1.36 mM) (Figure 4m). These tendencies are consistent with the pore‐size dependent modulation of the effective diffusivity of the redox probe discussed in Figure 2a,b, and g. Accordingly, the Na_2_SO_4_–PAH membrane is optimal for low‐glucose concentration detection, while the CaCl_2_/Na_2_SO_4_–PAH membrane is better suited for high concentration glucose detection.

Overall, these findings indicate that ion‐mediated engineering provides a viable strategy for modulating the sensitivity and linear dynamic range of a biosensor within a single PAH‐based material platform, thereby supporting adaptation to varying analytical requirements. Whereas previous studies had to design and optimize separate polymer membranes for specific purposes (high S/N ratio [21] or wide range [54]), requiring different chemical compositions, crosslinking methods, or templating strategies for each application, our strategy enables customized interface properties simply by altering ion‐treatment conditions, thereby enhancing clinical adaptability without the need for complex synthesis. This versatility is further validated by the comparative analysis (Table S4, Supporting Information), which demonstrates that ISC–PAHs uniquely integrate dual adaptability for both sweat‐compatible low‐concentration detection and ISF‐compatible high‐concentration monitoring. In addition, although mechanical rigidity is a critical parameter for hydrogel‐based biosensors, as hydrogels must maintain structural stability under deformation when applied to skin or tissue, previous studies have typically addressed this aspect only qualitatively, without quantitative validation. In contrast, the ISC–PAH system presented here simultaneously achieves physiologically relevant mechanical robustness and antifouling capability, thereby overcoming the limitations of conventional hydrogel‐based biosensors and demonstrating superior multifunctional performance for practical biosensing applications.

### 2.5 Wearable Electrochemical Biosensor Integrated with Na_2_SO_4_–PAH Interface for Sweat Glucose Monitoring

**FIGURE 5:**
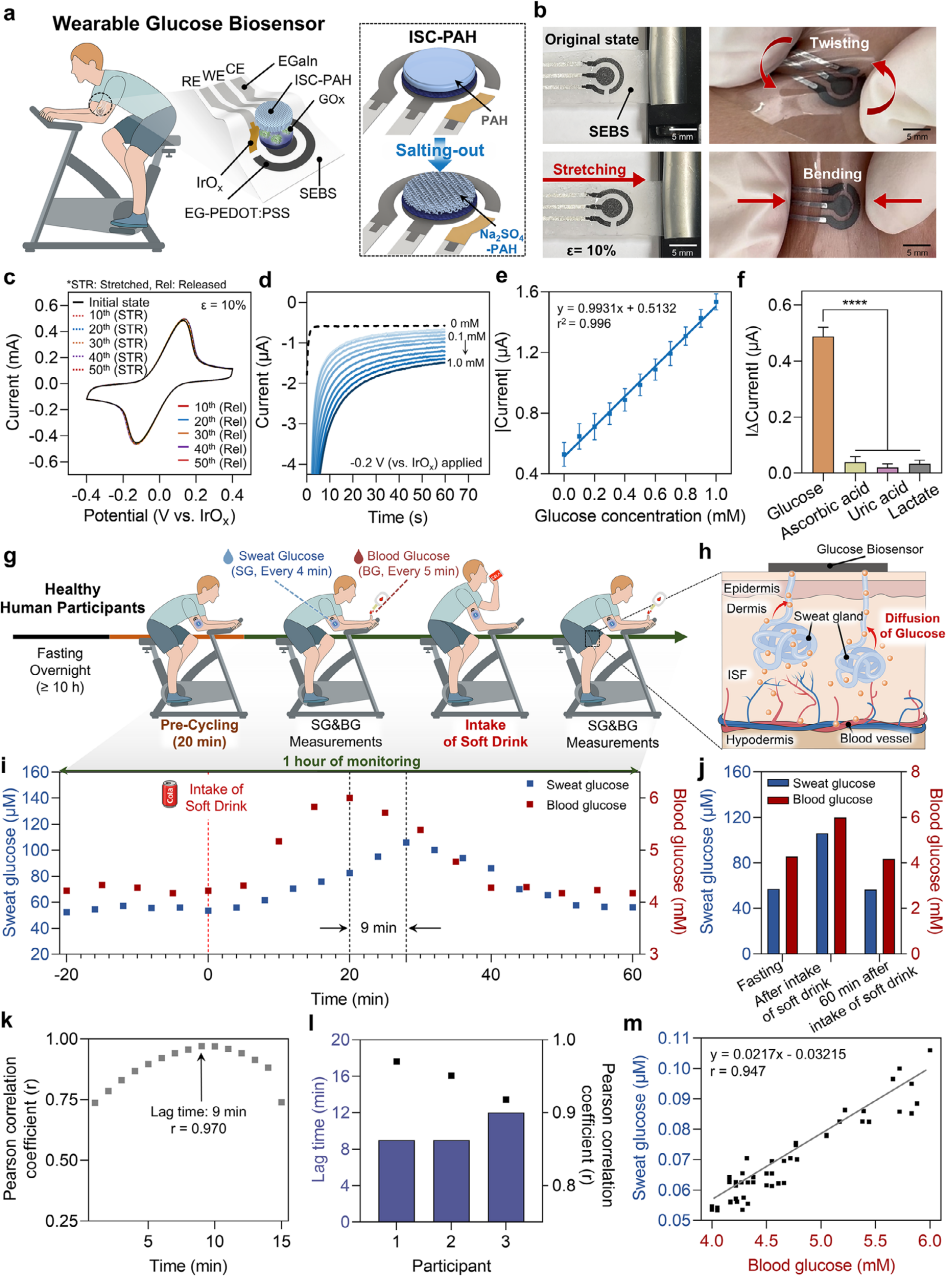
Wearable electrochemical glucose biosensor with Na_2_SO_4_–PAH interface for continuous SG monitoring in a human pilot study. (a) Schematic illustration of the developed wearable electrochemical glucose biosensor patch, built on a soft electrode platform comprising an EGaIn current collector, EG‐PEDOT:PSS electrodes, and an IrO*_x_* reference electrode on a SEBS substrate, and integrated with a C‐ECH catalytic layer, a GO*_x_* enzyme layer, and a highly porous Na_2_SO_4_–PAH diffusion‐control layer. (b) Photographs demonstrating mechanical deformations (stretching, twisting, and bending) applied to the biosensor patch. (c) Cyclic voltammograms of the biosensor patch under repeated 10% stretching–release cycles, recorded in a 5 mM K_3_Fe(CN)_6_/K_4_Fe(CN)_6_ solution. (d) Amperometric responses of Na_2_SO_4_–PAH‐modified biosensor patch for glucose concentrations from 0.1 to 1.0 mM with 0.1 mM increments. (e) Corresponding calibration plots and (f) selectivity for 0.5 mM glucose against ascorbic acid at 0.01 mM, uric acid at 0.05 mM, and lactate at 5 mM, based on the change in absolute current values. Data are presented as mean ± SD, *n* = 3. (g) Protocol of the human pilot study in healthy participants, in which SG and BG were monitored during cycling, with intake of a soft drink in between. (h) Schematic illustration showing the passive diffusion pathway of glucose from the bloodstream into eccrine sweat. (i) Continuous monitoring results of SG and BG in a representative participant. (j) Comparison of SG and BG measured in the fasting state and at 30 and 60 min after soft drink intake. (k) Pearson's correlation coefficient between SG and BG as a function of lag time for a representative participant. (l) Lag time and Pearson's correlation coefficient, and (m) scatter plot showing the relationship between SG and BG levels across all participants (*n* = 3).  All amperometric measurements were conducted at an applied potential of −0.2 V (vs. IrO*_x_*) for 60 s, following a 3 min incubation period to ensure enzymatic reaction of GO*_x_*. (*p < 0.05, **p < 0.01, ***p < 0.001, ****p < 0.0001); ns, not significant.

To demonstrate the practical value of the established ion‐mediated engineering, a wearable electrode platform‐composed of a three‐electrode system based on Elastic Graphite‐filled electrochemical PEDOT:PSS (EG‐PEDOT:PSS) and an IrO*_x_* reference electrode, which is a well‐known biocompatible reference electrode material, on a Stretchable Elastomer styrene–ethylene/butylene–styrene block copolymer (SEBS) substrate was first constructed. The final biosensor was then developed by integrating the Na_2_SO_4_–PAH, optimized for low‐concentration glucose detection, as the outermost interface with GO*_x_* and C‐ECH (Figure 5a). As wearable sweat biosensors must flexibly respond to the wearer's movements while sensitively detecting glucose at micromolar concentrations in sweat, the underlying EG‐PEDOT:PSS electrodes must exhibit mechanical durability and stable electrochemical operation.

Therefore, prior to evaluating the biosensor performance, the mechanical and electrochemical properties of the developed wearable electrode platform were verified. The core components of the platform, the EG‐PEDOT:PSS ink and the IrO*_x_* reference electrode, were developed to possess excellent printability, fast electron transfer rates, and long‐term stability (see Figures S20–S26 for detailed optimization). Prior to analytical testing, the printed electrode set was verified to maintain both structural and electrochemical stability under mechanical deformation relevant to wearable operation, thereby ensuring that subsequent performance originated from the ISC–PAH layer rather than from effects within the underlying electrode platform. The fully fabricated electrode platform was confirmed to maintain excellent mechanical integrity without cracks or structural damage under various deformations, including bending, twisting, and 10% tensile strain (Figure 5b). Moreover, the electrochemical stability also showed stable responses during and after 50 repeated cycles of 10% tensile strain (Figure 5c).

Using the previously validated highly porous ISC–PAH (Na_2_SO_4_–PAH), designed to achieve robust mechanical properties and facilitate efficient glucose diffusion for enhanced sensitivity at low glucose concentrations, the final biosensor was completed by sequentially coating the C‐ECH, GO*_x_*, and Na_2_SO_4_–PAH, and its analytical performance was systematically evaluated. The completed biosensor exhibited a highly linear current response to glucose in the 0.1–1 mM concentration range with the regression equation of *y* = 0.9931x + 0.5132 (*R* ^2^ = 0.995, *n* = 3) (Figure 5d,e). Due to its operating principle of selectively reducing H_2_O_2_, the biosensor demonstrated excellent selectivity for glucose against common oxidizable interfering substances found in sweat, such as ascorbic acid, uric acid, and lactate (Figure 5f).

Finally, to validate the practical efficacy of the developed Na_2_SO_4_–PAH‐based wearable glucose biosensor, a human pilot study was conducted with four healthy participants. The participants, in a fasting state, wore the biosensor patch on their upper arm, and dynamic changes in blood glucose (BG) and sweat glucose (SG) were induced through cycling and the intake of a sugar‐sweetened beverage, while BG and SG were continuously monitored (Figure 5g). Glucose concentrations in sweat reflect BG levels via passive diffusion from the blood (Figure 5h). Accordingly, BG was simultaneously monitored to ensure the accuracy of SG detection, consistent with previous studies employing noninvasive wearable biosensors for glucose monitoring in biofluids. Results from the pilot study showed a clear trend where the SG concentration closely tracked the dynamic changes in BG concentration with a time lag (Figure 5i,j and Figure S27). For one representative participant, BG peaked 20 min after the beverage intake, while SG peaked at approximately 30 min, with both values returning to baseline levels due to insulin response of the body. To quantitatively analyze the correlation, the lag time for each participant was determined by calculating the Pearson correlation coefficient, resulting in a lag‐time range of 9–12 min at the highest correlation (Figure 5k,l). After lag time correction, all participants exhibited consistently strong correlations between SG and BG, with similar proportional relationships across individuals (Figure S28a,b). A linear mixed‐effects (LME) analysis of participant‐specific correlations and SG/BG partition ratios confirmed no significant inter‐individual differences (*p* > 0.05), confirming that the pooled correlation faithfully represents the group‐level relationship (Figure 5m). The SG/BG partition ratio remained statistically stable across participants and showed a consistent dynamic trajectory over time, characterized by a transient rise and recovery following glucose intake (Figure S28c,d), reflecting physiological regulation rather than participant‐specific fluctuations. The pooled SG/BG partition ratio (≈0.021) was comparable to the previously reported physiological range (10^−3^–10^−2^), thereby confirming the physiological relevance and reproducibility of the observed SG–BG coupling [55, 56]. To further evaluate the clinical accuracy of the sweat‐based glucose predictions, a Parkes Error Grid (PEG) analysis was performed using 54 paired SG–BG data points collected from human subjects (Figure S29). All data points fell within Zone A, the most clinically acceptable region, demonstrating the biosensor's high predictive accuracy and confirming its strong potential as a noninvasive glucose monitoring platform with clinically safe performance.

The wearable glucose biosensor demonstrated in this work successfully monitored the dynamic changes of glucose in human sweat. Unlike many conventional wearable sweat biosensors that sacrifice mechanical durability to achieve high sensitivity [57, 58], or introduce complex structures to ensure durability [59], the Na_2_SO_4_–PAH interface in this study simultaneously achieves high diffusivity while maintaining intrinsic mechanical robustness of the material through the salting‐out process. Resolving this trade‐off between performance and durability within a single material represents a significant advancement, potentially enabling reliable detection of glucose at low concentrations relevant to sweat‐based wearable biosensing applications.

### 2.6 Implantable Electrochemical Biosensor Integrated with CaCl_2_/Na_2_SO_4_–PAH Interface for Interstitial Fluid Glucose Monitoring

**FIGURE 6:**
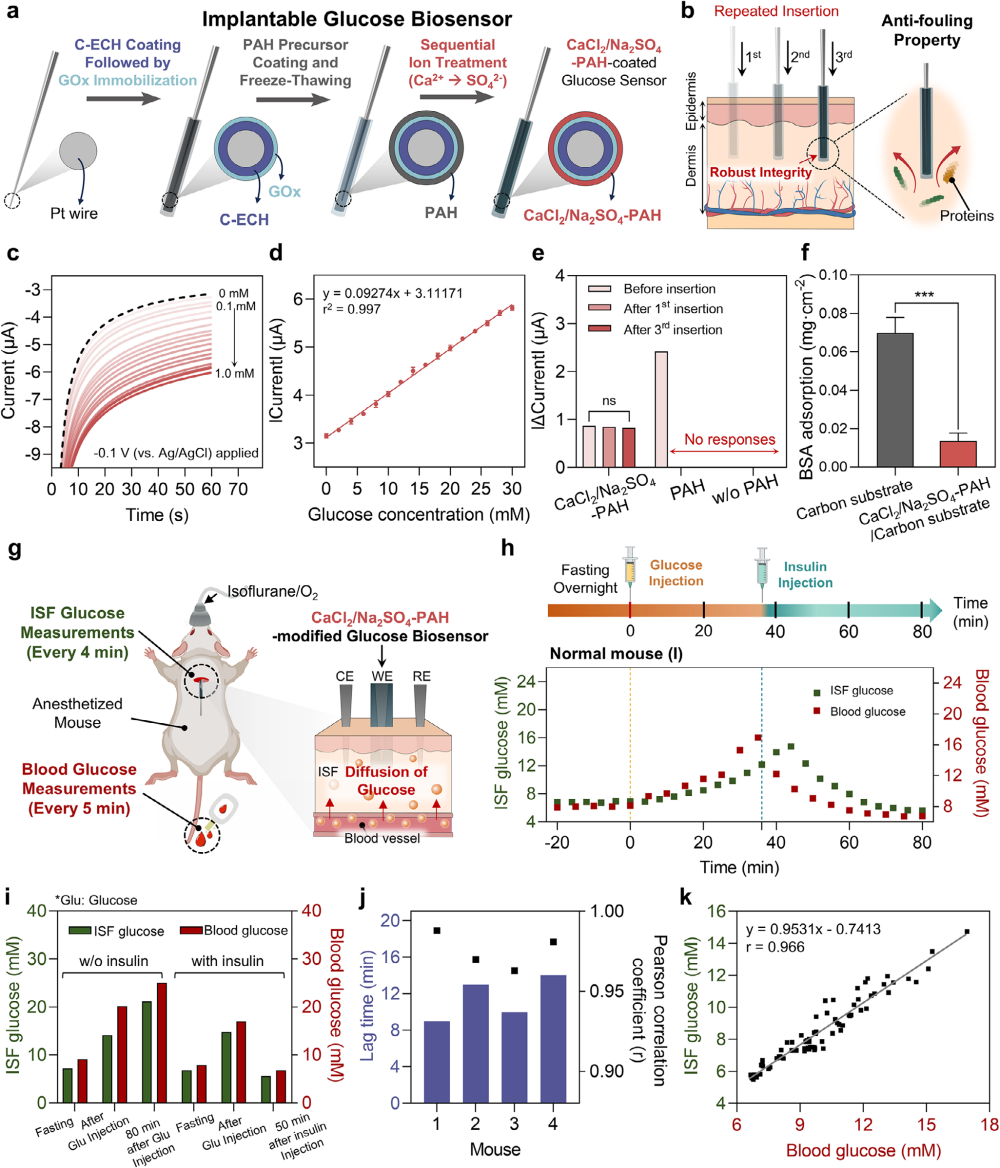
Implantable electrochemical glucose biosensor with CaCl_2_/Na_2_SO_4_–PAH interface for continuous IG monitoring in normal mice. (a) Schematic illustration displaying the fabrication process of CaCl_2_/Na_2_SO_4_–PAH/GO*_x_*/C‐ECH/Pt electrode, incorporating a C‐ECH catalytic layer, GO*_x_* enzyme layer, and a sequential ion‐treated CaCl_2_/Na_2_SO_4_–PAH diffusion‐control layer. (b) Schematic showing the mechanical integrity and antifouling performance of the developed implantable glucose biosensor after repeated insertion–withdrawal cycles. (c) Amperometric responses of the CaCl_2_/Na_2_SO_4_–PAH‐modified implantable biosensor for glucose concentrations from 2 to 30 mM with 2 mM increments, and (d) corresponding calibration plots based on absolute current responses. Data are presented as mean ± SD, *n* = 3. (e) Bar graph showing CA responses to 1 mM glucose for implantable biosensors without hydrogel coating, with PAH coating, and with CaCl_2_/Na_2_SO_4_–PAH coating after repeated insertion–withdrawal cycles into phantom skin. (f) Quantification of nonspecific BSA adsorption on carbon substrate and CaCl_2_/Na_2_SO_4_–PAH‐modified carbon substrate obtained from UV–vis spectroscopy. Data are presented as mean ± SD, *n* = 3. (g) Schematic illustration of glucose diffusion from the bloodstream into ISF and of an anesthetized mouse with insertion of the implantable biosensor. h) Protocol for in vivo study monitoring IG and BG in normal mice and representative continuous monitoring profiles of IG and BG in a mouse. (i) Comparison of IG and BG levels in mice without and with insulin injections. For control mouse (without insulin), IG and BG concentrations were obtained at baseline and 35 min after glucose injection and again at 80 min postinjection. For insulin‐injected mouse, IG and BG concentrations were obtained at the same time points with an additional measurement taken 50 min after insulin administration. (j) Lag time and Pearson's correlation of mice (*n* = 4). (k) Scatter plot showing the relationship between IG and BG across all mice (*n* = 4). All amperometric measurements were conducted at an applied potential of −0.1 V (vs. Ag/AgCl) for 60 s, following a 3 min incubation period to ensure the enzymatic reaction of GO*_x_*. (*p < 0.05, **p < 0.01, ***p < 0.001, ****p < 0.0001); ns, not significant.

Following the development of the ISC–PAH‐modified wearable biosensor, this study was expanded to minimally invasive glucose monitoring using ISF. ISF‐based sensing offers distinct advantages, providing a high correlation with BG while markedly reducing the risk of adverse biological reactions, such as bleeding, pain, and infection compared to fully invasive biosensors inserted directly into blood vessels. As ISF reflects changes in BG more accurately and rapidly than other noninvasive biofluids like sweat or tears, ISF‐based glucose biosensors must reliably operate within the physiological glucose concentration range—a low‐to‐tens of millimolar concentration—comparable to that of BG. Accordingly, to achieve the linear and stable detection of high‐concentration glucose (>10 mM) in ISF, the CaCl_2_/Na_2_SO_4_–PAH, previously verified to have high diffusion resistance and excellent mechanical robustness, was applied as the diffusion‐limiting outer layer of an implantable glucose biosensor. The implantable glucose biosensor was fabricated by sequentially dip‐coating a platinum (Pt) wire electrode with the C‐ECH, GO*_x_*, and ISC–PAH, followed by the sequential ion treatments to form the final Pt/C‐ECH/GO*_x_*/CaCl_2_/Na_2_SO_4_–PAH configurations, which exhibit robust mechanical stability and antifouling properties suitable for implantation (Figure 6a,b and Figure S30).

The electrochemical characteristics and H_2_O_2_ sensing performance of the fabricated electrode was evaluated in vitro. Distinct oxidation/reduction peaks of Ni–PB were observed in developed biosensor (Figure S31). CA measurements confirmed that the biosensor exhibited a wide linear dynamic range of 2–30 mM for glucose, with a regression equation of *y* = 0.09234x + 3.11171 (*R* ^2^ = 0.997, *n* = 3) (Figure 6c,d). Additionally, the biosensor exhibited excellent selectivity for 5 mM glucose even in the presence of common interfering substances found in ISF, such as ascorbic acid, uric acid, and lactic acid (Figure S32). To assess mechanical robustness and antifouling performance under realistic conditions, the electrode was tested using a “phantom skin” with tissue‐like elasticity, and the setup was immersed in a protein‐containing solution (5 mg mL^−1^ of BSA) mimicking biofluid conditions (Figure 6e and Figure S33a,b). GO*_x_*/C‐ECH/Pt electrodes without an ISC–PAH coating exhibited no glucose biosensing response even before penetrating the phantom skin due to the absence of an effective enzyme immobilization layer that led to enzyme detachment. Pristine‐PAH/GO*_x_*/C‐ECH/Pt electrodes showed a rapid decline in glucose response after phantom skin penetration attributed to their limited mechanical strength, which was readily compromised by external stress and subsequent enzyme loss (Figure S33c). In contrast, CaCl_2_/Na_2_SO_4_–PAH/GO*_x_*/C‐ECH/Pt electrodes maintained a stable glucose biosensing response even after three consecutive piercing cycles in the presence of BSA, demonstrating their effective role as a protective layer against mechanical stress (Figure S33d). To further characterize the dynamic stability and reproducibility of the CaCl_2_/Na_2_SO_4_–PAH‐modified implantable biosensor, repetitive glucose‐cycling tests were performed in vitro using artificial interstitial fluid (a‐ISF). The glucose concentration was cycled between 0 and 20 mM in 4 mM increments to simulate physiological fluctuations. The biosensor accurately tracked these repeated up‐down transitions, and the end‐of‐step currents at each concentration showed coefficients of variation (CVs) below 3%, confirming excellent reproducibility and operational stability (Figure S34a,b). Additionally, in the 4–16 mM glucose range, comparing the current responses under concentration increase (up‐step) and decrease (down‐step) conditions differed by less than 3%, indicating symmetric response characteristics during glucose concentration cycling. These results suggest that the signal fully recovered within the 4‐min measurement interval, even during the concentration‐decrease phase (Figure S34c).

Moreover, its antifouling property was confirmed by UV–vis spectroscopy, which showed that residual BSA in the solution increased by approximately 4.76% for the coated substrate compared to the uncoated one, confirming suppressed protein adsorption (Figure 6f). To further investigate the long‐term antifouling behavior, fluorescence imaging using FITC–BSA was performed (Figure S35a,b). The hydrogel‐coated surface exhibited negligible fluorescence up to Day 5, indicating that protein adsorption was effectively suppressed. However, after 7 days of immersion, the hydrogel surface underwent gradual rearrangement under prolonged exposure to the protein‐containing medium, leading to partial exposure of protein‐binding sites and limited surface adsorption. In contrast, electrochemical measurements demonstrated that the diffusivity of redox species and signal stability were maintained over 14 days of continuous measurement (Figure S35c). These combined observations suggest that, although minor protein adsorption occurs beyond 1 week, it remains confined to the outer hydrogel layer without penetrating toward the electrode interface and does not significantly affect analyte transport or sensor performance. This observation is further supported by SEM images of CaCl_2_/Na_2_SO_4_–PAH after prolonged immersion in a‑ISF. These images show that, although the average crystal size gradually decreases over 14 days, the internal mineralized network retains a high crystal density (Figure S36). This demonstrates that the hydrogel preserves its compact structure, maintaining restricted analyte diffusion and mechanical robustness even after extended physiological exposure due to the biomineralized CaSO_4_. Collectively, CaCl_2_/Na_2_SO_4_–PAH provides strong initial antifouling protection that gradually weakens after extended exposure yet continues to ensure stable analyte diffusion toward the electrode interface. Unlike membranes typically employed in implantable biosensors, which require additional antifouling coatings or rigid housings, our biomineralized CaCl_2_/Na_2_SO_4_–PAH integrates diffusion control, antifouling, and mechanical protection within a single layer.

To validate the in vivo efficacy of the developed biosensor, continuous monitoring of ISF glucose (IG) was performed by subcutaneously implanting the biosensor in healthy 7‐week‐old male mice (Figure 6g). The study was designed to determine whether the developed glucose biosensor could detect both the rise and fall of BG levels by sequentially injecting glucose and, after a defined time interval, insulin into healthy mice (Figure 6h). In a representative trial, the developed implantable glucose biosensor successfully captured a sharp increase in glucose concentration immediately after intraperitoneal glucose injection, followed by a distinct decline driven by insulin‐mediated glucose uptake. In contrast, no decrease was observed in a control mouse that did not receive insulin (Figure S37), likely due to anesthesia‐induced insulin resistance. These results demonstrate the biosensor's capability to sensitively track glucose dynamics and reliably reflect insulin‐mediated metabolic responses.

In a representative mouse, the corresponding plots of IG and BG measurements at different experimental time points clearly illustrate a sharp elevation in both BG and IG concentrations immediately following glucose administration (Figure 6i). In the absence of insulin injection, glucose levels remained persistently elevated. In contrast, subsequent insulin administration led to a distinct decrease in both BG and IG concentrations, effectively returning them toward baseline levels. Furthermore, the biosensor's dynamic responsiveness was confirmed by real‐time detection of glucose metabolism following external insulin administration. Across all four mice, the IG consistently followed the BG trend (Figure S38), and quantitative analysis confirmed a strong positive correlation between the two values, with a time lag range of 9–14 min and a high IG/BG partition ratio of 0.9531 (Figure 6j,k). The robustness of these results was further analyzed through statistical analysis (Figures S39 and S40). To further assess the clinical reliability of the developed biosensor, PEG analysis was performed using 74 paired IG and BG values collected from in vivo measurements (Figure S41). All data points fell within the clinically acceptable Zones A and B, with 98.6% in Zone A and 1.4% in Zone B. The predominance of data points within Zone A confirms the biosensor's high clinical accuracy and supports its suitability for safe therapeutic glucose monitoring.

Consequently, the CaCl_2_/Na_2_SO_4_–PAH outer layer was confirmed as a multifunctional interface that effectively endures mechanical stress, stably controls analyte flux, and possesses excellent antifouling properties in a simulated implantation environment. These results confirm that the “purpose‐driven design” strategy, where microstructures of hydrogel are precisely tailored through ion‐mediated control, effectively achieves controlled analyte diffusion, mechanical robustness, and long‐term antifouling performance. This approach directly fulfills the requirements in invasive environments, underscoring its potential as a versatile and practical platform for advanced biosensing applications. Implantable biosensors require effective prevention of foreign body reactions and protein fouling to ensure long‐term biosensor stability and clinical applicability. The CaCl_2_/Na_2_SO_4_–PAH interface developed in this study enabled stable and continuous monitoring of dynamic glucose changes, such as insulin‐induced fluctuations, in a in vivo environment. This compelling result conclusively demonstrates that the proposed ion‐mediated structural engineering is a highly effective framework for developing next‐generation implantable biosensors.

## 3 Conclusion

This study introduces a novel “ion‐mediated structural engineering” as a versatile strategy for fine modulation of diffusion control in electrochemical biosensors, successfully addressing the long‐standing trade‐off between mechanical robustness and analyte diffusivity. By combining multiple ion–polymer interactions within a single PAH system, including salting‐out, ion chelation, and sequence‐dependent biomineralization, we achieved bidirectional and quantitative tunability of the material's microstructure (pore size: 65 nm to 2.5 µm), mechanical properties (elastic modulus: 50–140 kPa), and diffusion characteristics. This strategy successfully decoupled inversely correlated parameters, enabling the rational design of hydrogel interfaces optimized for specific biosensing environments. Utilizing ISC–PAH as outer diffusion‐control layers enabled carefully tailored modulation of glucose diffusion, allowing both high‐porosity and low‐porosity architectures to meet distinct sensing requirements. The highly porous ISC–PAH enhanced analyte transport and sensitivity at low glucose concentrations, while the dense, low‐porosity ISC–PAH provided diffusion restriction for stable detection across a wide range of glucose levels. The practicality and versatility of this platform were validated through two purpose‐driven glucose biosensors. For wearable applications, a porous Na_2_SO_4_–PAH hydrogel based on a high‐flux diffusion strategy achieved high sensitivity (1.813 µA mM^−^^1^) and a low detection limit (15.44 µM) with reliable performance confirmed in a pilot human study. For implantable biosensors, a dense CaCl_2_/Na_2_SO_4_–PAH hydrogel employing a diffusion‐limiting strategy enabled continuous glucose monitoring over a wide glucose concentration range (2–30 mM) in interstitial fluid, with strong mechanical and antifouling stability verified in vivo.

To comprehensively benchmark the overall performance of ISC–PAH against existing hydrogel‐based sensing materials, we constructed a comparative radar plot (Figure S42). Unlike previous systems, which typically excelled in only one or two parameters at the expense of others, ISC–PAH exhibits a well‐balanced performance across multiple key properties, including tensile strength, Young's modulus, elongation at break, fatigue resistance, controlled diffusivity, and antifouling capability.

Overall, this work establishes ISC–PAH as a single hydrogel platform for tunable, high‐performance sensing via simple ionic modulation. It further offers an adaptable framework for next‐generation biointerfaces that resolves the trade‐off between mechanical robustness and molecular diffusion. The insights provided in this work establish a generalized and scalable design principle with broad implications not only for advanced enzymatic and affinity‐based biosensors but also for diverse fields, such as soft robotics, tissue engineering, and controlled drug delivery. This approach holds considerable promise for accelerating the development of robust, reliable, and clinically adaptable wearable and implantable bioelectronics, paving the way for new possibilities in personalized medicine and real‐time health monitoring. Given these insights, future work will focus on elucidating the ion‐sequence‐dependent structural dynamics of ISC–PAH using in situ methods capable of resolving ion‐diffusion behavior and structural reorganization. Such mechanistic clarification will further refine our understanding of network evolution under ionic modulation and guide the continued optimization of ISC–PAH interfaces for diverse biosensing applications. In addition, extended long‐term stability evaluations under physiologically relevant conditions and in vivo environments will be pursued to establish the durability required for next‐generation wearable and implantable biosensing applications.

## 4 Experimental Section

### 4.1 Materials

The following chemicals were used without further purification: poly(vinyl alcohol) (PVA, Mw 89 000–98 000), sodium alginate (low viscosity), glucose oxidase (GOx, from *Aspergillus niger*, ≥100 000 units g^−^^1^ solid), potassium phosphate monobasic (≥99.0%), potassium phosphate dibasic (≥98.0%), potassium hexacyanoferrate(III) (≥ 99.0%), potassium hexacyanoferrate(II) trihydrate (≥99.95%), D‐(+)‐glucose (≥99.5%), L‐ascorbic acid (≥99.0%), uric acid (≥99.0%), L‐(+)‐lactic acid (≥99.0%), sodium sulfate (Na_2_SO_4_, ≥ 99.0%, anhydrous, powder), calcium chloride (CaCl_2_, ≥ 99.0%, anhydrous), ammonium chloride (NH4Cl, 99.0%), magnesium chloride (MgCl_2_, ≥98%), potassium chloride (KCl, 99.0%–100.5%), nickel chloride (NiCl_2_, 98%), graphite (<20 µm, powder), dimethyl sulfoxide (DMSO, ≥99.9%), iridium(IV) chloride hydrate (≥99.9%), hydrogen peroxide (30 wt%), oxalic acid (≥99.9%), hydrochloric acid (37%), potassium carbonate anhydrous (99.99%), and fluorescein isothiocyanate‐dextran (4, 20, and 40 kDa), all purchased from Sigma‐Aldrich (St. Louis, MO). PEDOT:PSS suspension (Clevios PH1000) was obtained from Heraeus Electronic Materials (Hanau, Germany). Poly(styrene‐*b*‐(ethylene‐*co*‐butylene)‐*b*‐styrene) (SEBS) solution (Tuftec H1221, 13 wt% in toluene) was obtained from Asahi Kasei (Tokyo, Japan). Waterborne polyurethane (WPU) was provided by Youngjin Texchem (Daegu, South Korea). Silver/silver chloride (Ag/AgCl) paste and normal carbon paste were purchased from Sun Chemical (Parsippany, NJ). Polydimethylsiloxane (PDMS, Sylgard 184) was supplied by Dow Corning (Midland, MI). Liquid metal (LM, 75 wt% gallium and 25 wt% indium) was purchased from Changsha Rich Nonferrous Metals Co., Ltd. (Hunan, China). Deionized (DI) water for all solutions was purified with a Milli‐Q water purification system (Millipore). Unless otherwise mentioned, all solutions were dissolved in phosphate buffer solution (PBS, 50 mM, pH 7.0).

### 4.2 Preparation of Ion‐Mediated Structurally Controlled Poly(vinyl alcohol)‐Alginate Hydrogels (ISC–PAH)

Poly(vinyl alcohol)–Alginate hydrogels (PAH) were prepared by following the procedure described below. First, 3 g of PVA was dissolved in 27 mL of DI water at 120°C with stirring for 30 min until completely dissolved. Subsequently, 0.6 g of sodium alginate was subsequently added and stirred under the same conditions to achieve a homogeneous precursor solution, which was then poured into a mold and stored in a freezer at −20°C for 20 h and thawed at 25°C for 3 h to form pristine PAH. Then, pristine PAHs were immersed in salt solutions to obtain ISC–PAHs, with porous structures tailored by the specific ionic treatments as follows: (i) CaCl_2_–PAH: 2.0 M CaCl_2_ solution for 12 h, (ii) Na_2_SO_4_–PAH: 1.5 M Na_2_SO_4_ solution for 24 h, (iii) Na_2_SO_4_/CaCl_2_–PAH: 1.5 M Na_2_SO_4_ solution for 24 h followed by 2.0 M CaCl_2_ solution for 12 h, and (iv) CaCl_2_/Na_2_SO_4_–PAH: 2.0 M CaCl_2_ solution for 12 h followed by 1.5 M Na_2_SO_4_ solution for 24 h. Residual ions were removed by rinsing with DI water after immersion in the salt solution.

### 4.3 Microstructural and Material Characterization of ISC–PAHs

The microstructure of freeze‐dried pristine PAH and various ISC–PAHs was examined to assess surface morphology and porosity. In addition, to investigate the growth behavior of CaSO_4_ crystals, hydrogels were imaged at multiple time points within the PAH matrix during the biomineralization process. For CaCl_2_/Na_2_SO_4_–PAH, pristine PAH was first treated with 2 M CaCl_2_ for 12 h, and then immersed in 1.5 M Na_2_SO_4_ solution, and images were acquired at specific time intervals during the Na_2_SO_4_ immersion (*t* = 0, 30 min, 1, 3, 6, 12, and 24 h). Conversely, for Na_2_SO_4_/CaCl_2_–PAH, hydrogels were pretreated with 1.5 M Na_2_SO_4_ for 24 h and then transferred to 2 M CaCl_2_ solution, with SEM imaging performed at the same time points as a function of CaCl_2_ exposure. The stability of the grown CaSO_4_ crystals was further investigated by immersing the CaCl_2_/Na_2_SO_4_–PAH and Na_2_SO_4_/CaCl_2_–PAH in artificial interstitial fluid (a‐ISF), followed by freeze‐drying and imaging at different time points (*t* = 0, 3, 6, 12, 24, and 48 h). All images were obtained using scanning electron microscopy (SEM) performed with a field‐emission SEM (JSM‐IT500HR, JEOL, Japan) after platinum coating at an accelerating voltage of 15.0 kV. The pore sizes of the PAH and various ISC–PAHs were analyzed using a porosimeter (PM33GT, Quantachrome, USA). Analyses of functional groups and bonding environments were conducted using Fourier‐transform infrared spectroscopy (FT‐IR, Vertex 70, Bruker) and X‐ray photoelectron spectroscopy (XPS, K‐alpha, Thermo Scientific). The FT‐IR spectra were recorded in the range of 4000–650 cm^−1^ to investigate changes in chemical bonding and functional groups after ion treatment. The XPS analysis was conducted with an Al K‐α X‐ray source over a circular sampling area with a diameter of 400 µm. The crystallinity of the pristine PAH and ISC–PAHs was further analyzed using X‐ray diffraction (XRD, SmartLab, Rigaku, Japan) and Raman spectra were acquired using a confocal Raman microscope (LabRAM Aramis, Horiba Scientific, Japan) equipped with a 785 nm excitation laser. Spectra were collected in the range of 200–1800 cm^−1^. Liquid contact angles were measured using a contact angle measurement system equipped with a dynamic image capture camera (Smart Drop, FEMTOBIOMED, Korea) employing 5 µL droplets of deionized water. Fluorescence Recovery After Photobleaching (FRAP) analysis was conducted to evaluate the effective diffusion properties of the ISC–PAH using a confocal Laser Scanning Confocal Microscope (LSM 980, Carl Zeiss Germany) equipped with a 40x water‐immersion objective lens (NA = 1.2). Fluorescein Isothiocyanate (FITC)–dextran (4, 20, and 40 kDa) was dissolved in PBS at a concentration of 0.2 mg mL^−1^, and pristine PAH and ISC–PAH samples were immersed in the respective solutions for 24 h. The hydrogel samples were placed on a 35 mm Glass Bottom Microwell Dish and were analyzed at room temperature. A sequence of 500 frames was imaged with an image size of 512 × 512 pixels and a pixel size of 86 nm. FRAP bleach pulse was applied after 15 frames at a circular ROI. In the bleaching process, the 488 nm laser was set to 1% and 405 nm laser was set to 1% simultaneously and imaging was carried out using a laser intensity of 1%. Three parallel FRAP experiments were conducted with each hydrogel containing various sizes of FITC–dextran. The half‐recovery time (τ_1/2_) was determined from the fluorescence recovery curves as the time required to reach 50% of the plateau intensity, which represents the characteristic index of molecular diffusion rate within each hydrogel. The percent recovery, representing the mobile fraction of fluorescent molecules, was calculated from the intensity‐time profiles according to the following Equation (5)

(5) $$\begin{array}{c} Mobile\;Fraction\left(\%\right)=\frac{I_{\infty }-I_{0}}{I_{\mathrm{pre}}-I_{0}}\times 100\left(\%\right) \end{array}$$

where *I*_∞_ is the recovered fluorescence intensity at the plateau, *I*_0_ is the intensity immediately after bleaching, and *I*_pre_ is the pre‐bleach intensity. Moreover, the apparent diffusion coefficient (*D*_app_) was calculated using the Soumpasis analytical solution for 2D diffusion within a circular Region of Interest (ROI), as expressed by the following Equation (6)

(6) $$\begin{array}{c} D_{\mathrm{app}}=\frac{0.244\;\times \;\omega^{2}}{\tau_{1/2}} \end{array}$$

where ω is the radius of the bleached region and τ_1/2_ is the time required to reach half of the maximum fluorescence recovery. The calculated *D*_app_ was then normalized to the free diffusion coefficient (*D*_0_) of FITC‐dextran in PBS under identical temperature and ionic strength conditions, yielding a dimensionless *D*_app_/*D*_0_ ratio that reflects the degree of diffusion hindrance within each hydrogel.

### 4.4 Evaluation of Mechanical Properties and Swelling Properties of ISC–PAHs

The tensile and compressive properties of the ISC–PAHs were evaluated using a mechanical testing instrument (MultiTest 2.5‐DV, Mecmesin, UK) equipped with a 50 N load cell. Tensile tests were conducted at a speed of 50 mm min^−1^ using samples measuring approximately 15 × 20 × 2 mm^3^, while compressive tests were performed at a speed of 10 mm min^−1^ using cubic samples measuring 20 × 20 × 20 mm^3^. Rheological properties were assessed using a rheometer (MCR102, Anton Paar, Austria), where the ISC–PAH specimens were positioned between parallel plates with a 25 mm diameter and a 1 mm gap. Additionally, the viscosity of the ISC–PAHs was measured across a shear rate range of 0.01–100 s^−1^ to evaluate their flow behavior. Amplitude sweep tests were first conducted to determine the linear viscoelastic region (LVR), from which an appropriate strain amplitude was selected for each sample. Based on the identified LVR, frequency sweep tests were subsequently performed at a fixed strain specific to each sample over an angular frequency range of 0.1–100 rad s^−^^1^ to evaluate the storage modulus (*G*′), loss modulus (*G*″), and tan *δ*. The swelling behavior of the ISC–PAHs was determined by immersing pre‐weighed samples in either deionized (DI) water at 25°C, artificial sweat (a‐Sweat: 60 mM NaCl, 15 mM KCl, 20 mM lactate, 5 mM urea, 1 mM NH_4_Cl, 0.5 mM CaCl_2_), or artificial interstitial fluid (a‐ISF: 135 mM NaCl, 5 mM KCl, 1.5 mM CaCl_2_, 1 mM MgCl_2_, 2 mM lactate, 5 mM glucose) at 25°C and 37°C. At predetermined time points (0, 12, 24, and 48 h), samples were removed, gently blotted to remove excess surface water, and immediately weighed. The swelling ratio was calculated using the following Equation (7)

(7) $$\begin{array}{c} Swelling\;ratio\;\left(\%\right)=\frac{W_{t}-\;W_{i}}{W_{t}}\;\times \;100\;\left(\%\right)\; \end{array}$$

where *W*_t_ is the swollen weight at time *t*, and *W*_i_ is the initial weight of the hydrogel.

### 4.5 In Vitro Biocompatibility of ISC–PAHs

NIH3T3 fibroblast cells (KCLB No. 80 054; Korean Cell Line Bank, Seoul, Korea) (2.25 × 10^5^ cells mL^−1^) were cultured in a well plate with DMEM (Dulbecco's modified Eagle Medium) supplemented with 10% bovine calf serum and 1% penicillin‐streptomycin. ISC–PAHs were placed in the transwells and incubated with the fibroblast cells. Cell viability was assessed on days 1, 3, and 5 using a Live/Dead assay kit (L3224, Invitrogen, USA) following the manufacturer's instructions. A laser scanning confocal microscope (LSM 980, Carl Zeiss, Germany) was used to capture fluorescence images of live and dead cells. Cell viability was quantitatively evaluated using the Cell Counting Kit‐8 (CCK‐8, Dojindo, Japan; WST‐8 based), where the absorbance at 450 nm after 1 h incubation directly reflects the metabolic activity of living cells.

### 4.6 Electrochemical Characterization of Analyte Diffusion Across ISC–PAHs

All electrochemical characterizations were performed using a PalmSens4 potentiostat (PalmSens, Houten, Netherlands). The diffusion behavior of the analyte within ISC–PAHs was assessed by inserting an Au microelectrode (diameter: 12.5 µm) into various types of ISC–PAH cubes (2 × 2 × 2 cm^3^). These ISC–PAH cubes with Au microelectrode were immersed in a 5 mM K_3_[Fe(CN)_6_]/K_4_[Fe(CN)_6_] solution, with a Pt counter and an Ag/AgCl reference electrode placed in the same solution. Electrochemical linear scan voltammetry (LSV) was performed in a potential range of −0.3–0.7 V vs. Ag/AgCl at a scan rate of 0.025 V/s to characterize the diffusion properties of the redox probes across the ISC–PAHs matrix.

### 4.7 Preparation of Conductive‐Electrochemical Catalytic Hydrogel (C‐ECH) Precursor

The C‐ECH precursor solution was prepared through the following procedure. Ni‐doped Prussian Blue (Ni‐PB) nanoparticles with catalytic activity for electrochemical H_2_O_2_ reduction were synthesized by mixing a 5 wt% Prussian Blue dispersion, 0.1 M HCl/0.1 M KCl solution, 2.5 mM NiCl_2_, and 1.2 mM K_3_[Fe(CN)_6_] solution at various volume ratios. The suspension was stirred at room temperature for 1 h, then allowed to settle completely, and the supernatant was carefully removed. The precipitate was redispersed in fresh 0.1 M HCl/0.1 M KCl solution and thoroughly washed. It was then allowed to settle again, after which the supernatant was carefully removed. This washing process was repeated five times. Subsequently, the purified Ni‐PB nanoparticles were redispersed to a final volume of 1 mL in 0.1 M HCl/0.1 M KCl solution. Next, equal volumes of 20 wt% PVA solution and PEDOT:PSS dispersion were mixed at 300 rpm for 3 min. This mixture was then combined with an equal volume of the synthesized Ni‐PB nanoparticle suspension and gently homogenized until uniform dispersion was achieved.

### 4.8 Electrochemical Determination of Hydrogen Peroxide Using C‐ECH‐Modified SPCEs

To fabricate the C‐ECH‐modified Screen‐Printed Carbon Electrode (C‐ECH/SPCE), C‐ECH precursor solution was drop‐cast on the working electrode (diameter: 4 mm) of the SPCE, which was custom‐made on a Poly(ethylene terephthalate) (PET) substrate in the laboratory. The electrode was then dried at 60°C for 5 min. The electrocatalytic performance of the C‐ECH/SPCE for hydrogen peroxide (H_2_O_2_) reduction was evaluated by cyclic voltammetry (CV) and chronoamperometry (CA). CV measurements were initially performed in PBS to establish a baseline and assess the redox property of C‐ECH, while scanning in the potential range of ‐0.3–0.6 V (vs. Ag/AgCl) at a scan rate of 0.1 V s^−1^. CA measurements were performed at an applied potential of −0.1 V (vs. Ag/AgCl) for 60 s, assessing the current responses to varying H_2_O_2_ concentrations ranging from 0.1 to 1 mM with 0.1 mM increments.

### 4.9 Electrochemical Determination of Glucose Using ISC–PAHs‐Modified Glucose Biosensors

Various ISC–PAH‐modified enzymatic glucose biosensors were fabricated on SPCEs and evaluated for their biosensing performance. The SPCE was first modified following the C‐ECH/SPCE preparation procedure described above. Then, 4 µL of GOx (40 mg mL^−1^ in 1× PBS, pH 7.4) was drop‐cast onto the C‐ECH/SPCE and dried overnight at 4°C. Subsequently, ISC–PAHs were applied to the electrode surface. The biosensing performance of ISC–PAH‐modified glucose biosensors was evaluated by CA. Measurements were performed under the following glucose concentration ranges: For pristine‐PAH modified glucose biosensor, glucose concentrations ranged from 0.2 to 1.2 mM in 0.2 mM increments; for CaCl_2_–PAH‐modified glucose biosensors, concentrations ranged from 0.2 to 2.0 mM in 0.2 mM increments; for Na_2_SO_4_–PAH‐modified glucose biosensors, concentrations ranged from 0.1 to 1.0 mM in 0.1 mM increments; for Na_2_SO_4_/CaCl_2_–PAH‐modified glucose biosensors, concentrations ranged from 1 to 8 mM in 1 mM increments; for CaCl_2_/Na_2_SO_4_–PAH‐modified glucose biosensors, concentrations ranged from 2 to 30 mM in 2 mM increments. Each CA measurement was performed after a 3 min incubation period to allow sufficient enzymatic reaction of GOx, followed by measurement at an applied potential of −0.1 V (vs. Ag/AgCl) for 60 s.

### 4.10 Development of Highly Porous ISC–PAH (Na_2_SO_4_–PAH)‐Integrated Wearable Glucose Biosensor

To develop a wearable glucose biosensor, a custom‐designed three‐electrode system was fabricated via sequential stencil‐printing using functional inks specifically formulated for enhanced stretchability and electrical conductivity. An elastic graphite‐filled PEDOT:PSS (EG‐PEDOT:PSS) ink was developed and used as an electrode material. Eutectic gallium‐indium (EGaIn) ink was used as a conductive current collector, while iridium oxide (IrO*_x_*) was incorporated as the interface material for the reference electrode. To prepare the EG‐PEDOT:PSS ink, 3 mL of PEDOT:PSS suspension was mixed with 0.15 mL of dimethyl sulfoxide (DMSO) to improve conductivity and film formation. Then, 1 g of graphite powder was added to enhance electrochemical performance, followed by the addition of 1 mL of waterborne polyurethane (WPU, 40 wt% in deionized water) to provide mechanical elasticity and substrate adhesion. The resulting ink mixture was homogenized using a centrifugal mixer (ARE‐310, Thinky Corporation, Japan) for 20 min to achieve uniform dispersion and printability. Subsequently, the EGaIn ink was printed onto a SEBS substrate to form the current collector. The EG‐PEDOT:PSS ink was then stencil‐printed onto the EGaIn layer using AutoCAD‐designed stainless‐steel stencils (Sample PCB, Korea) to define the working, counter, and reference electrodes. Finally, IrO*_x_* was electrochemically deposited on the EG‐PEDOT:PSS electrode to fabricate the electrochemically stable reference electrode.

To fabricate the wearable glucose biosensors, C‐ECH, GOx, and Na_2_SO_4_–PAH were applied on the EG‐PEDOT:PSS working electrode. Using the resulting Na_2_SO_4_–PAH modified wearable glucose biosensors, the electrochemical properties and glucose biosensing performance were evaluated. CV measurement was conducted to characterize the electrochemical properties of the developed Na_2_SO_4_–PAH‐modified wearable glucose biosensors under a potential range of −0.3–0.7 V with a scan rate of 0.1 V/s (vs. Ag/AgCl). CA measurements were performed at −0.2 V (vs. IrO*_x_*), using glucose concentrations ranging from 0.1 to 1 mM in 0.1 mM increments. Moreover, to assess the selectivity of the biosensor, CA measurements were also conducted in 0.5 mM glucose solution with interferences found in human sweat: 50 µM uric acid, 10 µM ascorbic acid, and 5 mM lactate. Each CA measurement was conducted after a 3 min incubation to ensure sufficient enzymatic reaction of GOx.

### 4.11 Human Pilot Studies of Continuous Sweat Glucose Monitoring with Na_2_SO_4_–PAH‐Modified Wearable Glucose Biosensor in Healthy Participants

Continuous sweat glucose monitoring was performed with four healthy participants to evaluate the performance of the developed Na_2_SO_4_–PAH‐modified wearable glucose biosensor under practical wearable conditions. The wearable glucose biosensor was first connected directly to a Bluetooth potentiostat unit (Sensit Smart, PalmSens) for powering the biosensor and acquiring data. Subsequently, the wearable glucose biosensor was attached to the ventral forearm with an adhesive tape, which had been prepared with an alcohol swab to ensure low skin impedance and stable adhesion. After participants wore the developed wearable glucose biosensor under fasting conditions (≥12 h), they were instructed to begin cycling on a stationary ergometer to induce sweat secretion. Then, continuous sweat glucose monitoring was performed with the developed wearable glucose biosensor using the CA method at −0.2 V (vs. IrO*_x_*) for 60 s every 3 min over 100 min. Basal sweat glucose levels were quantified during the initial 20 min, after which participants were asked to drink a soft drink containing 54 g of sugar to elevate glucose levels. Blood glucose was simultaneously measured every 5 min using a commercial glucometer (Accu‐chek Instant, Roche Diabetes Care GmbH, Germany) to validate the physiological dynamic glucose trends. All study procedures were approved by the Institutional Review Board (IRB) at Gangnam Severance Hospital (IRB no: 3‐2024‐0124), and the study was conducted according to the Declaration of Helsinki, and all experimental procedures were performed with the informed consent of the participants. The glucose measurements conducted using sweat samples in this study were performed as a pilot investigation and are not classified as a clinical trial; no clinical trial registration number is available.

### 4.12 Development of Implantable Glucose Biosensor Using Less Porous ISC–PAH (CaCl_2_/Na_2_SO_4_–PAH) and Evaluation of Biosensing and Antifouling Performance

An implantable glucose biosensor was fabricated following the sequences described below. First, the Pt wire (diameter: 0.5 mm) used for the working electrode was subjected to two consecutive dip‐coating and drying cycles in the C‐ECH precursor solution (C–ECH/Pt). Subsequently, GOx solution (40 mg mL^−1^ in 1× Phosphate buffer saline, pH 7.4) was drop‐cast on the C‐ECH‐coated electrode and dried overnight at 4°C (GOx/C‐ECH/Pt). Next, GOx/C‐ECH/Pt underwent three consecutive dip‐coating cycles in the PAH precursor solution. The following procedure was the same as the CaCl_2_/Na_2_SO_4_‐modified glucose biosensor on SPCE mentioned above (CaCl_2_/Na_2_SO_4_–PAH/GOx/C‐ECH/Pt). CV was conducted to characterize the electrochemical properties of the developed implantable glucose biosensor with a Pt counter electrode and an Ag/AgCl reference electrode. The glucose biosensing performance of the CaCl_2_/Na_2_SO_4_–PAH modified implantable glucose biosensor was evaluated using CA recorded at an applied potential of −0.1 V (vs. Ag/AgCl) for 60 s after a 3 min incubation, using glucose concentrations ranging from 2 to 30 mM in 2 mM increments. Selectivity of the developed implantable glucose biosensors was assessed in the 5 mM of glucose presence of potential interferences (0.05 mM ascorbic acid, 0.05 mM uric acid, and 1 mM lactate) with CA at −0.1 V (vs. Ag/AgCl) for 60 s with 3 min incubation times, representative of glucose level in ISF, to account for typical interferences in ISF. Additionally, the mechanical durability and stability of the developed implantable glucose biosensor interfaces were evaluated by repeated penetration tests. For this, the phantom skin (freeze‐dried PVA hydrogel) was placed at the top of the electrochemical cell. The implantable glucose biosensing electrodes were subjected to multiple penetration cycles through phantom skin. Subsequently, glucose biosensing performance was evaluated using CA at −0.1 V (vs. Ag/AgCl) for 60 s, following a 3 min incubation in PBS containing 5 mg mL^−1^ bovine serum albumin (BSA) and 1 mM of glucose to confirm robustness against potential mechanical damage and biofouling.

As antifouling characteristics are a critical indicator of biosensor performance in implantable applications, a BSA adsorption test was conducted to further evaluate the biosensor's antifouling performance. Specimens (10 mm × 10 mm × 0.5 mm) were immersed in a 1 mg mL^−1^ BSA solution in PBS (50 mM, pH 7.0) and incubated at 4°C for 24 h. The remaining BSA concentration in the supernatant was measured by UV–visible spectrophotometry (*λ* = 368 nm, V‐650, JASCO, Japan). For fluorescence‐based evaluation, samples (10 mm × 10 mm × 0.5 mm) were immersed in 1 mg mL^−1^ FITC–BSA solution (PBS, pH 7.4) and incubated at 37°C for 1, 3, 5, 7, and 14 days. After each incubation period, the specimens were rinsed with PBS to remove unbound proteins and observed using a laser scanning confocal microscope (LSM 980, Carl Zeiss, Germany). Fluorescence intensity was quantified using ImageJ software to compare relative protein adsorption levels between coated and uncoated surfaces. Moreover, to electrochemically evaluate the antifouling performance, LSV was conducted using an Au microelectrode (D: 12.5 µm) embedded in CaCl_2_/Na_2_SO_4_–PAH cube (2 × 2 × 2 cm^3^) after incubation for 1, 3, 5, 7, and 14 days in 1 mg mL^−1^ BSA, followed by measurement in a 5 mM K_3_[Fe(CN)_6_]/K_4_[Fe(CN)_6_] dissolved in the same BSA‐containing PBS.

### 4.13 Continuous Interstitial Fluid Glucose Monitoring Using an Implantable Glucose Biosensor in Normal Mice

Normal male mice (20−25 g, 7 weeks) were purchased from Orient Bio Inc. (Gyeonggi‐do, South Korea), and they were then quarantined under semispecific pathogen‐free (semi‐SPF) conditions with a 12 h cycle of light/dark. To evaluate the biosensing performance of the developed implantable glucose biosensor, interstitial fluid glucose in mice was continuously monitored using the CaCl_2_/Na_2_SO_4_–PAH‐modified implantable glucose biosensors after a 12 h fasting state. To establish a three‐electrode system in the subcutaneous region, mice were anesthetized with isoflurane (1.5%−2% in O_2_, 1 L min^−1^). Three small dorsal incisions were made to implant a CaCl_2_/Na_2_SO_4_–PAH‐modified implantable glucose biosensor as the working electrode, a Pt wire electrode as the counter electrode, and an Ag/AgCl electrode as the reference electrode, enabling subsequent electrochemical measurements. ISF glucose was measured using the CA method at an applied potential of −0.1 V (vs. Ag/AgCl) for 60 s every 3 min over 120 min. Blood glucose was measured simultaneously every 5 min using a commercial glucometer (Accu‐chek Instant, Roche Diabetes Care GmbH, Germany) to validate the physiological dynamic glucose trends. Basal ISF glucose levels were quantified during the initial 20 min. Subsequently, a sterile 20% glucose solution (1 g kg^−1^) was injected intraperitoneally to elicit a glucose spike, followed by intraperitoneal administration of insulin (0.5 U kg^−1^) 35 min after glucose injection to lower the physiological glucose levels. All animal procedures were approved by the Yonsei University Institutional Animal Care and Use Committee (IACUC‐A‐2025‐05‐2050‐02).

### 4.14 Statistical Analysis

All experimental data were subjected to statistical analysis with a minimum sample size of three, using GraphPad Prism 8 software (GraphPad Software Inc.) and SPSS (IBM SPSS Statistics version 20, SPSS Inc.). Before analysis, the datasets were checked for normality and screened for potential outliers. No obvious outliers were detected, and all data were used without transformation. Results are presented as mean ± standard deviation (SD). An unpaired two‐sided *t*‐test, ordinary two‐way analysis of variance (ANOVA), Tukey's multiple‐comparisons test, Fisher's *r*‐to‐*z* transformation, and linear mixed‐effects models were performed to assess differences between groups, with individual variances calculated for each comparison. A significance level of *α* = 0.05 was applied. Statistical significance is indicated in the figures as follows: *p < 0.05, **p < 0.01, ***p < 0.001, and ****p < 0.0001, while “ns” denotes no significant difference.

## Author Contributions

D.L. and S.A.K. contributed equally to this work. D.L. and J.K. conceived the research. D.L. performed experiments, analyzed data, wrote the manuscript, and created figures. S.A.K. contributed to mechanical testing and data analysis, figure refinement, and manuscript revision. Y.L. assisted with mechanical testing. B.‐J.S., H.G.C., and S.B.H. fabricated and tested biosensors, with S.B.H. also supporting statistical analysis. T.Y.K. and S.J.K. conducted animal studies. K.S., K.Y.Y., and Y.L. contributed to material characterization and biological evaluation. S.P. supported the human pilot study and figure editing. J.K. and J.S. supervised the overall project and contributed to data interpretation and manuscript revision. J.K. and J.S. are corresponding authors. All authors discussed the results and approved the final manuscript.

## Conflicts of Interest

The authors declare no conflict of interest.

## Supporting information




**Supporting File**: adma72278‐sup‐0001‐SuppMat.docx.

## Data Availability

The data that support the findings of this study are available from the corresponding author upon reasonable request.
