# Supplementary material for: Ion‑Mediated Structural Engineering of Hydrogel Interfaces for Tunable Mechanical and Analyte Diffusion Properties in Electrochemical Biosensors
Source: Adv Mater. 2026 Mar 5;38(19):e15767. doi: 10.1002/adma.202515767 (PMC13040517; doi:10.1002/adma.202515767)
Supplement: Supplementary file 1 — Supporting File: adma72278‐sup‐0001‐SuppMat.docx. [file ADMA-38-e15767-s001.docx]

Supporting Information

**Ion‑mediated Structural Engineering of Hydrogel Interfaces for Tunable Mechanical and Analyte Diffusion Properties in Electrochemical Biosensors**

*Dongwook Lee, Soo A Kim, Beom-Jun Shim, Yurim Lee, Tae Young Kim, Sunghyun Park, Yeontaek Lee, Hyeong Gyu Choi, Kayoung Son, Su Bin Han, Keun Young Yook, Seo Jung Kim, Won-Yong Lee, Jungmok Seo*, Jayoung Kim**

**D. Lee, W.-Y. Lee**Department of Chemistry, Yonsei University, Seoul 03722, Republic of Korea
Center for Nanomedicine, Institute for Basic Science (IBS), Seoul 03722, Republic of Korea

**S. A. Kim, Y. Lee, T. Y. Kim, S. Park, K. Son, Y. Lee, K. Y. Yook, J. Seo**School of Electrical and Electronic Engineering, Yonsei University, Seoul 03722, Republic of Korea

**B.-J. Shim, S. B. Han**
Department of Medical Engineering, Yonsei University College of Medicine, Seoul 03722, Republic of Korea

**B.-J. Shim**
Graduate School of Medical Science, Brain Korea 21 Project, Yonsei University College of Medicine, Seoul 03722, Republic of Korea

**H. G. Choi, J. Kim**Department of Battery Engineering, Yonsei University, Seoul 03722, Republic of Korea

**S. J. Kim**Department of Pediatrics, Severance Children’s Hospital, Yonsei University, College of Medicine, Seoul 03722, Republic of Korea

**J. Kim**Department of Materials Science and Engineering, Yonsei University, Seoul 03722, Republic of Korea

**** Corresponding author.***

*Email: Jayoungkim@yonsei.ac.kr (J. Kim), Jungmok.seo@yonsei.ac.kr (J. Seo**)*


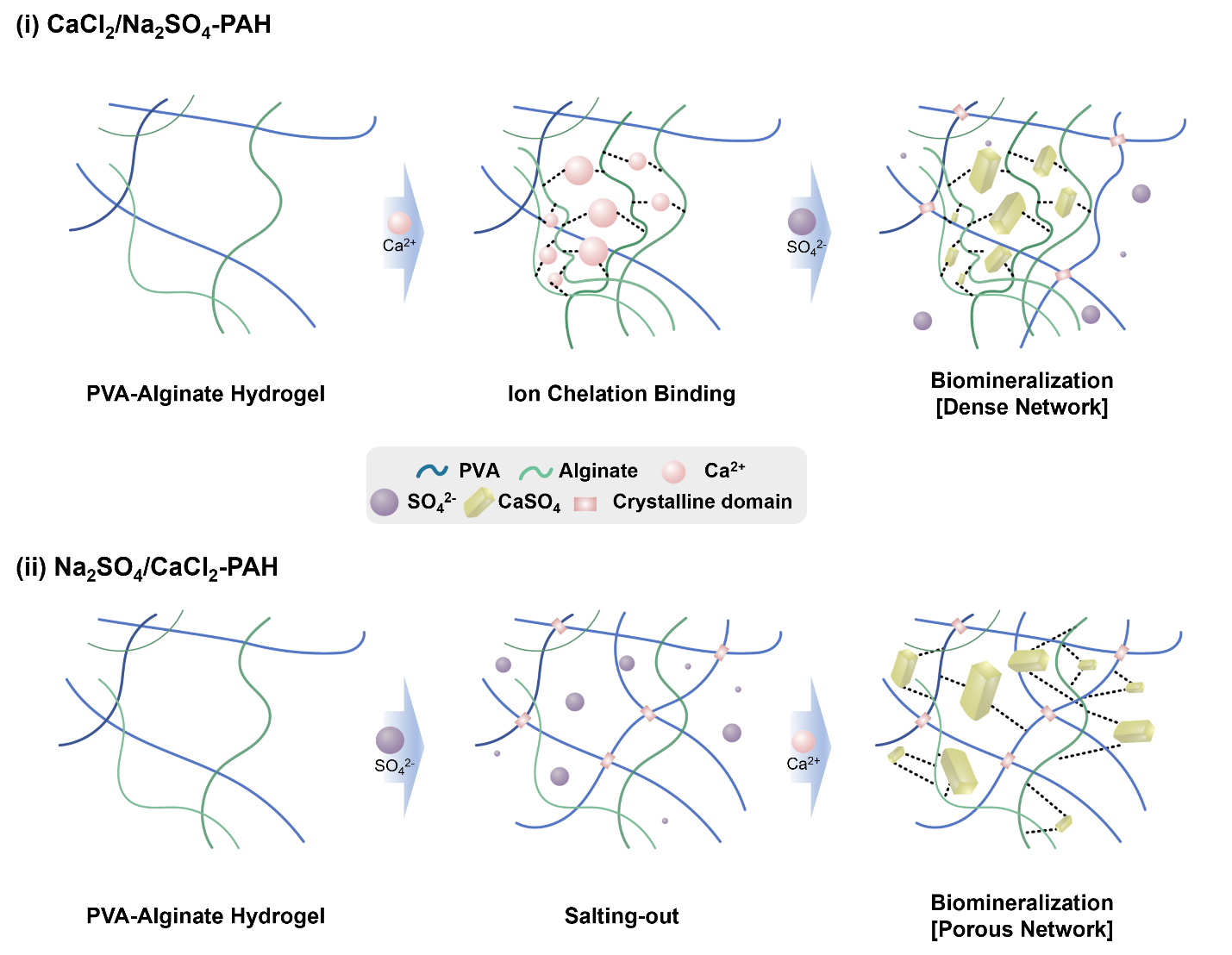


**Figure S1.** Schematic illustration of the structural evolution of PAH as a function of the ion-treatment sequence: (i) CaCl₂→Na₂SO₄ (CaCl_2_/Na_2_SO_4_–PAH) and (ii) Na₂SO₄→CaCl₂ (Na_2_SO_4_/CaCl_2_–PAH).


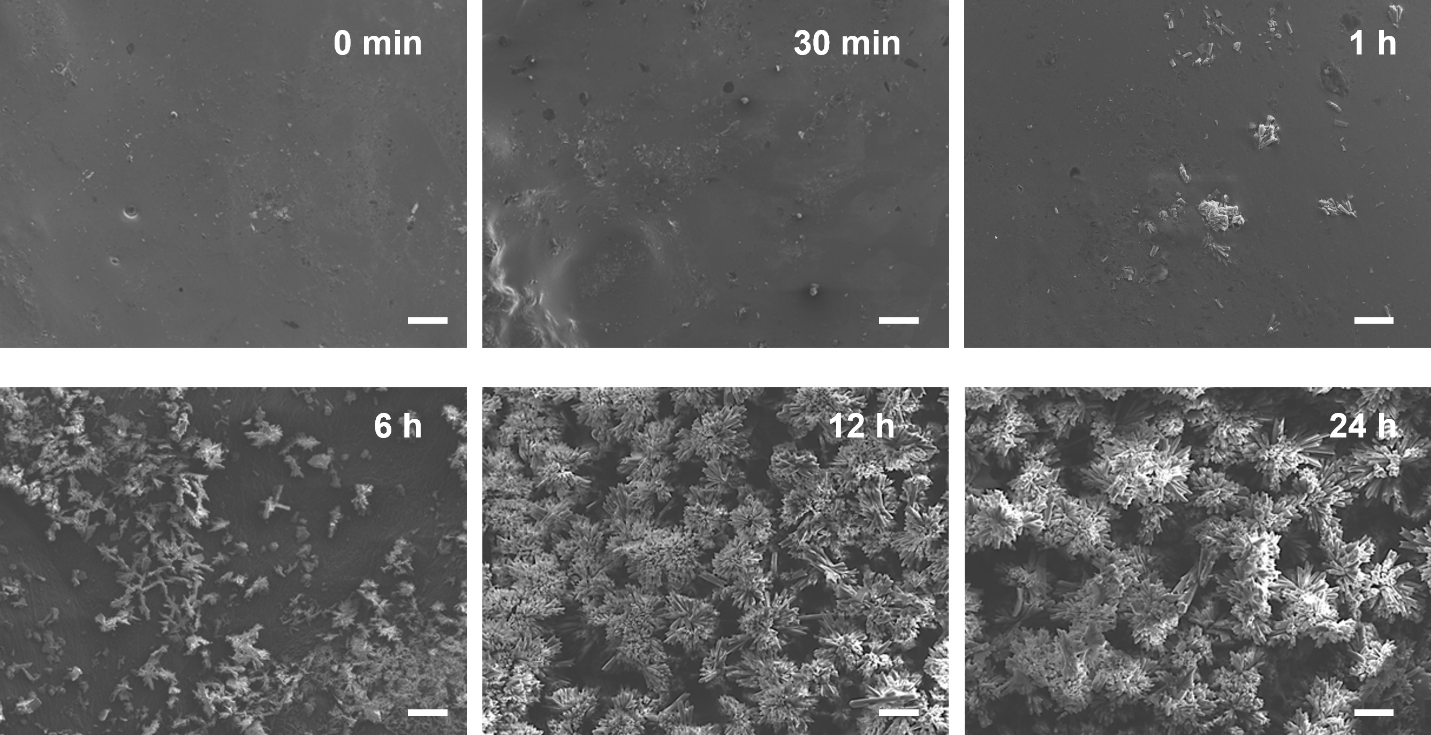


**Figure S2.** Time-resolved SEM images showing the evolution of CaSO_4_ crystals in a 2 M CaCl_2_-pretreated PAH upon treatment with 1.5 M Na_2_SO_4_ at various time points (*t* = 0, 30 min, 1 h, 6 h, 12 h, and 24 h). Scale bar = 10 μm.


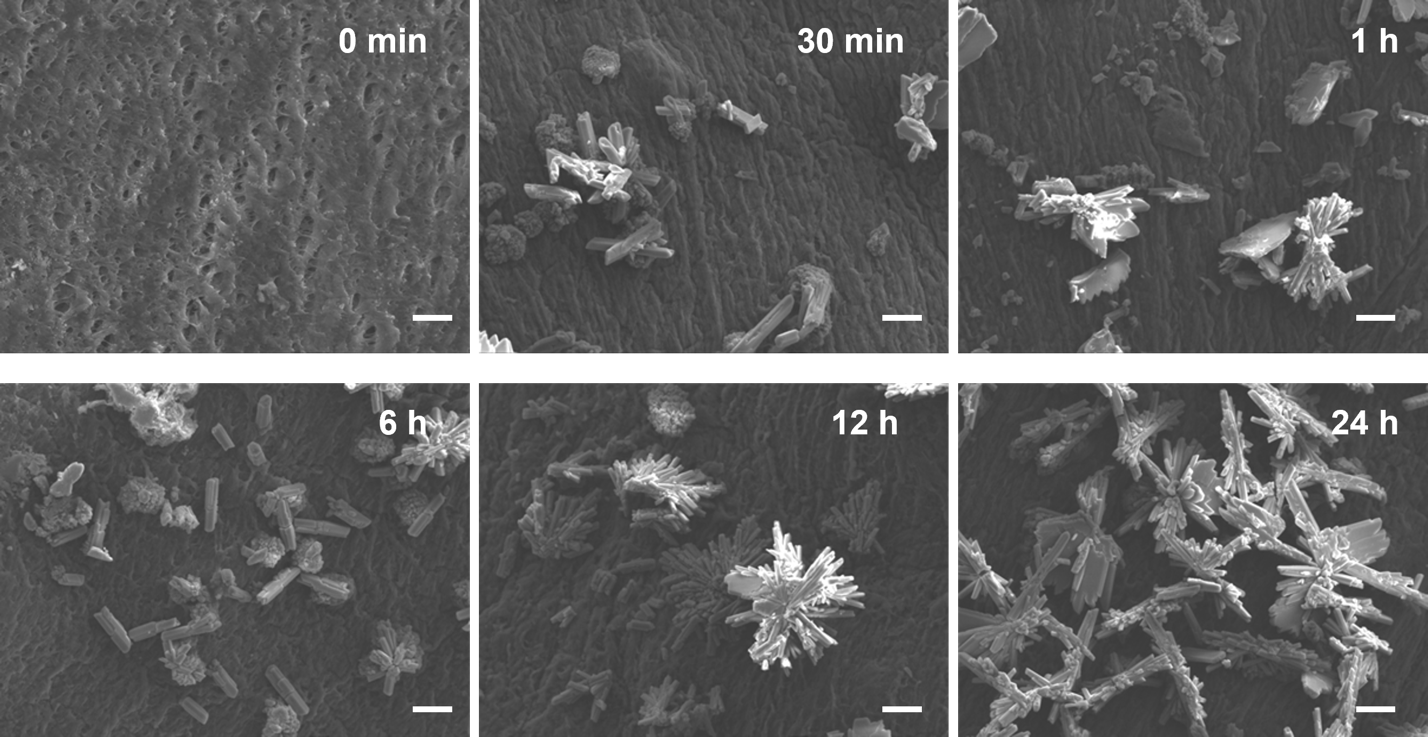


**Figure S3.** Time-resolved SEM images showing the evolution of CaSO_4_ crystals in a 1.5 M Na_2_SO_4_-pretreated PAH upon treatment with 2 M CaCl_2_ at various time points (*t* = 0, 30 min, 1 h, 6 h, 12 h, and 24 h). Scale bar = 10 μm.


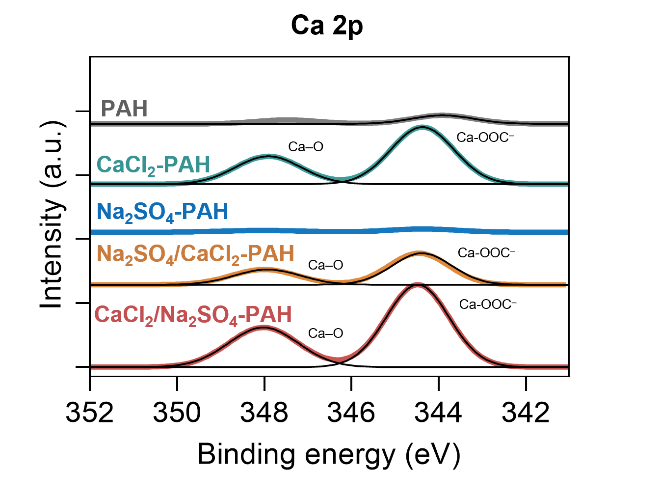


**Figure S4.** Ca 2p X-ray photoelectron spectra (XPS) of pristine PAH and ISC–PAHs.


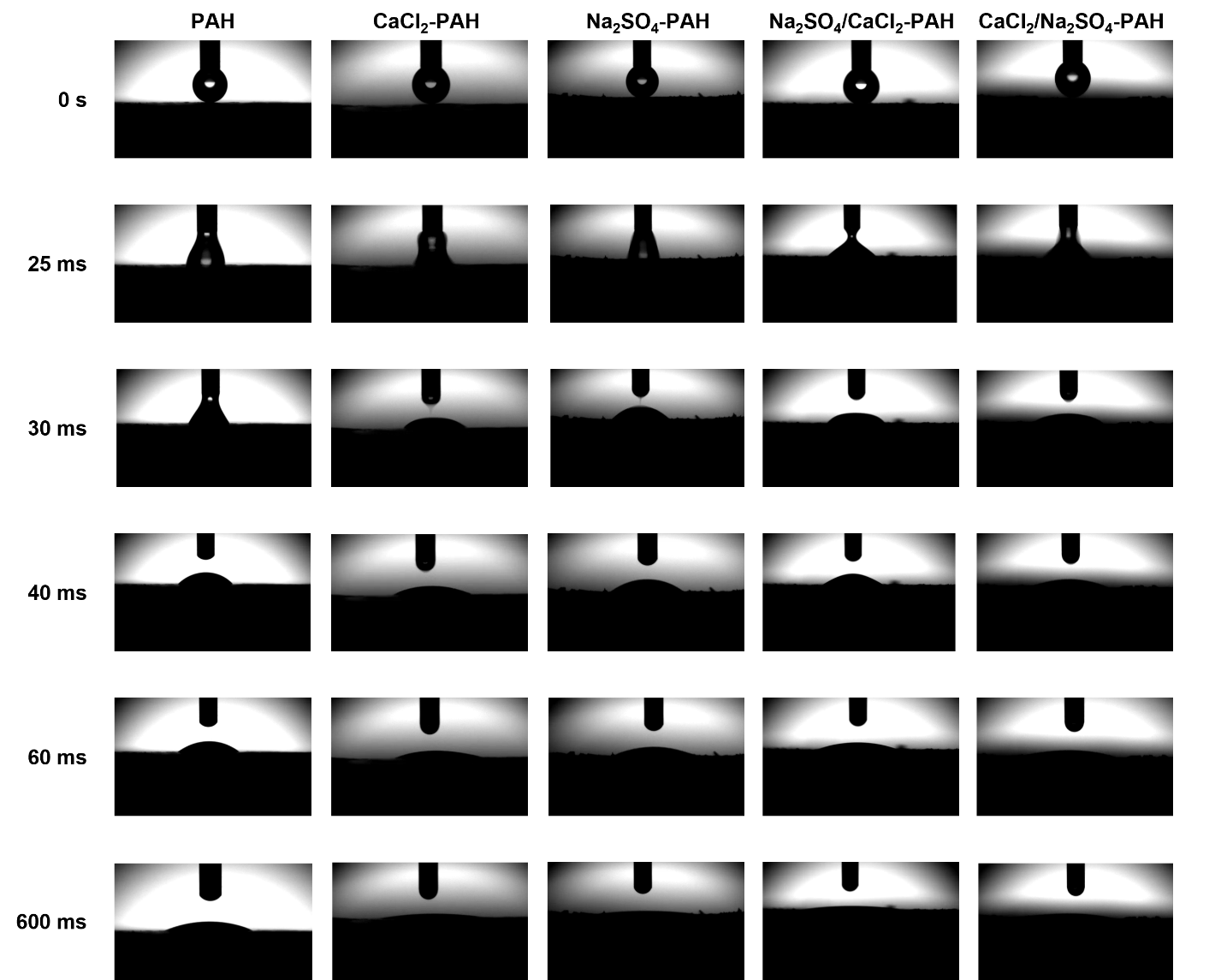


**Figure S5.** Time-dependent contact angle images of pristine PAH and ISC–PAHs at 0, 25, 30, 40, 60, and 600 ms after droplet contact.

**
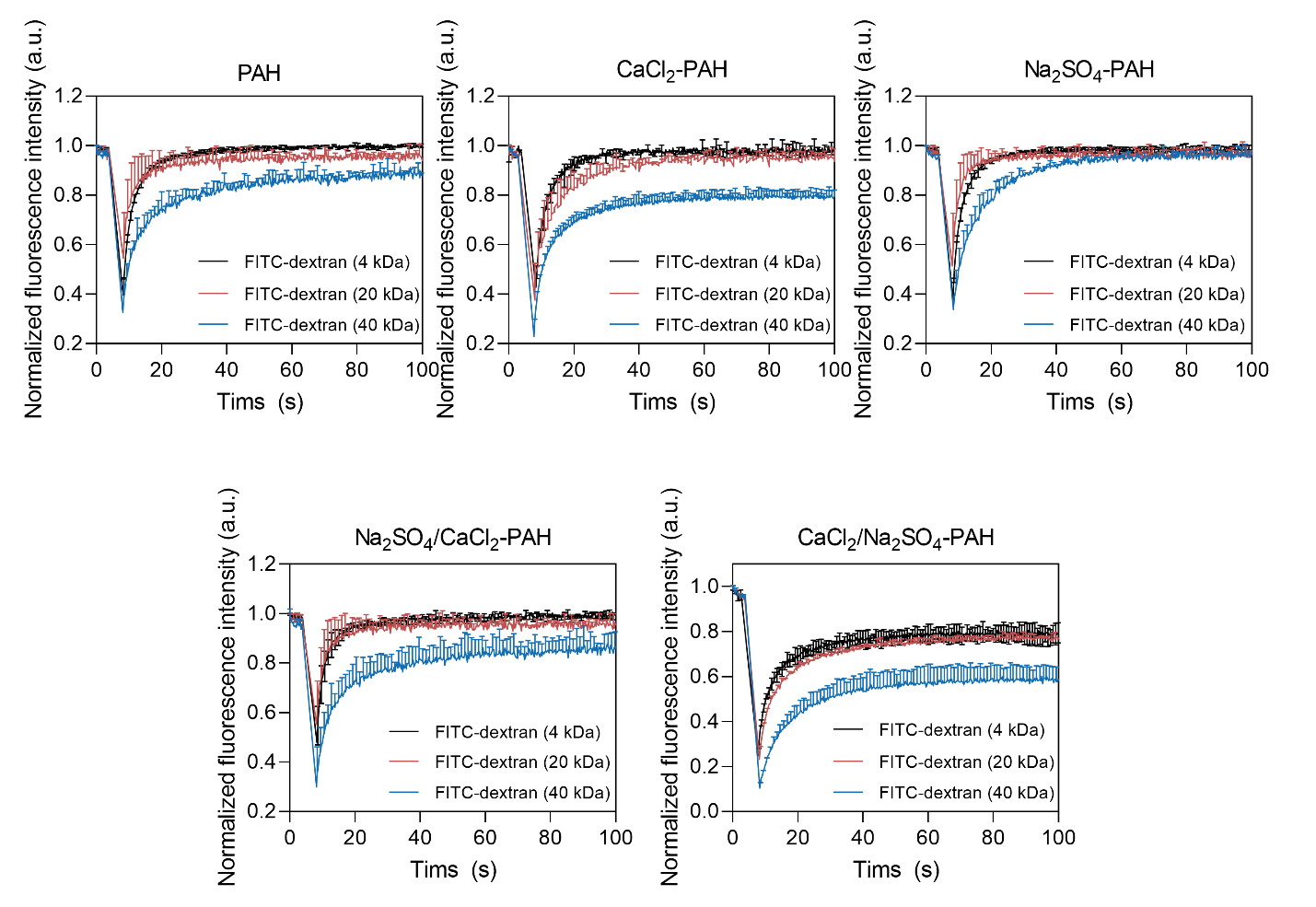
Figure S6.** Confocal fluorescence recovery after photobleaching (FRAP) curves of pristine PAH and various ISC–PAH hydrogels obtained using FITC–dextran probes with various molecular weights (4 kDa, 20 kDa, and 40 kDa). Data are presented as mean ± SD, *n* = 3.


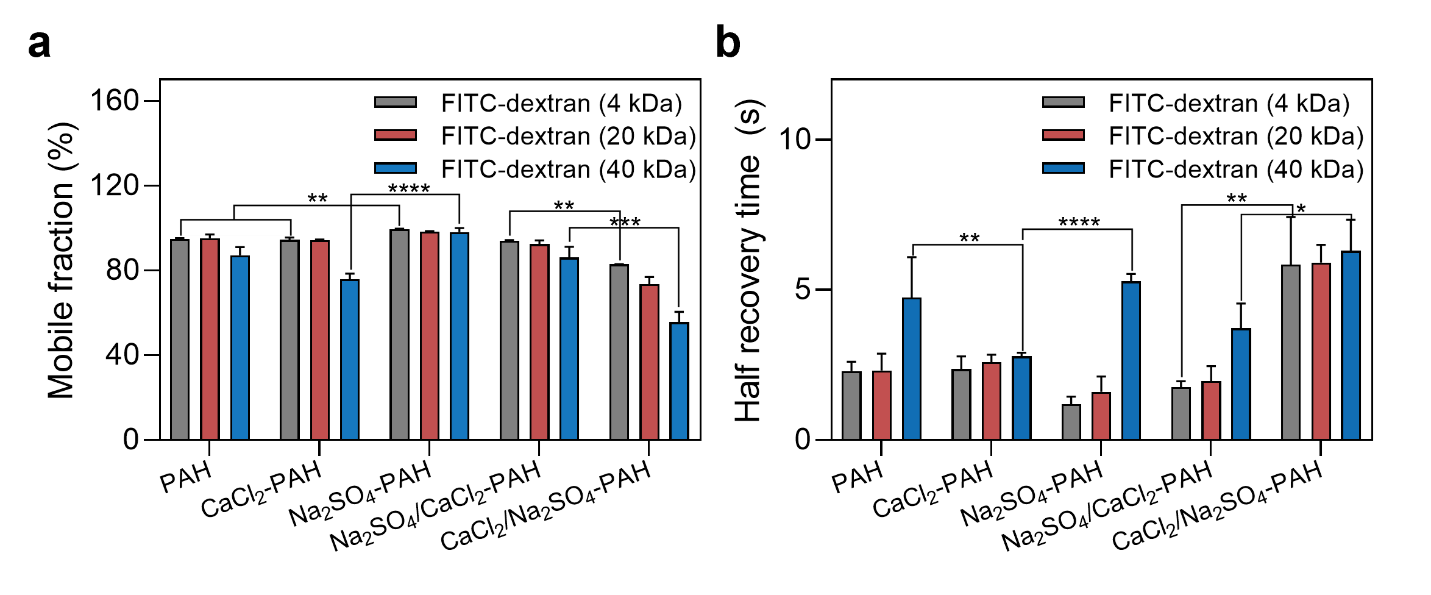


**Figure S7.** Quantitative analysis of a) mobile fraction and b) half-recovery time extracted from FRAP fluorescence recovery curves of pristine PAH and various ISC–PAH hydrogels obtained using FITC–dextran probes of different molecular weights (4 kDa, 20 kDa, and 40 kDa). Data are presented as mean ± SD, *n* = 3. (**p* < 0.05, ***p* < 0.01, ****p* < 0.001, *****p* < 0.0001; ns, not significant).


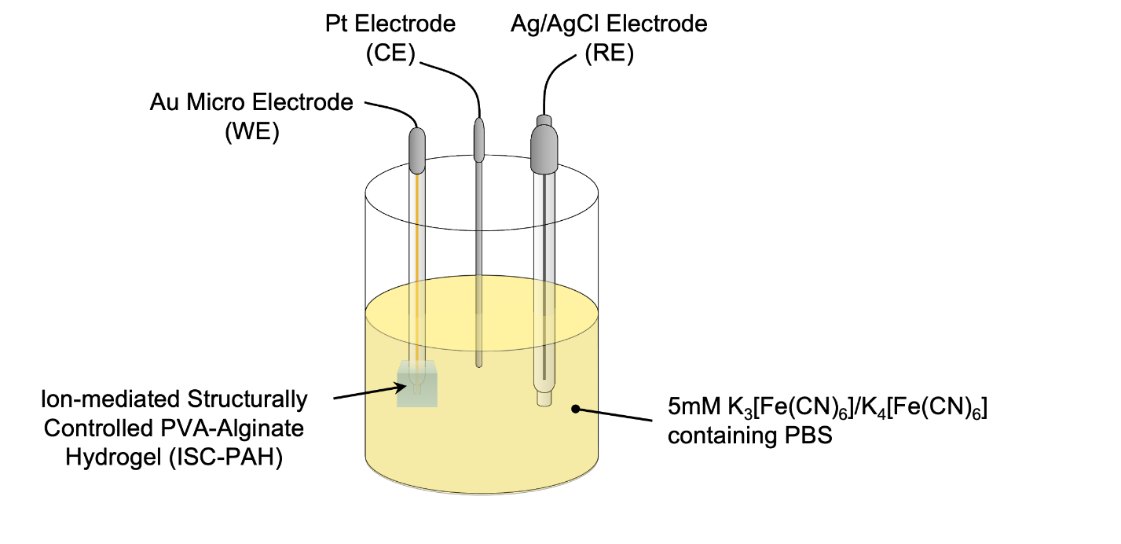


**Figure S8.** Schematic illustration of electrochemical characterization of the diffusion behavior of a redox probe across an ion-mediated, structurally controlled poly(vinyl alcohol)–alginate hydrogel (ISC–PAH) cube using an Au microelectrode (diameter: 12.5 μm). WE: working electrode; CE: counter electrode; RE: reference electrode.


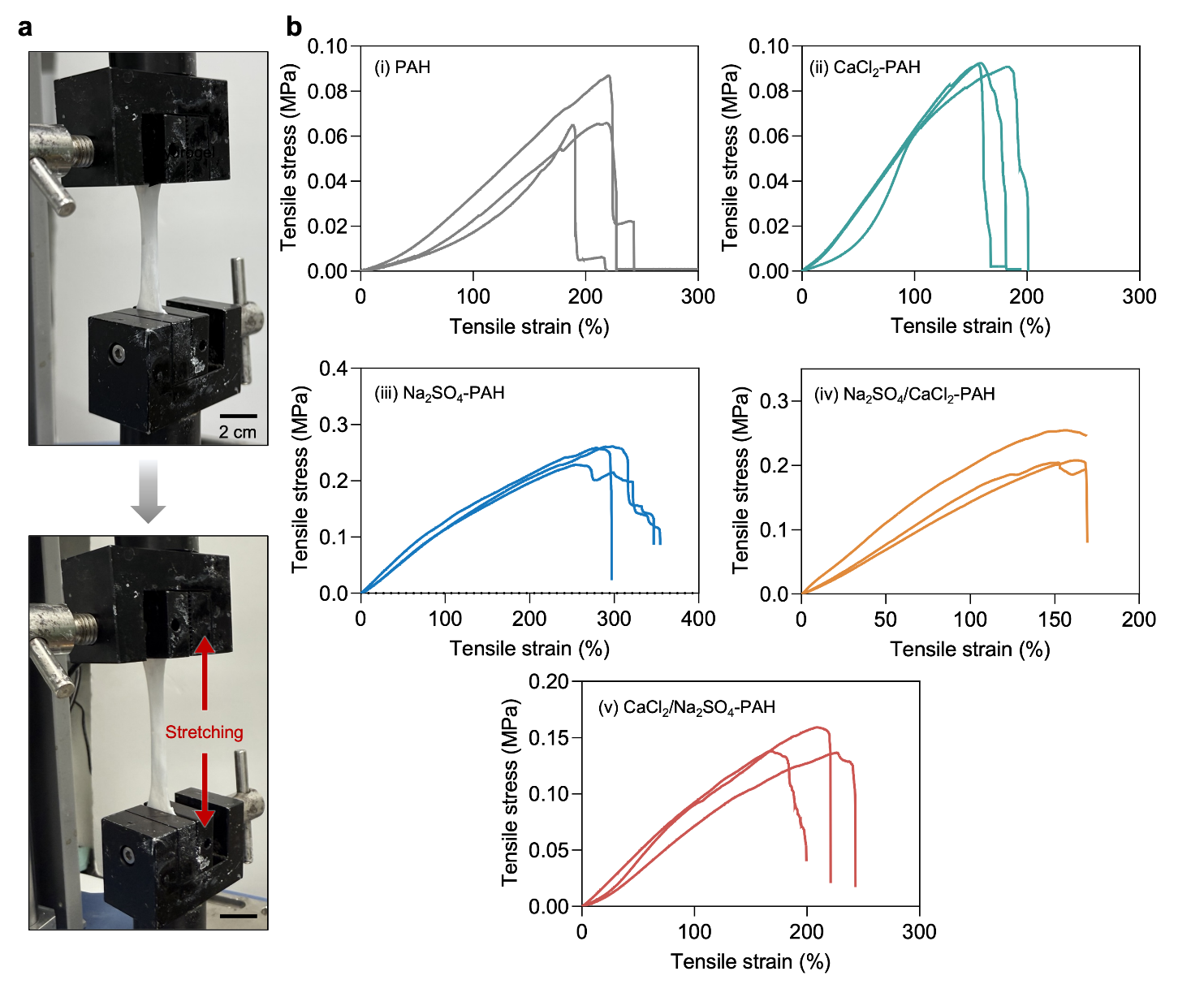


**Figure S9.** a) Photographs of pristine PAH and ISC–PAH samples during tensile testing, and b) tensile stress-strain curves obtained from uniaxial tensile test: (i) pristine PAH, (ii) CaCl_2_–PAH, (iii) Na_2_SO_4_–PAH, (iv) Na_2_SO_4_/CaCl_2_–PAH, and (v) CaCl_2_/Na_2_SO_4_–PAH.


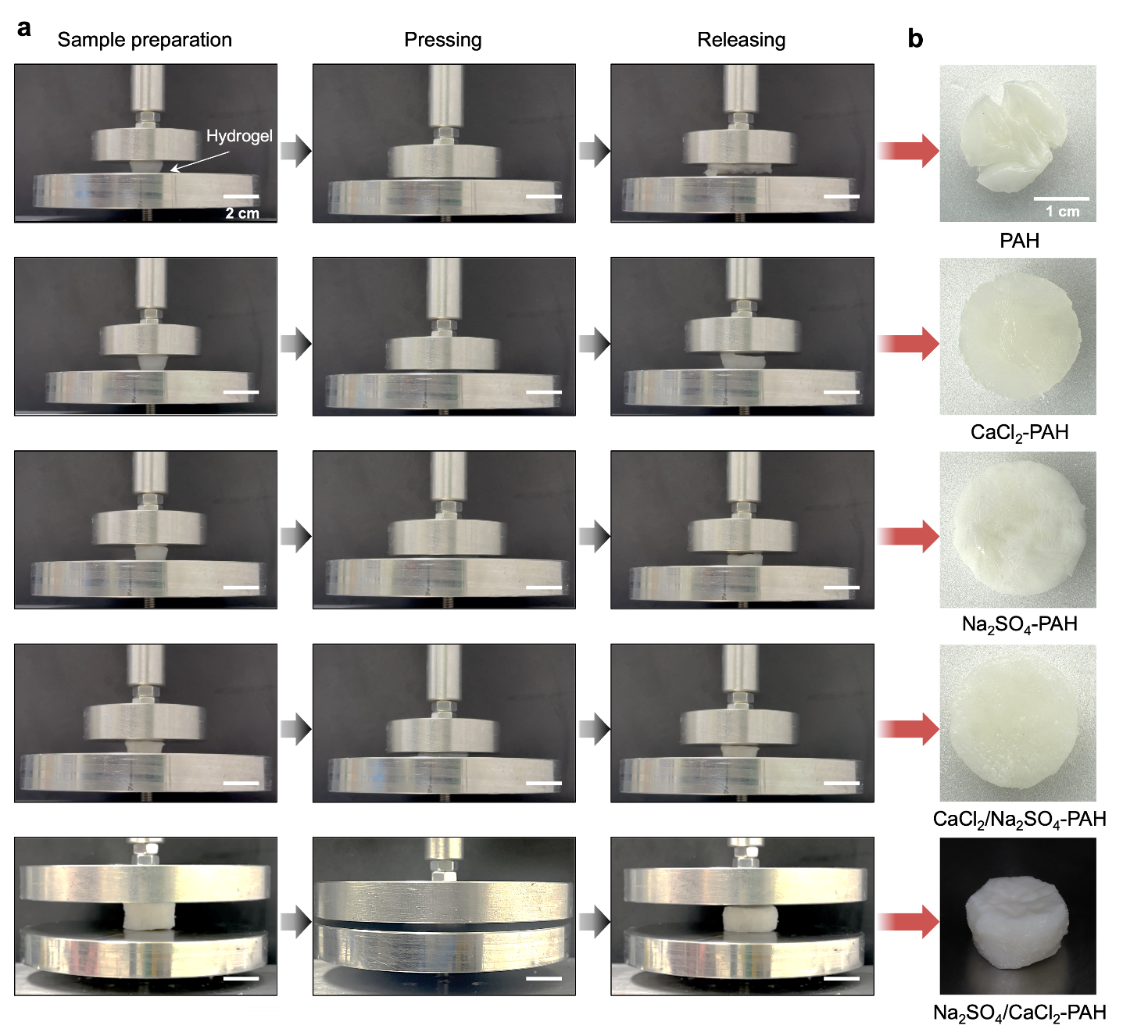


**Figure S10.** a) Sequential photographs of pristine PAH and ISC–PAH samples during compressive testing, showing the sample preparation, pressing, and release stages. b) Top-view images immediately after the release stage described in a). Sample groups include PAH, CaCl_2_–PAH, Na_2_SO_4_–PAH, CaCl_2_/Na_2_SO_4_–PAH, and Na_2_SO_4_/CaCl_2_–PAH.


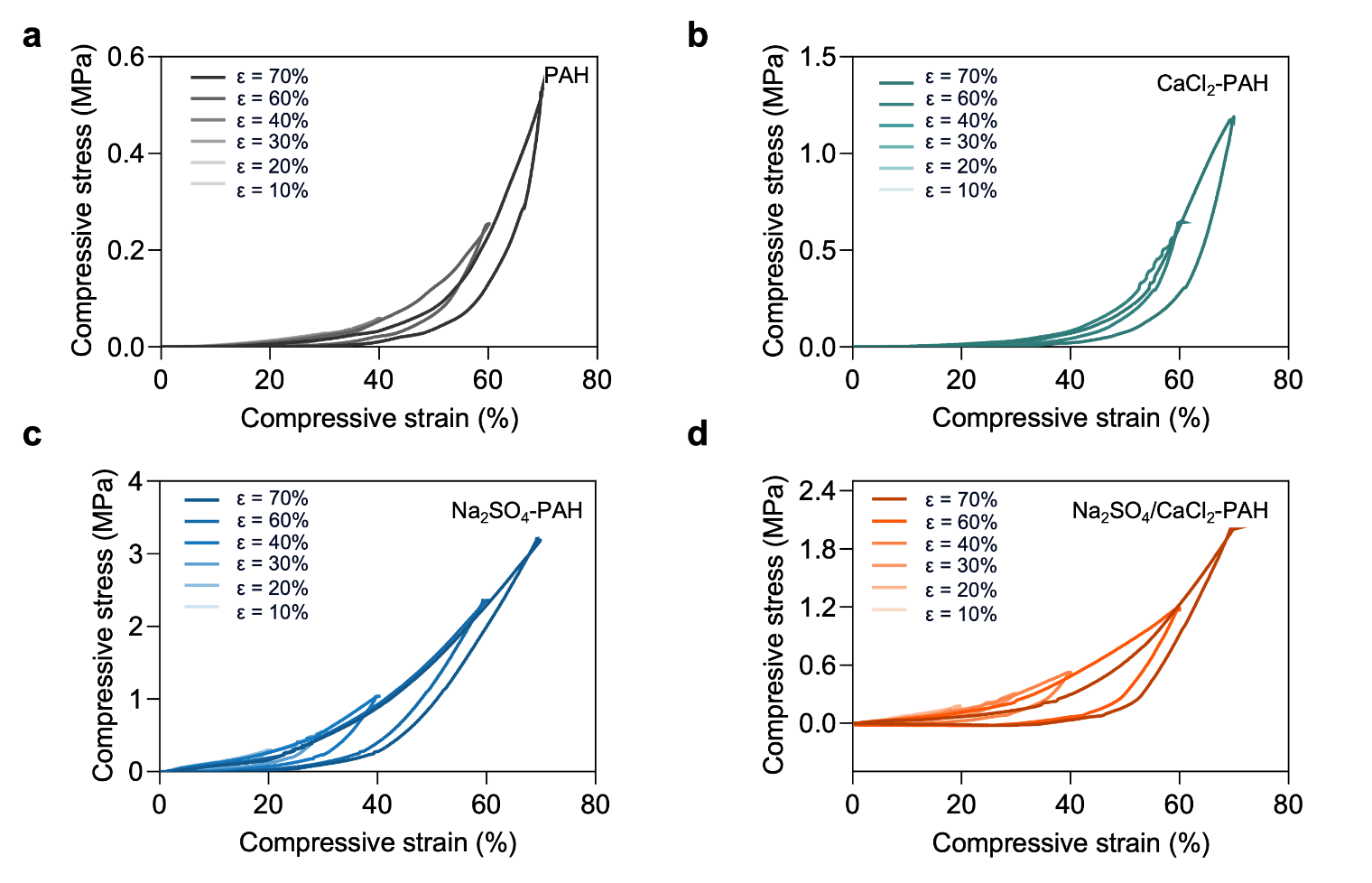


**Figure S11.** Cyclic compressive stress-strain curves of pristine PAH and ISC–PAH samples at various target strains (*ε* = 10–70%): a) pristine PAH, b) CaCl_2_–PAH, c) Na_2_SO_4_–PAH, and d) Na_2_SO_4_/CaCl_2_–PAH.


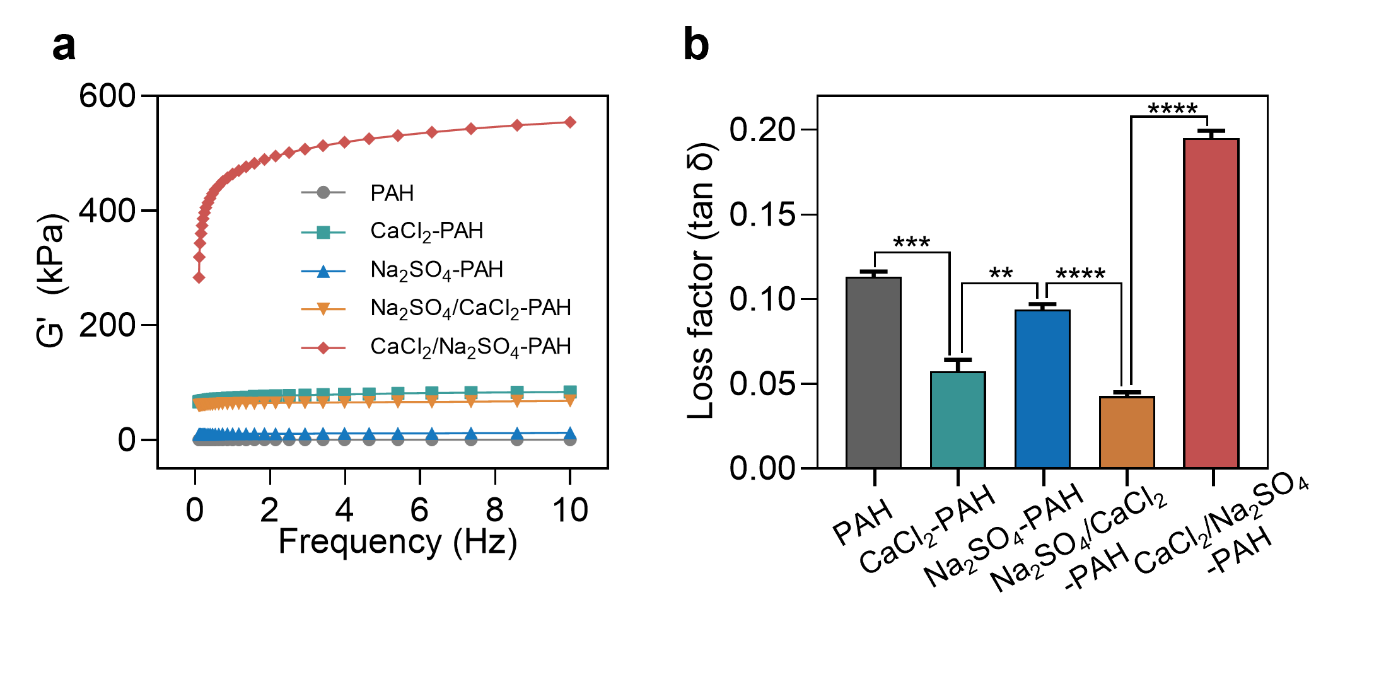


**Figure S12.** a) Storage modulus (*G′*) as a function of frequency and b) loss factor (tan *δ*, defined as *Gʺ*/*Gʹ*) for ISC–PAH samples. The loss factor is reported at 1 Hz, obtained from rheometric frequency-sweep measurements. Data are presented as mean ± SD, *n* = 3. (**p* < 0.05, ***p* < 0.01, ****p* < 0.001, *****p* < 0.0001; ns, not significant).


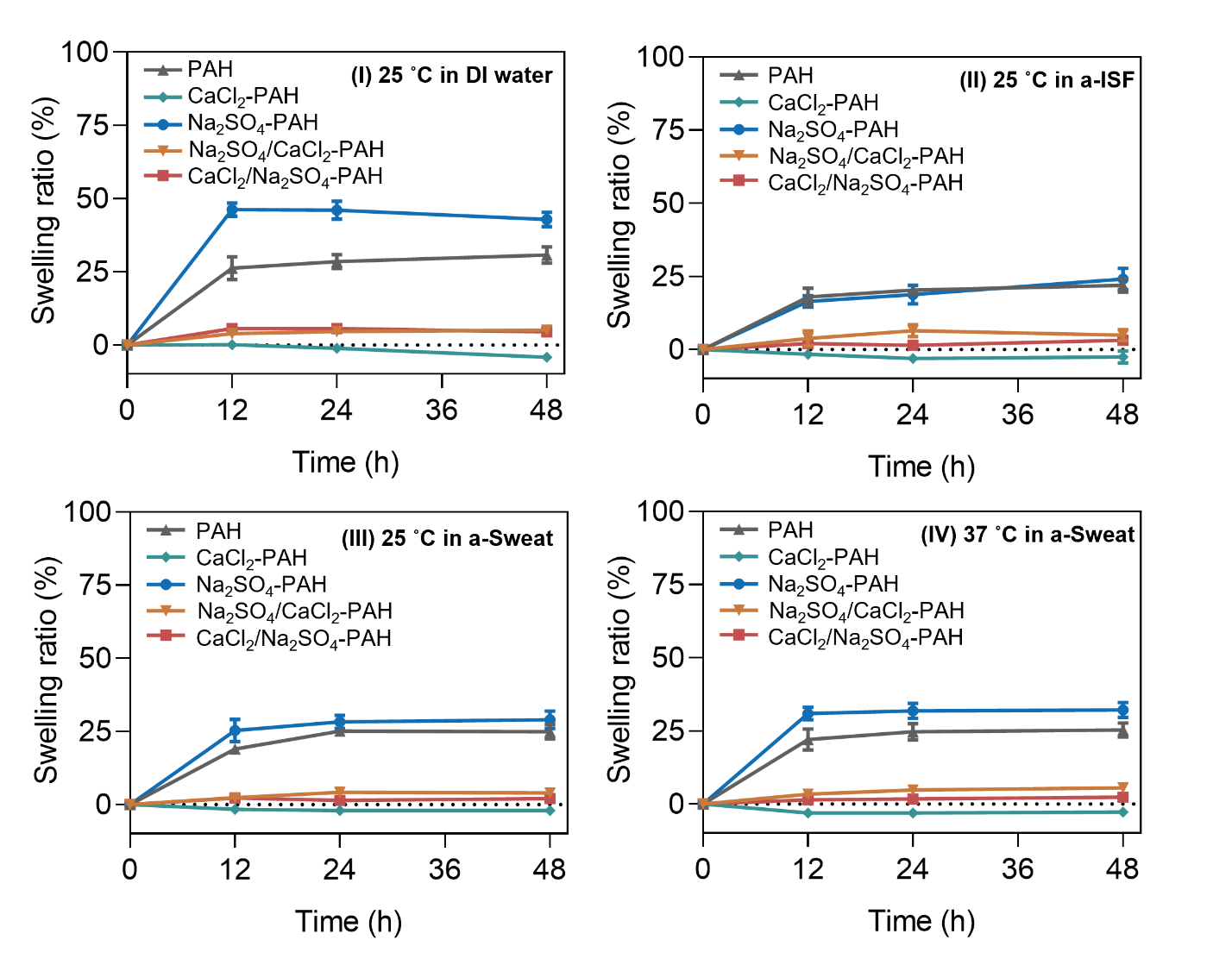


**Figure S13.** Swelling ratio of ISC–PAHs in (I) deionized water (DI water), (II) artificial interstitial fluid (a-ISF), and (III) artificial sweat (a-Sweat) over time at 25 °C and (IV) at 37 °C. Data are presented as mean ± SD, *n* = 3.

**
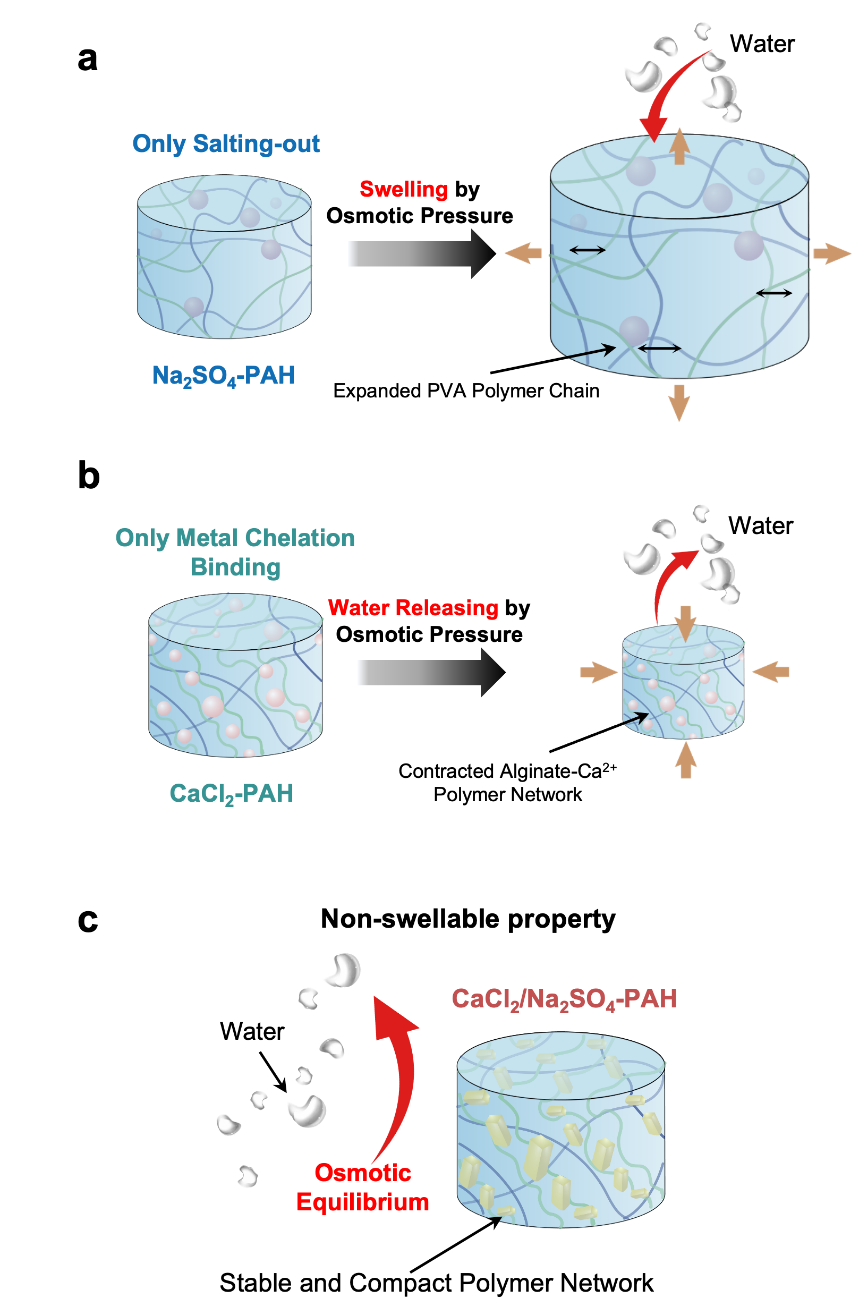
**

**Figure S14.** Schematic illustration of osmotic pressure-driven swelling behavior in ISC–PAHs. a) Na_2_SO_4_–PAH, b) CaCl_2_–PAH, and c) CaCl_2_/Na_2_SO_4_–PAH.


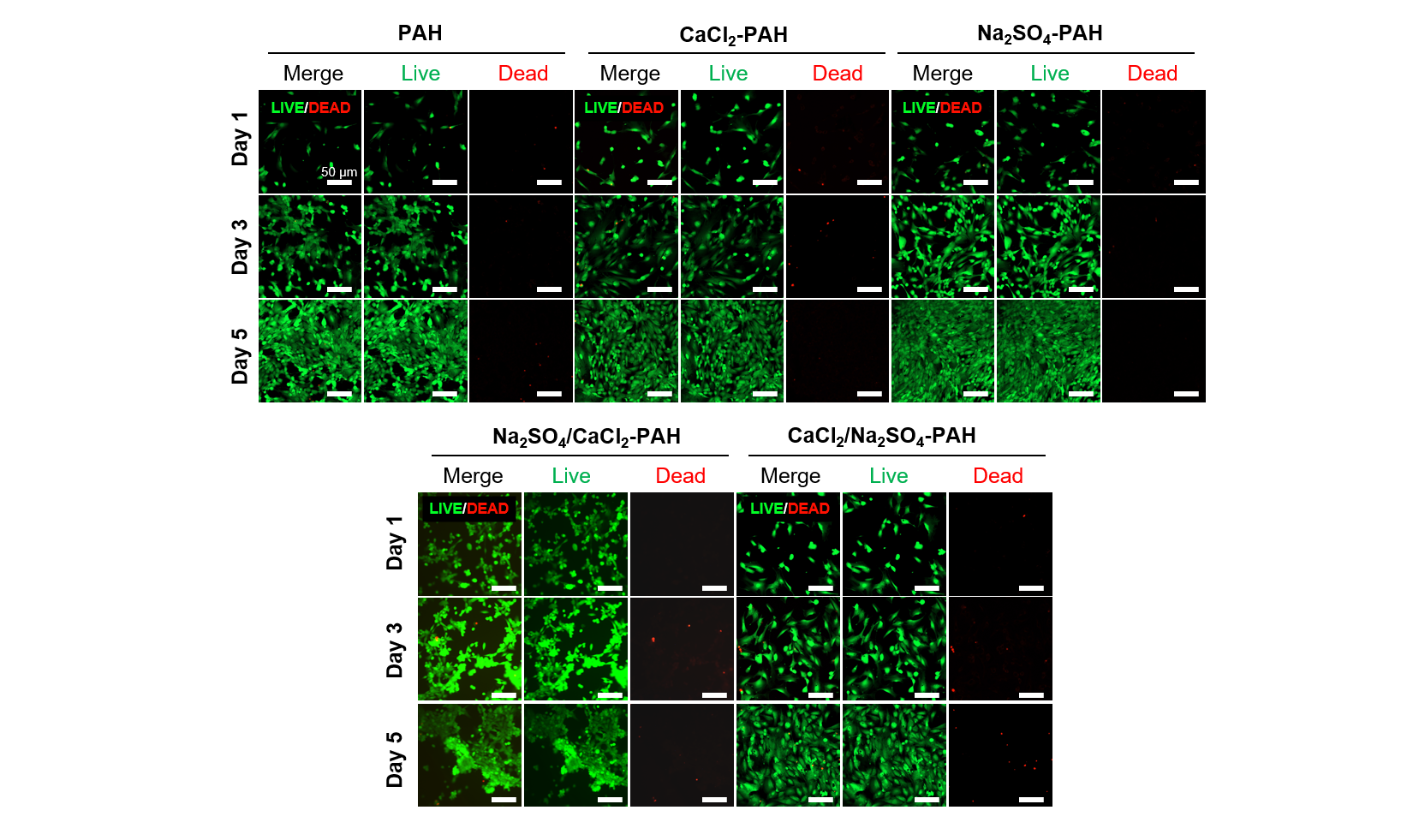


**Figure S15.** Live/dead fluorescence staining images of NIH-3T3 cells cultured for 1, 3, and 5 days on pristine PAH, CaCl_2_–PAH, Na_2_SO_4_–PAH, Na_2_SO_4_/CaCl_2_–PAH, and CaCl_2_/Na_2_SO_4_–PAH (live cells are stained green and dead cells are stained red). Scale bar = 50 μm.


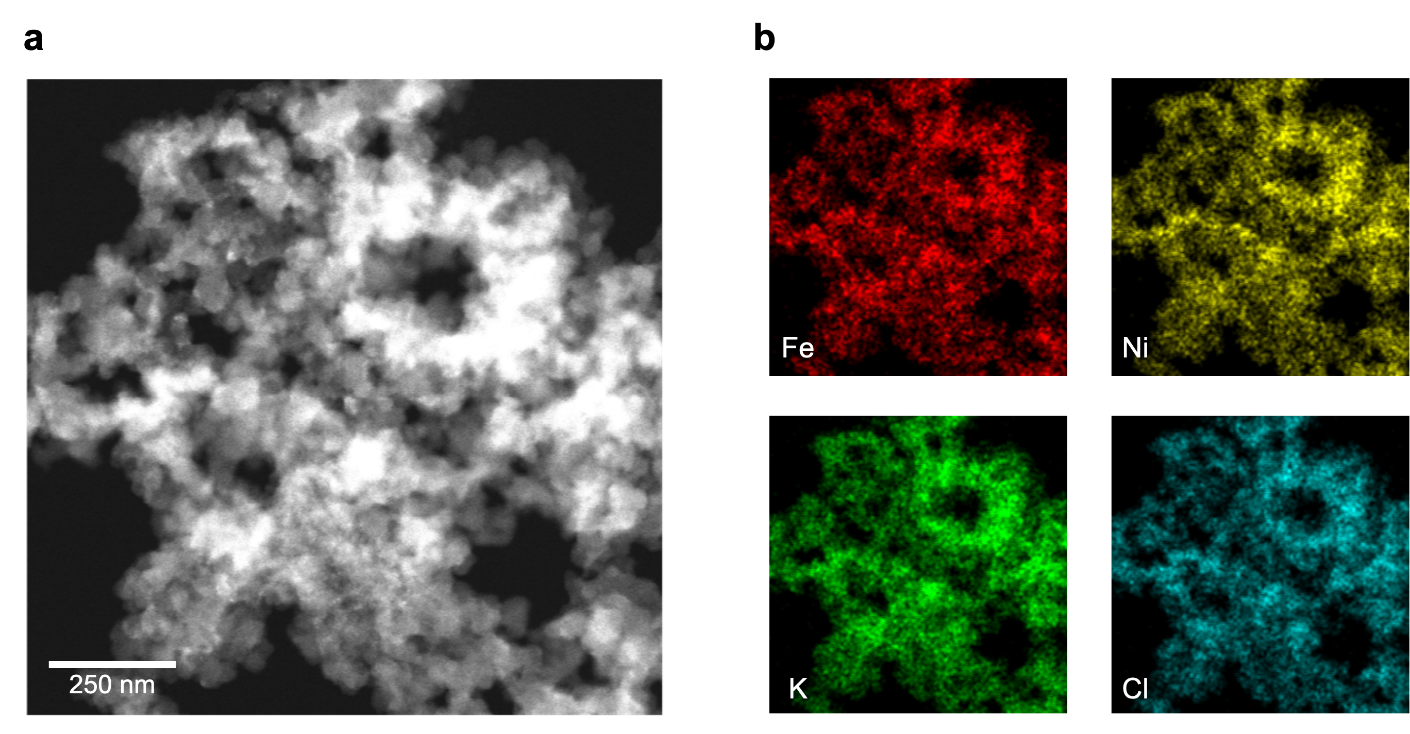


**Figure S16.** a) Transmission electron microscopy (TEM) image and b) corresponding energy-dispersive X-ray spectroscopy (EDS) mapping of nickel-doped Prussian Blue (Ni-PB) nanoparticles (*v*/*v* = 1:400). The 1:400 volume ratio denotes the proportion of Prussian Blue (PB) dispersed in the nickel precursor solution used during synthesis.

The electrochemical oxidation of hydrogen peroxide (H_2_O_2_), a byproduct generated during the enzymatic reaction of glucose oxidase (GO_x_), typically requires a high overpotential and exhibits poor selectivity due to the concurrent oxidation of interfering substances such as ascorbic acid and uric acid. To address this limitation, nickel-doped Prussian Blue (Ni-PB) nanoparticles were employed as the catalytic component of the C-ECH due to their exceptional catalytic activity toward the electrochemical reduction of H_2_O_2_, characterized by low overpotential and high operational stability. Conventional PB is known to experience lattice collapse caused by Fe-atom leaching under conditions exceeding pH 6.5.^[1,2]^ To mitigate this structural instability, nickel doping was introduced into the PB nanoparticles. Transmission electron microscopy (TEM) images confirmed the uniform doping of nickel within the PB nanoparticles.


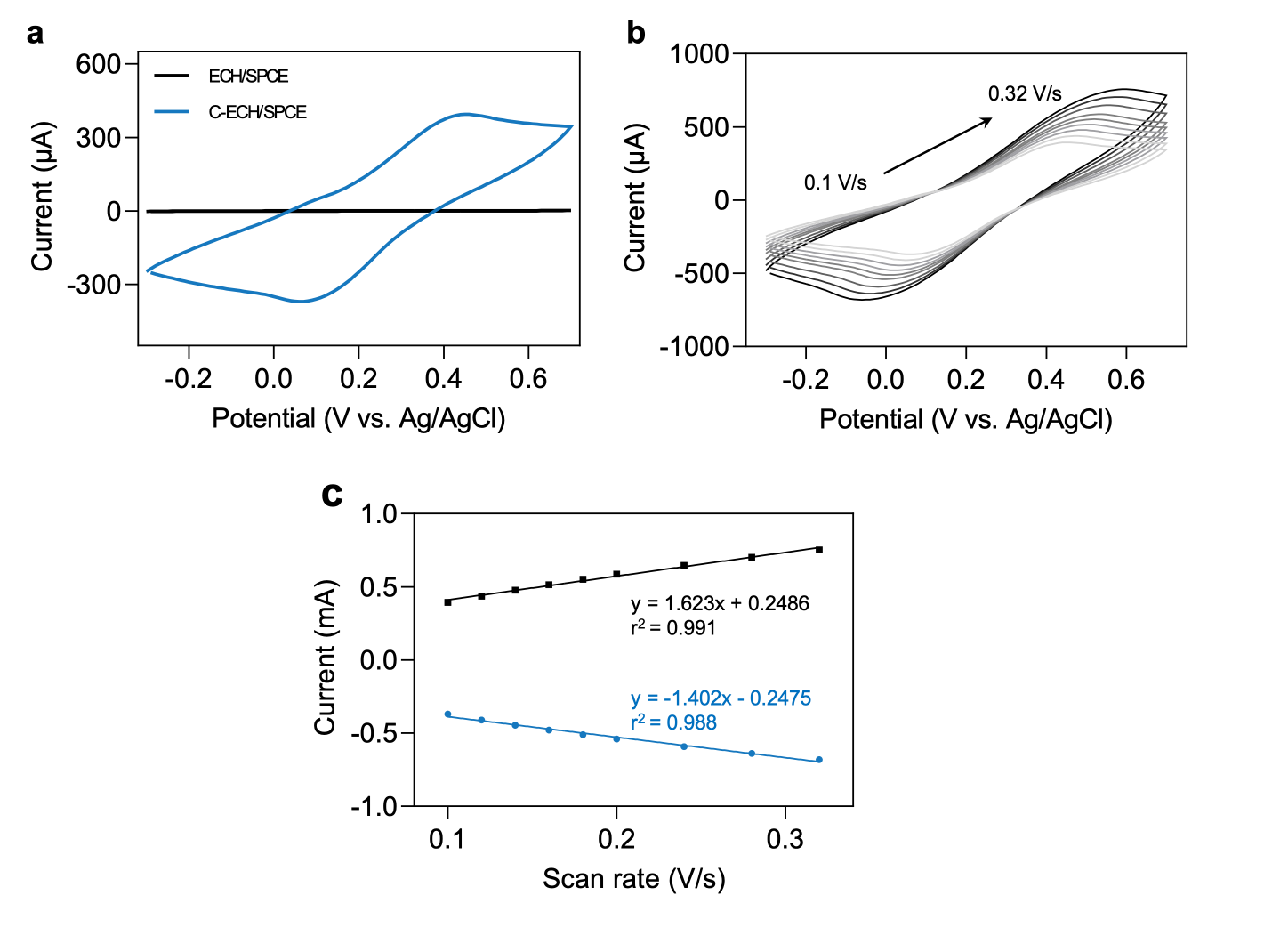


**Figure S17**. Electrochemical characterization of C-ECH/SPCE. a) Cyclic voltammograms of ECH/SPCE and C-ECH/SPCE recorded in the potential range from –0.3 to 0.7 V (vs. Ag/AgCl) at a scan rate of 0.1 V/s. b) Cyclic voltammograms of C-ECH/SPCE recorded at a scan rate ranging from 0.1 to 0.32 V/s in PBS. c) Corresponding plots of anodic and cathodic peak currents as a function of scan rate obtained from Cyclic voltammetry (CV) measurements.

Compared with an electrochemical catalytic hydrogel (ECH) composed of PVA and Ni-PB (without PEDOT:PSS), the C-ECH exhibited significantly enhanced redox peaks associated with Ni-PB. The peak current was linearly proportional to the scan rate, confirming a surface-confined redox reaction. This improvement can be attributed to the PEDOT:PSS, which effectively enhances the electron transfer pathway in C-ECH.


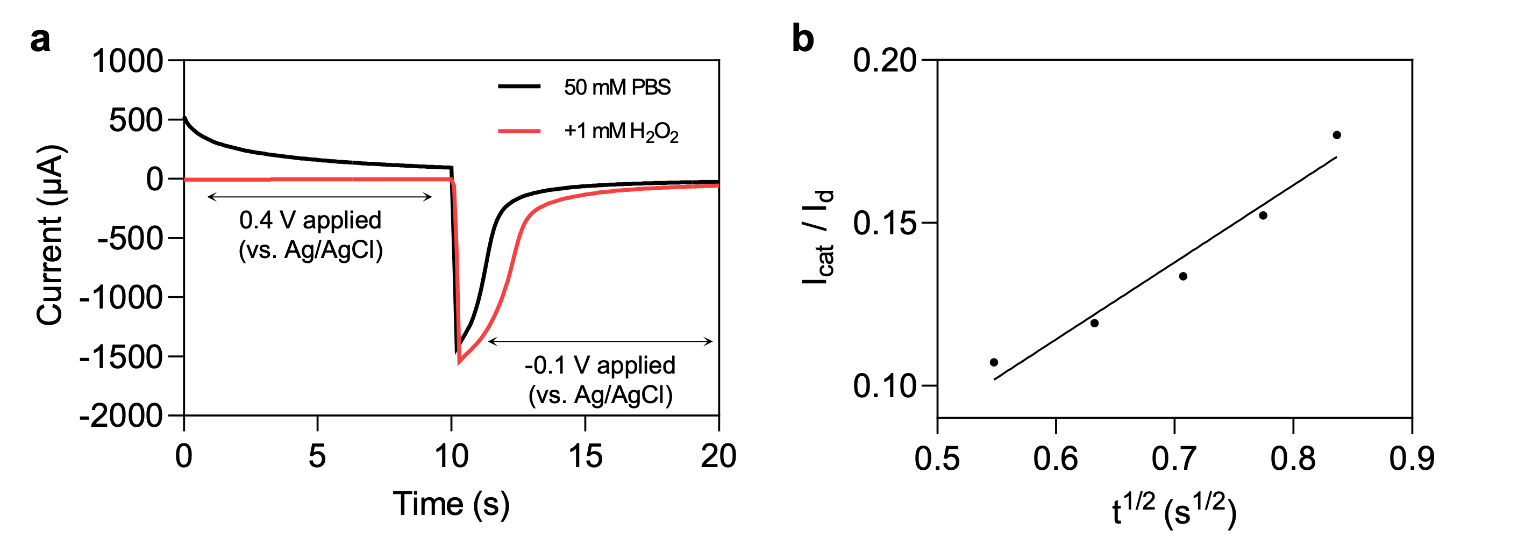


**Figure S18**. a) Amperograms recorded under a two-step potential method (0.4 V for 10 s to -0.1 V for 10 s) in PBS, in the absence (black) and presence (red) of 1.0 mM H_2_O_2_ using C-ECH/SPCE. b) Savéant–Nicholson kinetic analysis for electrochemical reduction of H_2_O_2_ using C-ECH/SPCE obtained from two-step potential measurements, showing the plot of I_cat_ / I_d_ vs. t^1/2^. I_cat_: catalytic current response in the presence of H₂O₂, I_d_: current response in the absence of H_2_O_2_.

**
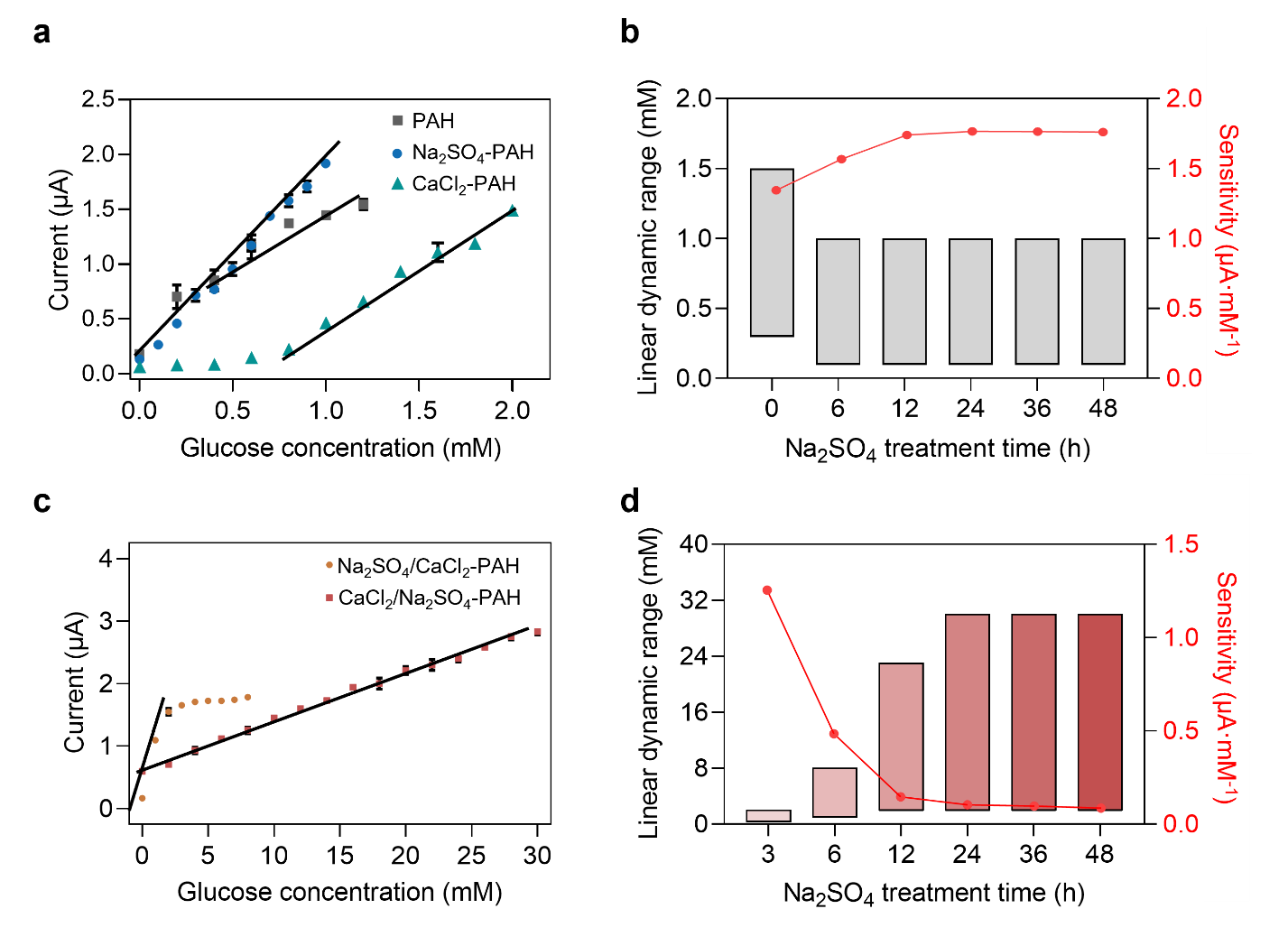
**

**Figure S19**. a) Calibration plots obtained from chronoamperometric (CA) measurements using highly porous ISC–PAH-modified glucose biosensors with a regression equation, (i) PAH, y = 0.8688x + 0.5362, *r^2^* = 0.882; (ii) Na_2_SO_4_–PAH, y = 1.813x + 0.086, *r^2^* = 0.996; (iii) CaCl_2_–PAH, y = 1.018x – 0.5970, *r^2^* = 0.984. b) Linear dynamic range and sensitivity for glucose detection using Na_2_SO_4_–PAH-modified glucose biosensors as a function of Na_2_SO_4_ treatment duration. c) Calibration plots obtained from CA measurements using sequential ion-treated ISC–PAH-modified glucose biosensors with a regression equation, (i) Na_2_SO_4_/CaCl_2_–PAH, y = 0.7105x + 0.2216, *r^2^* = 0.947; (ii) CaCl_2_/Na_2_SO_4_–PAH, y = 0.0773x + 0.6707, *r^2^* = 0.988. d) Box plots showing the effect of Na_2_SO_4_ treatment time on the sensitivity and linear range of CaCl_2_/Na_2_SO_4_–PAH biosensors pretreated with CaCl_2_ for 12 h. CA measurements were performed in glucose solutions (2–30 mM) at –0.1 V (vs. Ag/AgCl). Data represent mean ± SD (*n* = 3).


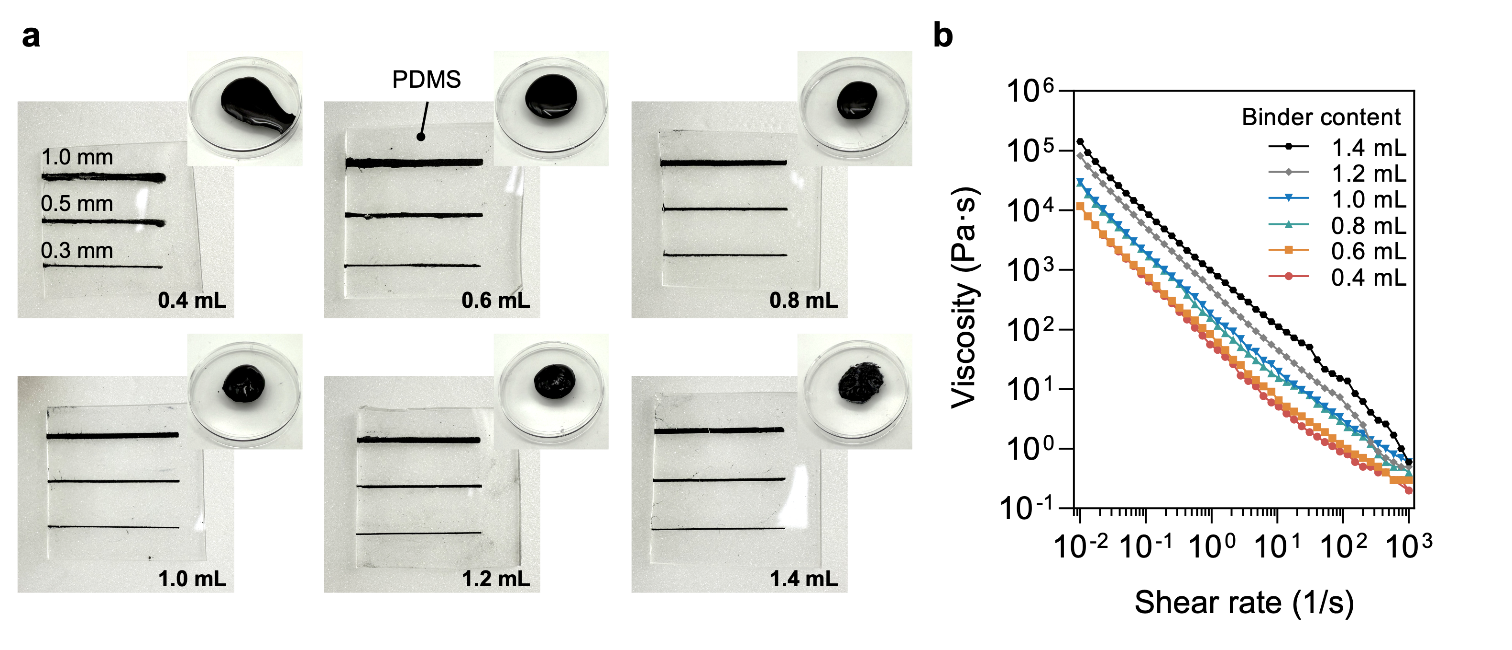


**Figure S20.** a) Photographs of line-printing tests using elastic graphite-filled-PEDOT:PSS (EG-PEDOT:PSS) ink formulated with varying waterborne polyurethane(WPU) contents. b) Rheological properties of EG-PEDOT:PSS ink with various WPU binder contents obtained using a rheometer.

The EG-PEDOT:PSS ink was formulated using graphite and PEDOT:PSS to provide conductivity and electrochemical stability, combined with WPU serving as a stretchable binder. The WPU content was optimized to achieve superior printability. Line-printing tests showed that only the formulation with 1 mL of WPU binder produced smooth and uniform linear patterns across all line widths without spreading or breaking. Rheological analysis confirmed these ideal properties, demonstrating a high zero-shear viscosity for maintaining pattern integrity after printing, coupled with a sufficiently low viscosity under high shear conditions for efficient ink transfer.

**
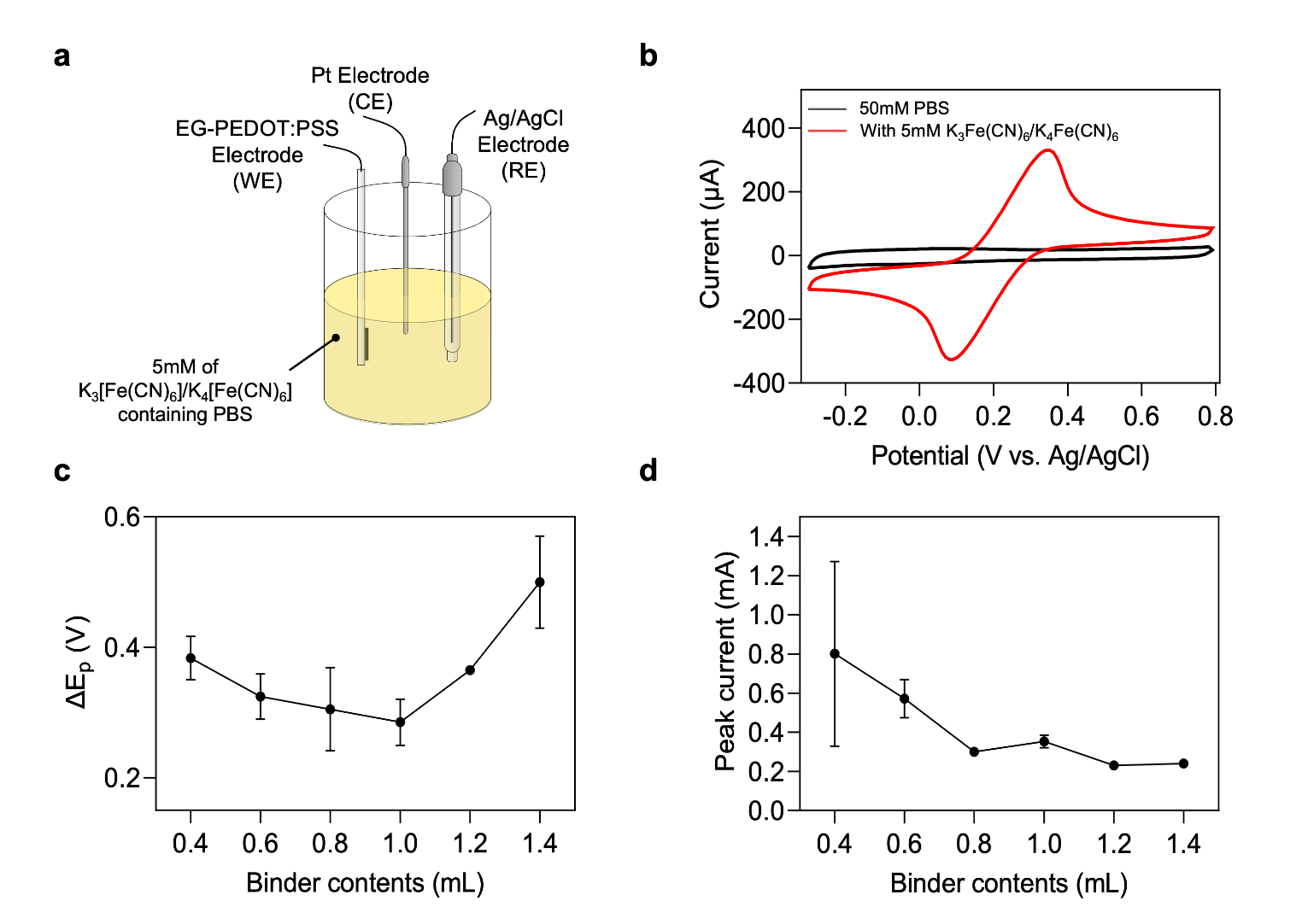
**

**Figure S21.** Electrochemical performance of EG-PEDOT:PSS electrodes prepared with various WPU contents. a) Schematic illustration of the electrochemical measurement system consisting of an EG-PEDOT:PSS electrode as a working electrode, Pt wire as a counter electrode, and Ag/AgCl as a reference electrode. b) Cyclic voltammograms of EG-PEDOT:PSS electrodes prepared using 1 mL of binder (WPU), recorded in the potential range from –0.3 to 0.8 V (vs. Ag/AgCl) at a scan rate of 0.1 V/s in 5 mM K_3_[Fe(CN)_6_]/K_4_[Fe(CN)_6_] solution. c) Variation of peak–to–peak separation (∆E_p_) as a function of binder content, and d) the relationship between peak current and binder contents of EG-PEDOT:PSS electrode, obtained from Cyclic voltammetry (CV) measurements in 5 mM K_3_[Fe(CN)_6_]/K_4_[Fe(CN)_6_] solution. Data are presented as mean ± SD, *n* = 3.

Electrochemical characterization revealed that the formulation with 1 mL WPU exhibited the lowest peak-to-peak separation (ΔE_p_) and highest peak current, indicating superior electron transfer kinetics and electrochemical reversibility.

**
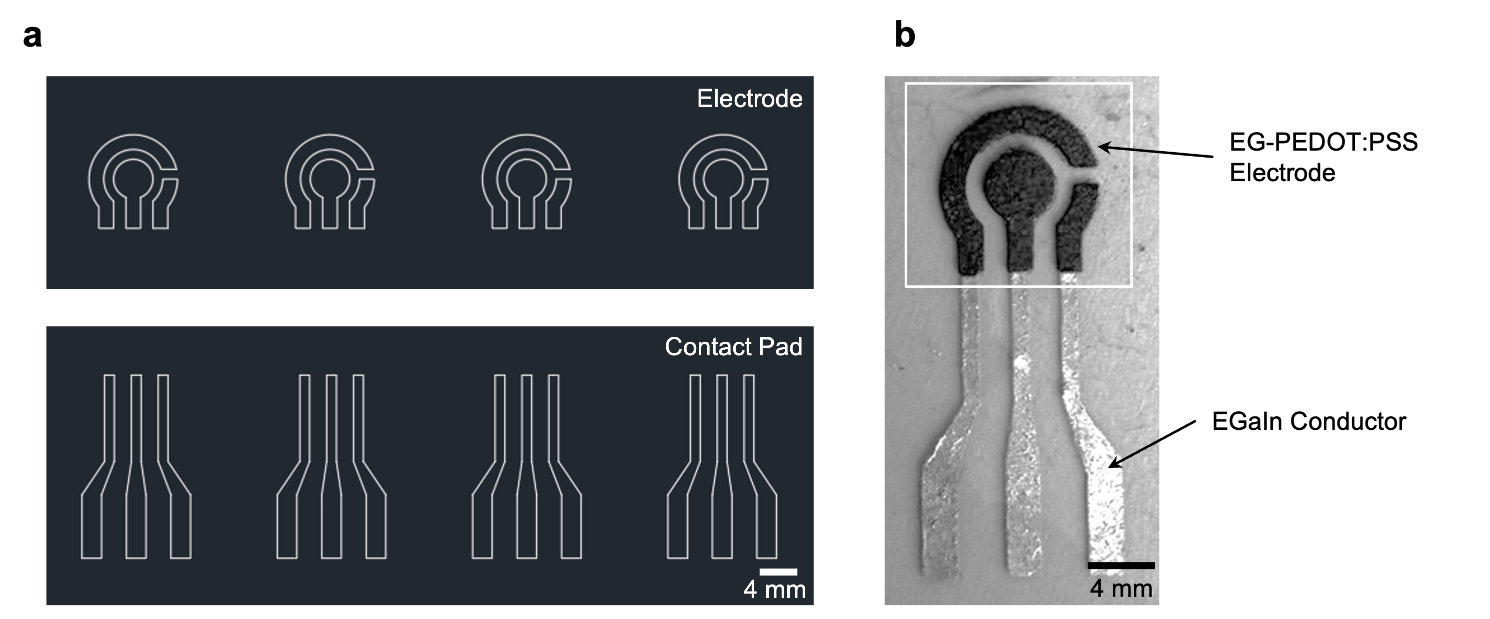
**

**Figure S22.** a) CAD design and b) photograph of EG-PEDOT:PSS electrode featuring a three-electrode configuration with EGaIn conductor.

The optimized EG-PEDOT:PSS ink was utilized to print a three-electrode system with stretchable EGaIn conductive traces onto a stretchable SEBS substrate to fabricate the stretchable electrochemical biosensor. First, to form the soft and highly conductive current collector, a paste-type EGaIn formulation was prepared by sonicating EGaIn alloy (75 wt% Ga and 25 wt% In) at 140 W for 5 min in a water-cooled bath (Qsonica LLC, Newtown, USA). The sonication process disrupted surface oxides and transformed the fluidic metal into a viscous, particle-like paste with enhanced surface adhesion, making it suitable for stencil printing on stretchable substrates.


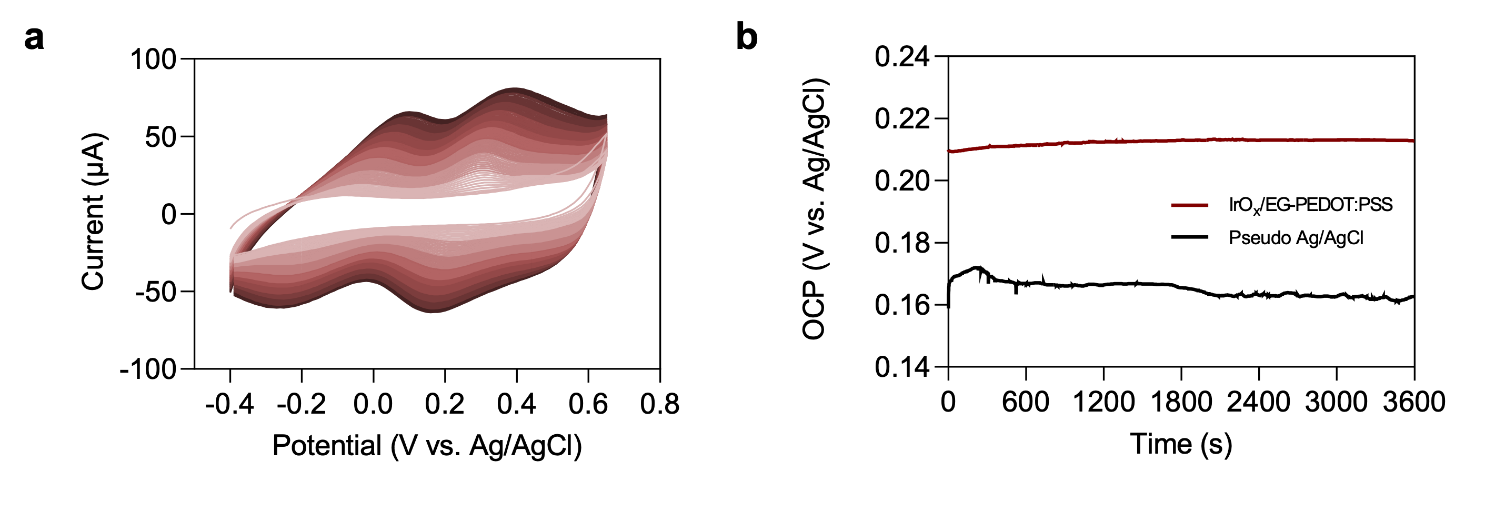


**Figure S23.** Fabrication and evaluation of electrochemical stability of IrO_x_ reference electrodes**.** a) Cyclic voltammograms recorded during electrodeposition of IrO_x_ on EG-PEDOT:PSS electrodes in the potential range from –0.4 to 0.6 V at a scan rate of 0.1 V/s. b) Open circuit potential (OCP) of IrO_x_-modified EG-PEDOT:PSS and commercial Ag/AgCl printed pseudo-reference electrode in PBS.

To develop a stable and biocompatible reference electrode for stretchable electrochemical biosensors, IrO_x_ was chosen for its high chemical stability, minimal potential drift, and excellent biocompatibility.^[3,4]^ Electrodeposition of IrO_x_ was performed following previous studies.^[5]^ The electrochemical deposition was performed using an IrO_x_ precursor solution (0.15 g iridium tetrachloride dissolved in 100 mL DI water, with sequential additions of 1 mL H_2_O_2_ solution (30 wt%), 0.5 g oxalic acid, and K_2_CO_3_ to adjust pH to 10.5) via CV from -0.2 to 0.65 V (vs. Ag/AgCl) with a Pt counter electrode and an Ag/AgCl reference electrode at a scan rate of 0.1 V/s for 80 cycles, followed by rinsing the electrode with DI water. Then, OCP measurements were conducted for 1 h to evaluate the reference electrode stability. The fabricated IrO_x_ reference electrode exhibited outstanding electrochemical stability, demonstrating a potential drift of less than ±10 mV over 1 h, a significant improvement compared to commercial Ag/AgCl ink-based electrodes, which typically exhibit potential drifts greater than ±20 mV.


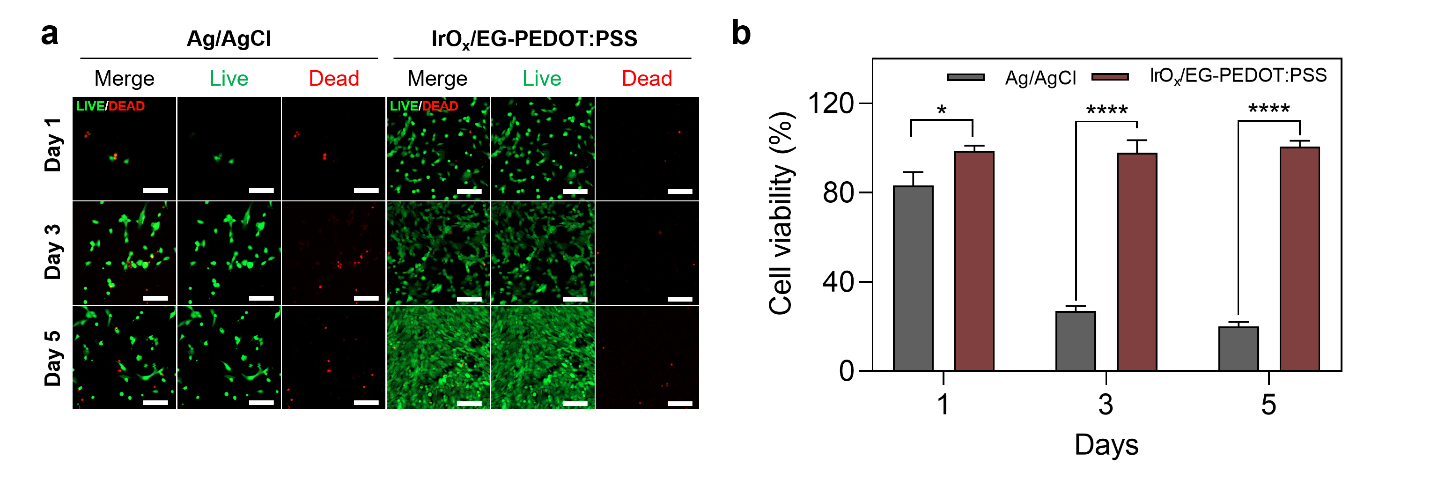


**Figure S24.** a) Live/Dead fluorescence staining images of NIH/3T3 cells cultured on Ag/AgCl and IrO_x_/EG-PEDOT:PSS electrodes for 1, 3, and 5 days (live cells are stained green, and dead cells are stained red; Scale bar = 50 μm). b) In vitro biocompatibility test: Quantitative analysis of cell viability (%) over 5 days, confirming the significantly higher biocompatibility of IrO_x_/EG-PEDOT:PSS compared with Ag/AgCl. Data are presented as mean ± SD, *n* = 3. (**p* < 0.05, ***p* < 0.01, ****p*< 0.001, and *****p* < 0.0001; ns, not significant).

Cell viability assessments confirmed that the IrO_x_ electrode possesses superior biocompatibility compared to Ag/AgCl electrodes, ensuring its suitability and safety for skin-contact applications. As described in the Experimental section, fibroblast cells were co-cultured with each electrode using a transwell system, and viability was evaluated using Live/Dead staining and CCK-8 assays.


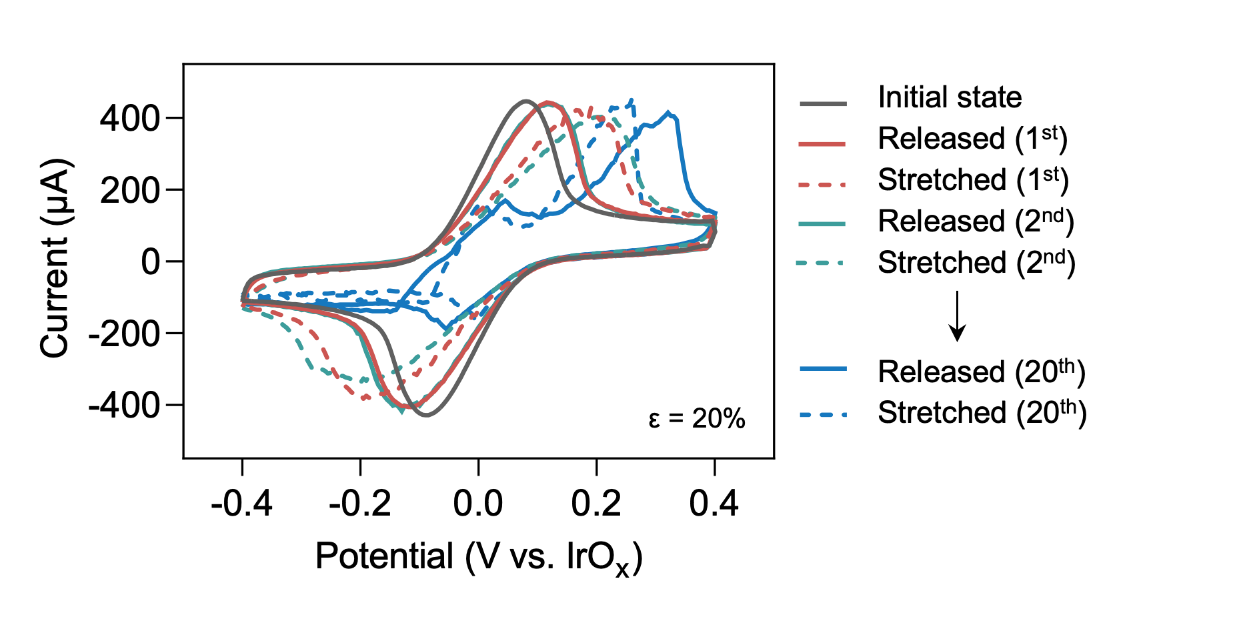


**Figure S25.** Cyclic voltammograms recorded during repeated stretch–release cycles (*ε* = 20%) using an EG-PEDOT:PSS working electrode in a three-electrode configuration with an IrO_x_/EG-PEDOT:PSS reference electrode. CV measurements were conducted in a 5 mM K_3_Fe(CN)_6_/K_4_Fe(CN)_6_ solution over a potential range from –0.4 to 0.4 V (vs. Ag/AgCl) at a scan rate of 0.1 V/s.

##
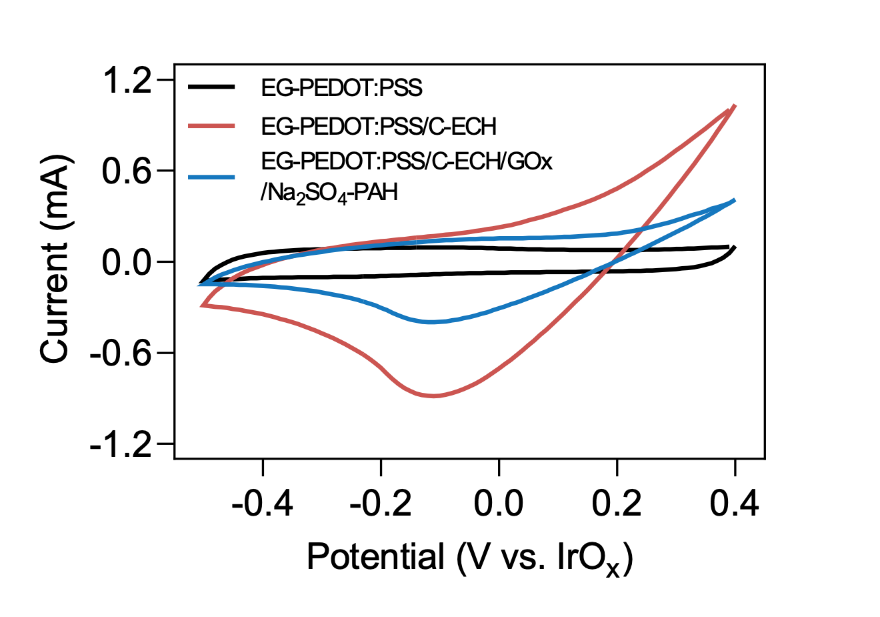


**Figure S26.** Cyclic voltammograms recorded with an EG-PEDOT:PSS, C-ECH/EG:PEDOT:PSS, and Na_2_SO_4_–PAH/GO_x_/C-ECH/EG-PEDOT:PSS in PBS. CV measurements were conducted in the potential range from –0.5 to 0.4 V (vs. IrO_x_) at a scan rate of 0.1 V/s.

The stepwise fabrication of the glucose biosensor on the optimized EG-PEDOT:PSS electrode was investigated through CV. A clear increase in capacitance and distinct redox peaks were observed after C-ECH coating on EG-PEDOT:PSS electrodes. After the subsequent modification of GO_x_ and Na₂SO₄–PAH, a decrease in these redox peaks was observed. This occurs because the additional ISC–PAH (Na_2_SO_4_–PAH) limits the access of K^+^ ions, which participate in the redox reactions of PB and Ni-PB, as shown in equations (S1) and (S2) below.^[6,7]^

| ${{Fe}_{4}}^{III}\left[ {Fe}^{II}\left( CN \right)_{6} \right]^{3+}K^{+}+e^{-} \leftrightarrow K{Fe}^{III}{Fe}^{II}\left( CN \right)_{6}$  ${K_{x}{Ni}^{II}[Fe}^{III}\left( CN \right)_{6} +{\left( 1-x \right)K^{+}+ e}^{-} \leftrightarrow K_{x+1}{Ni}^{II}[{Fe}^{II}\left( CN \right)_{6}]$ | (S1)  (S2) |
| --- | --- |
|  |  |

**
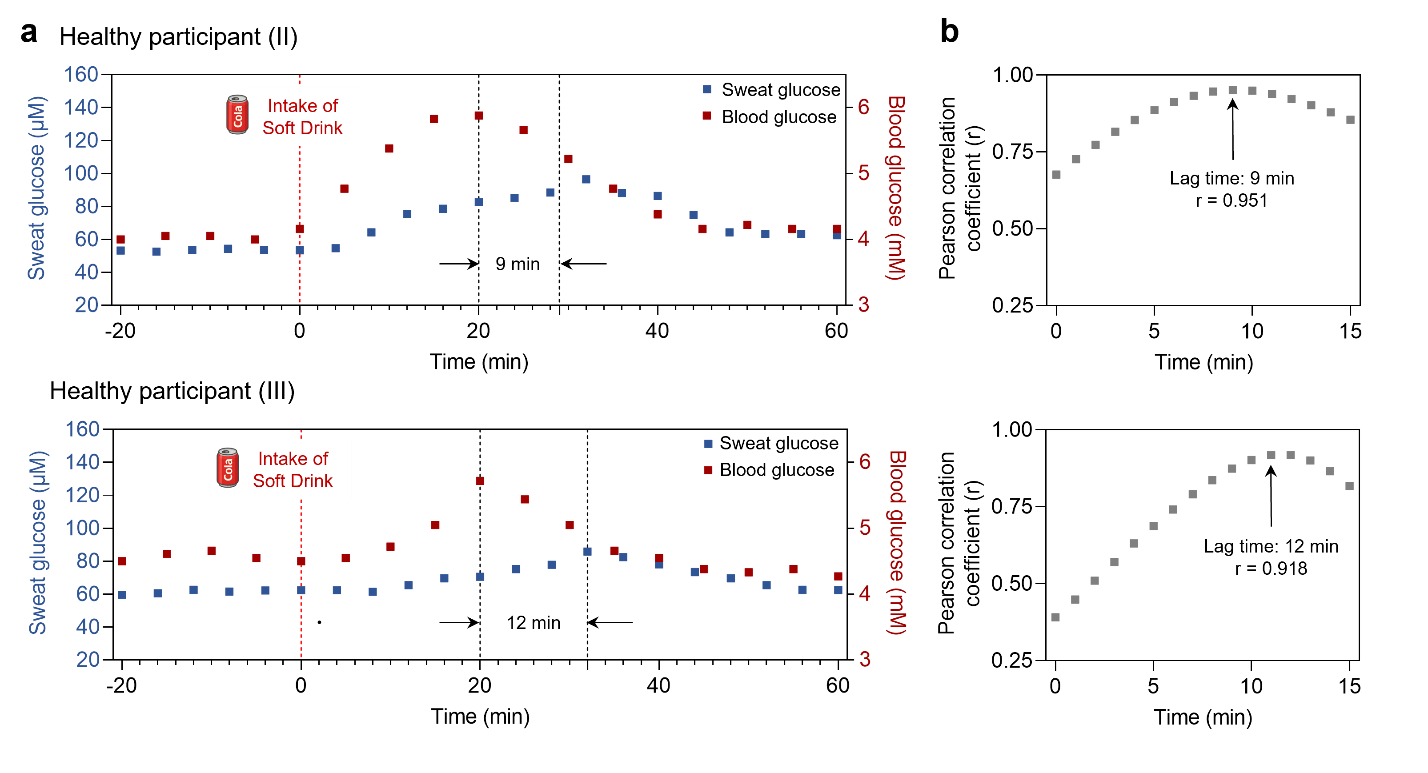
**

**Figure S27.** a) Continuous measurements of sweat glucose (SG) and blood glucose (BG) for each healthy participant (II-III) with intake of soft drink. b) Corresponding Pearson correlation coefficient (*r*) between SG and BG as a function of lag time, derived from the results of continuous SG and BG monitoring.


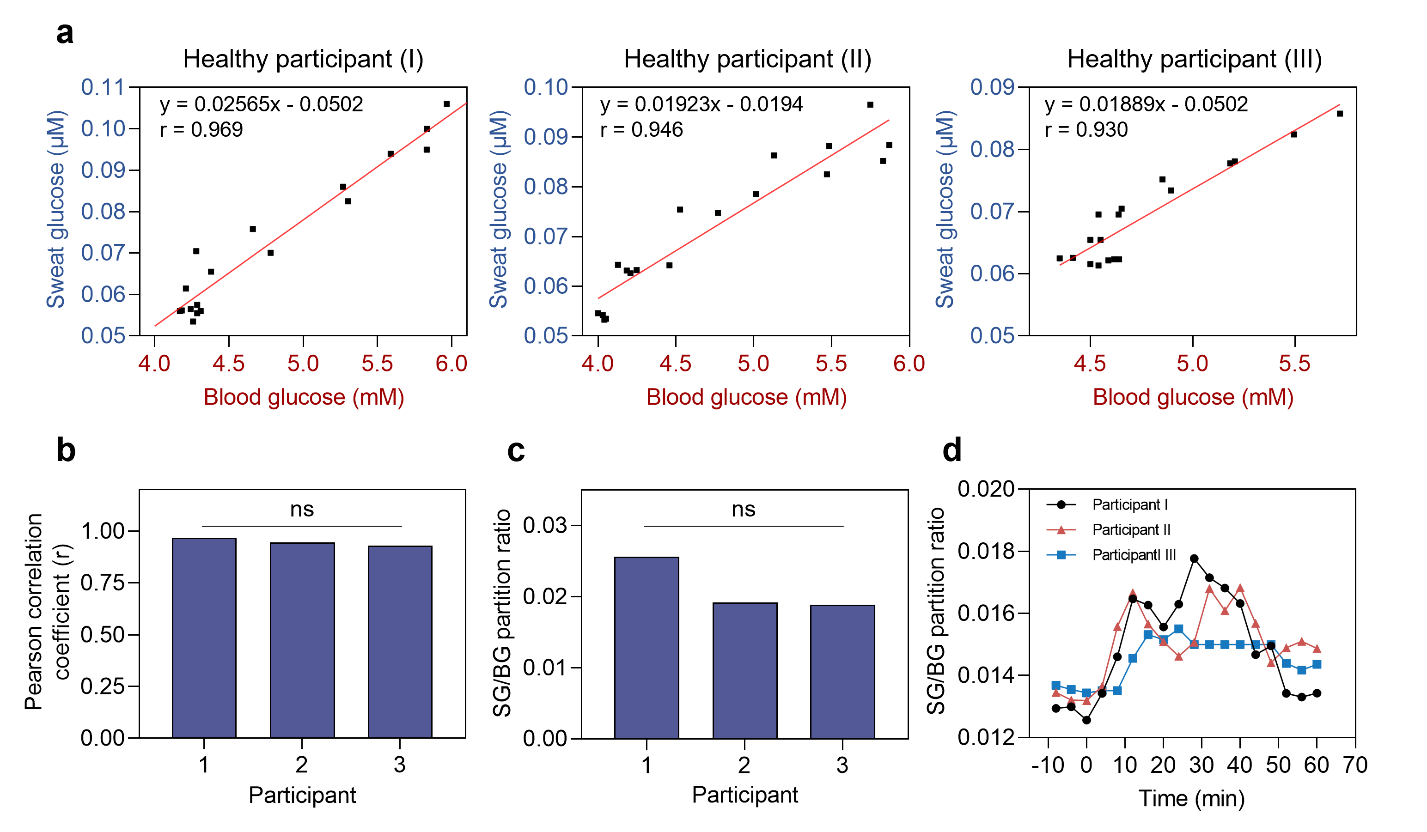


**Figure S28.** a) Scatter plot showing the relationship between sweat glucose (SG) and blood glucose (BG) levels for a) Participant (I), (II), and (III). b) Pearson correlation coefficient (*r*) across participants. c) Inter-participant variability and d) time-dependent changes in the SG/BG partition ratio across individual participants with soft drink intake at 0 min. (**p* < 0.05, ***p* < 0.01, ****p* < 0.001, *****p* < 0.0001; ns, not significant).

Across participants, the Pearson correlation coefficients (*r*) between SG and BG were comparable and showed no statistically significant differences (*p* > 0.05), with a low coefficient of variation (CVs = 15.5 %), confirming consistent SG/BG coupling strength among individuals (**Figure S28b**). A linear mixed-effects (LME) analysis further revealed a significant fixed effect of time (*p* = 6.0 × 10⁻⁶), whereas the random participant effect and its interaction with time were not significant (*p* > 0.05), indicating that all participants shared a common temporal trajectory (**Figure S28c, d**). Despite dynamic variation, the SG/BG partition ratio followed a reproducible baseline → postprandial peak → recovery trajectory. Relative to baseline, the ratio transiently increased during the postprandial phase and gradually returned toward baseline, reflecting a synchronized physiological regulation between systemic glucose uptake and sweat secretion. This behavior supports pooling of lag-corrected data to represent a shared physiological dynamic. Mechanistically, the observed trend is consistent with concentration-gradient-driven transport, in which meal-induced elevations in blood glucose steepen the interstitial-to-sweat gradient, transiently enhancing glucose flux and elevating the ratio.^[8,9]^


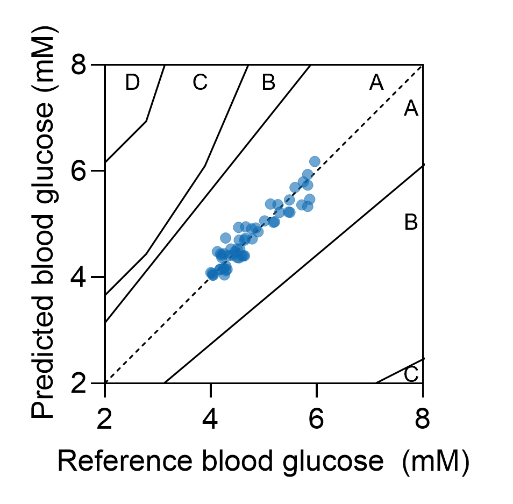


**Figure S29.** Parkes error grid analysis of three healthy participants using Na_2_SO_4_–PAH-modified wearable glucose biosensors.


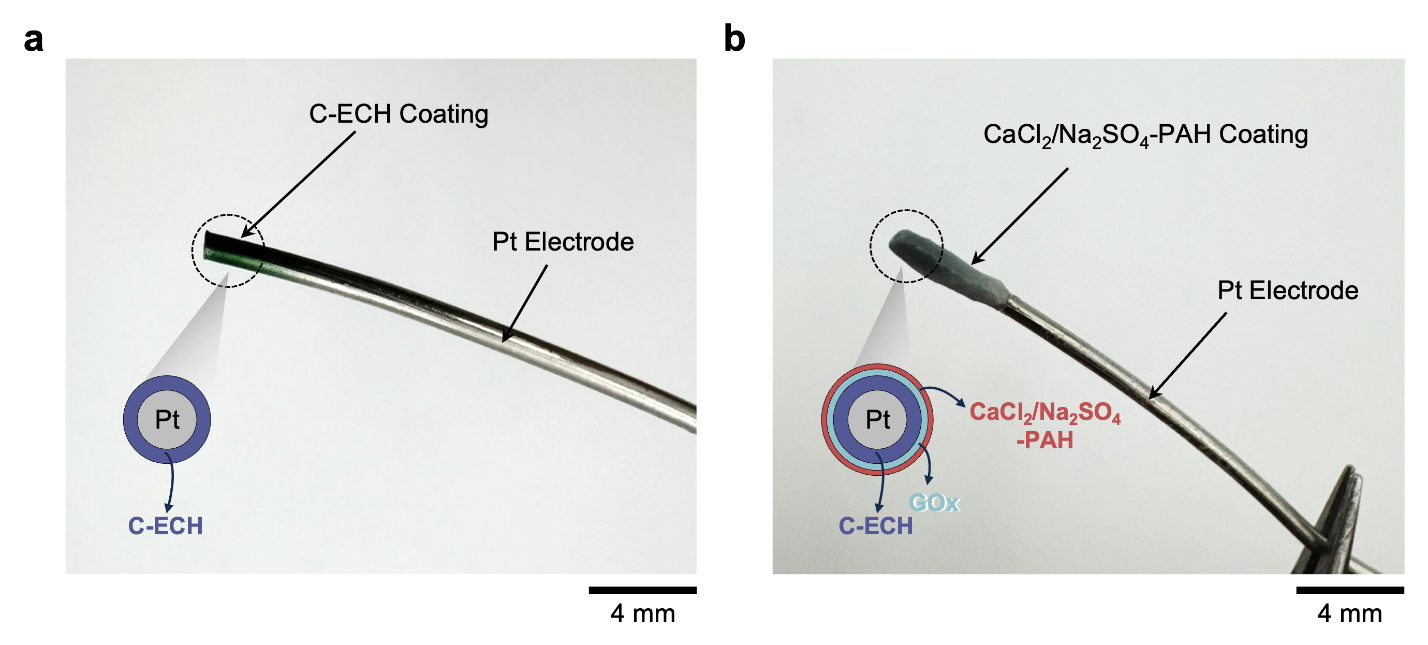


**Figure S30.** Photographs of a) C-ECH/Pt electrode and b) CaCl_2_/Na_2_SO_4_–PAH/GO_x_/C-ECH/Pt electrode.

**
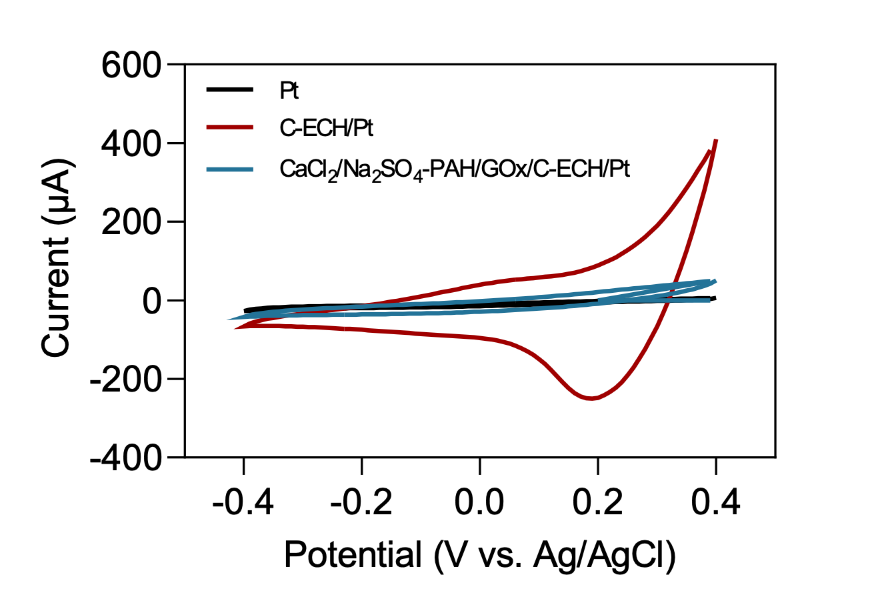
**

**Figure S31.** Cyclic voltammograms recorded with a Pt electrode, C-ECH/Pt electrode, and CaCl_2_/Na_2_SO_4_–PAH/GO_x_/C-ECH/Pt electrode in PBS. CV measurements were conducted in the potential range from –0.4 V to 0.4 V (vs. Ag/AgCl) at a scan rate of 0.1 V/s.

The stepwise fabrication process of the implantable glucose biosensor was evaluated by CV. Compared with the unmodified Pt electrode, distinct oxidation/reduction peaks of Ni-PB nanoparticles were observed for the C-ECH/Pt electrode. However, on the final CaCl_2_/Na_2_SO_4_–PAH/GO_x_/C-ECH/Pt electrode, the cathodic current was significantly reduced. This current decrease is interpreted, based on equations S4 and S5 as the result of limited access for K⁺ ions, which participate in the electrochemical reactions within the C-ECH, due to the high diffusion resistance of the outer CaCl_2_/Na_2_SO_4_–PAH layer.


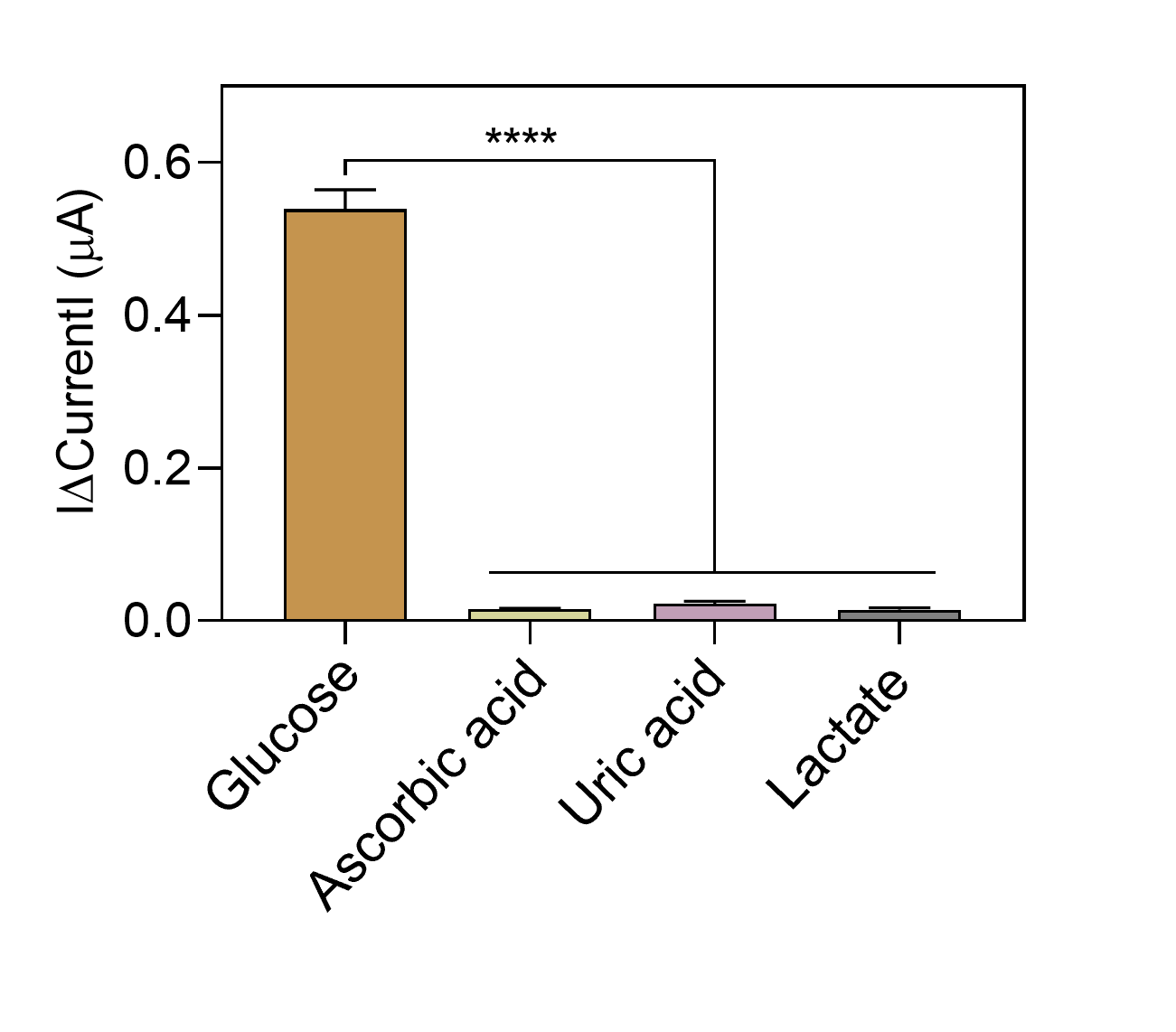


**Figure S32**. Bar plots obtained from chronoamperometry at an applied potential of –0.1 V (vs. Ag/AgCl) illustrating the selectivity of the CaCl_2_/Na_2_SO_4_–PAH/C-ECH/Pt electrode under the sequential addition of 5 mM glucose, 50 µM ascorbic acid, 50 µM uric acid, and 1 mM lactate. Selectivity was evaluated by comparing the steady-state amperometric current responses after the addition of each analyte. Data are presented as mean ± SD, *n* = 3. (**p* < 0.05, ***p* < 0.01, ****p* < 0.001, and *****p* < 0.0001; ns, not significant).


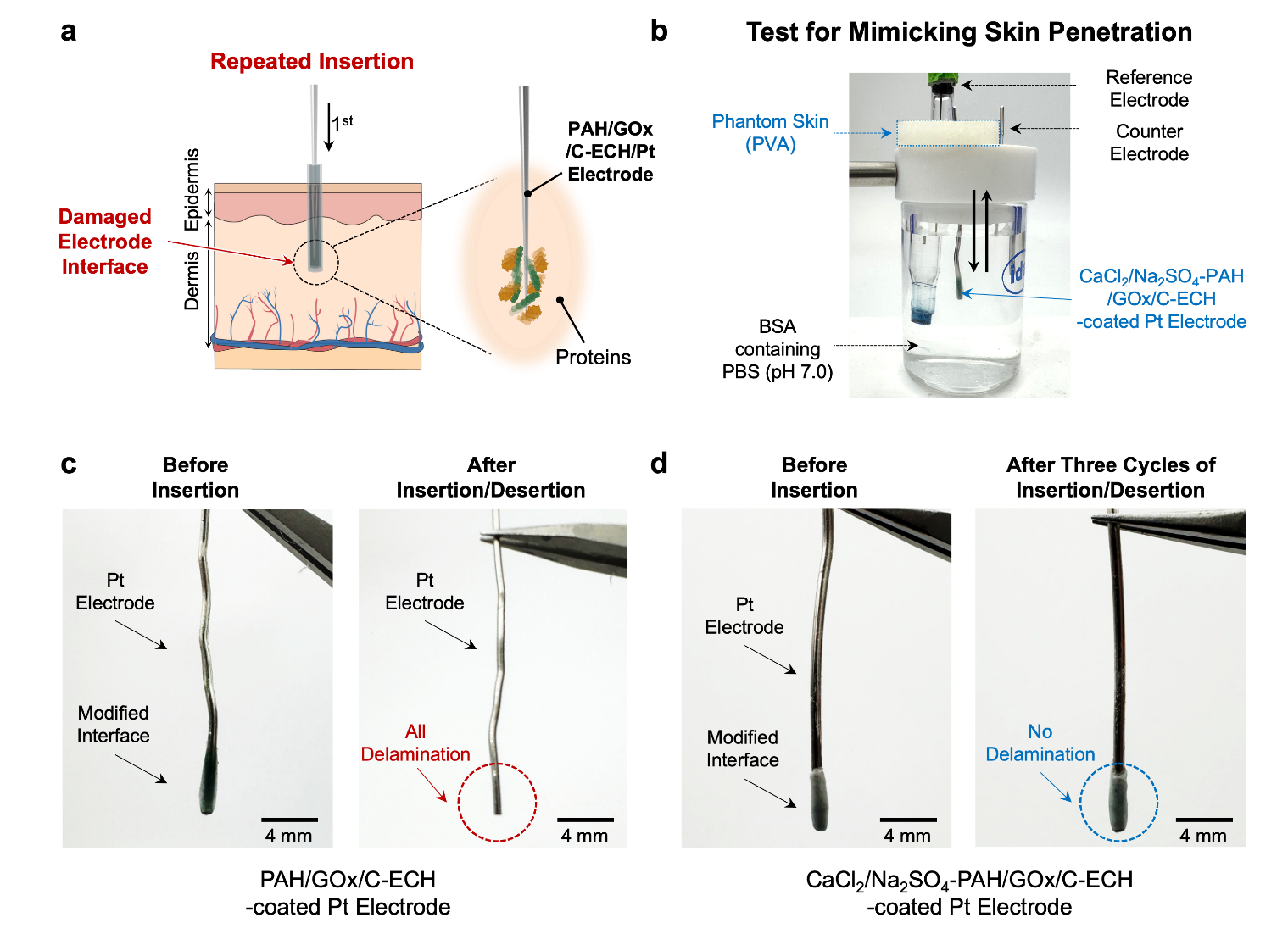


**Figure S33**. a) Schematic representation highlighting potential interface damage or structural disruption of the ISC–PAH–modified glucose biosensor interface (PAH/GO_x_/C-ECH/Pt) after electrode insertion of into skin. b) Photographs of a test simulating skin-penetration using phantom skin with CaCl_2_/Na_2_SO_4_–PAH/GO_x_/C-ECH/Pt electrode. c) Photograph comparing the PAH/GO_x_/C-ECH/Pt electrode before and after one cycle of insertion/removal into phantom skin. d) Photograph comparing the CaCl_2_/Na_2_SO_4_–PAH/GO_x_/C-ECH/Pt electrode before and after three cycles of insertion/removal into phantom skin.


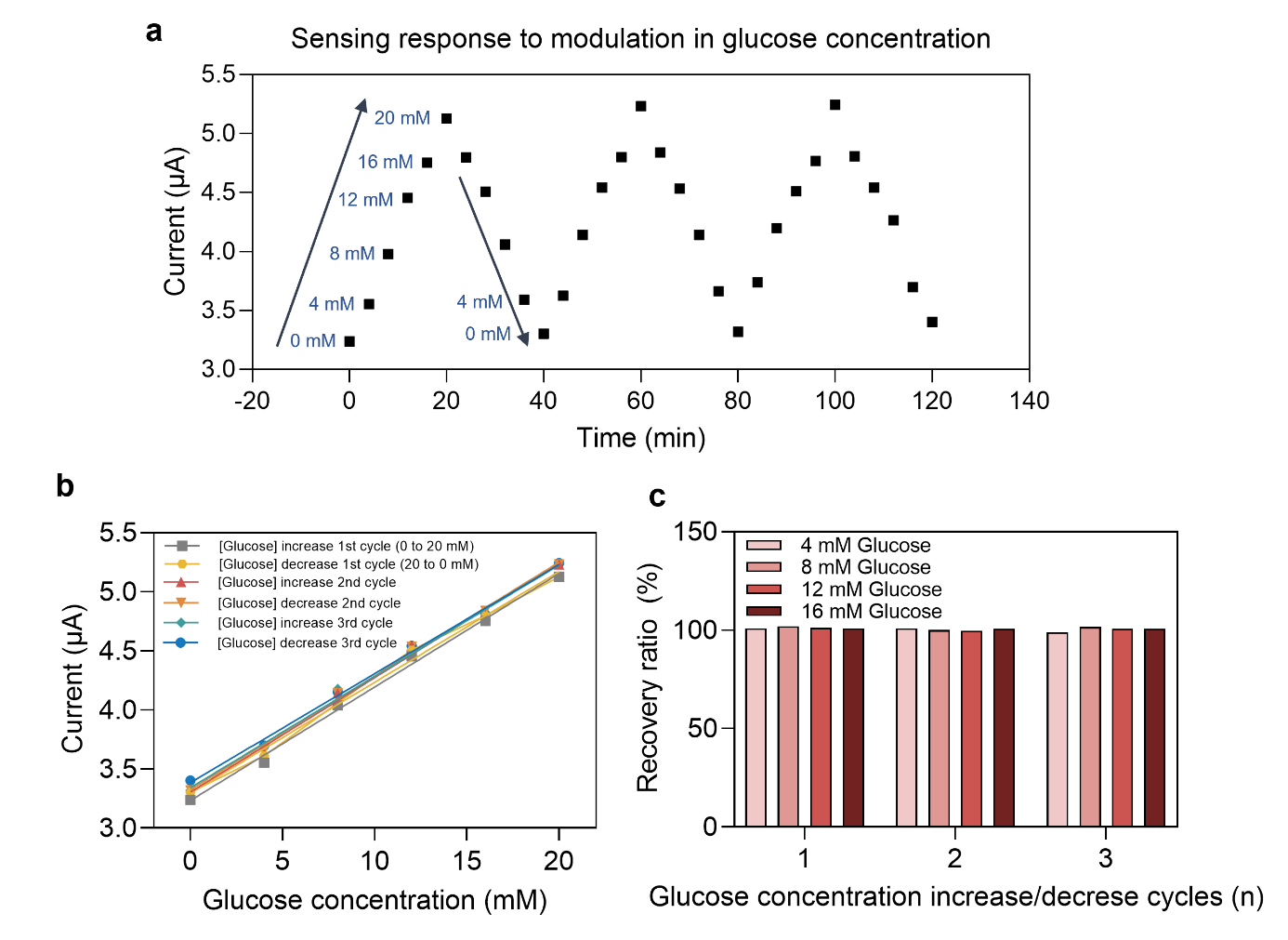


**Figure S34.** a) Stepwise current responses of the CaCl_2_/Na_2_SO_4_–PAH/GO_x_/C-ECH/Pt electrode under successive increases and decreases in glucose concentration (0–20 mM), b) calibration plots obtained from consecutive increase and decrease steps over three repeated sensing cycles, and c) bar graphs summarizing the signal recovery ratio (%) at each glucose concentration. All data were obtained from chronoamperometric measurements, in which each concentration step consisted of a 3 min incubation for the enzymatic reaction followed by a 1 min measurement, resulting in a 4 min interval between successive increase and decrease steps.


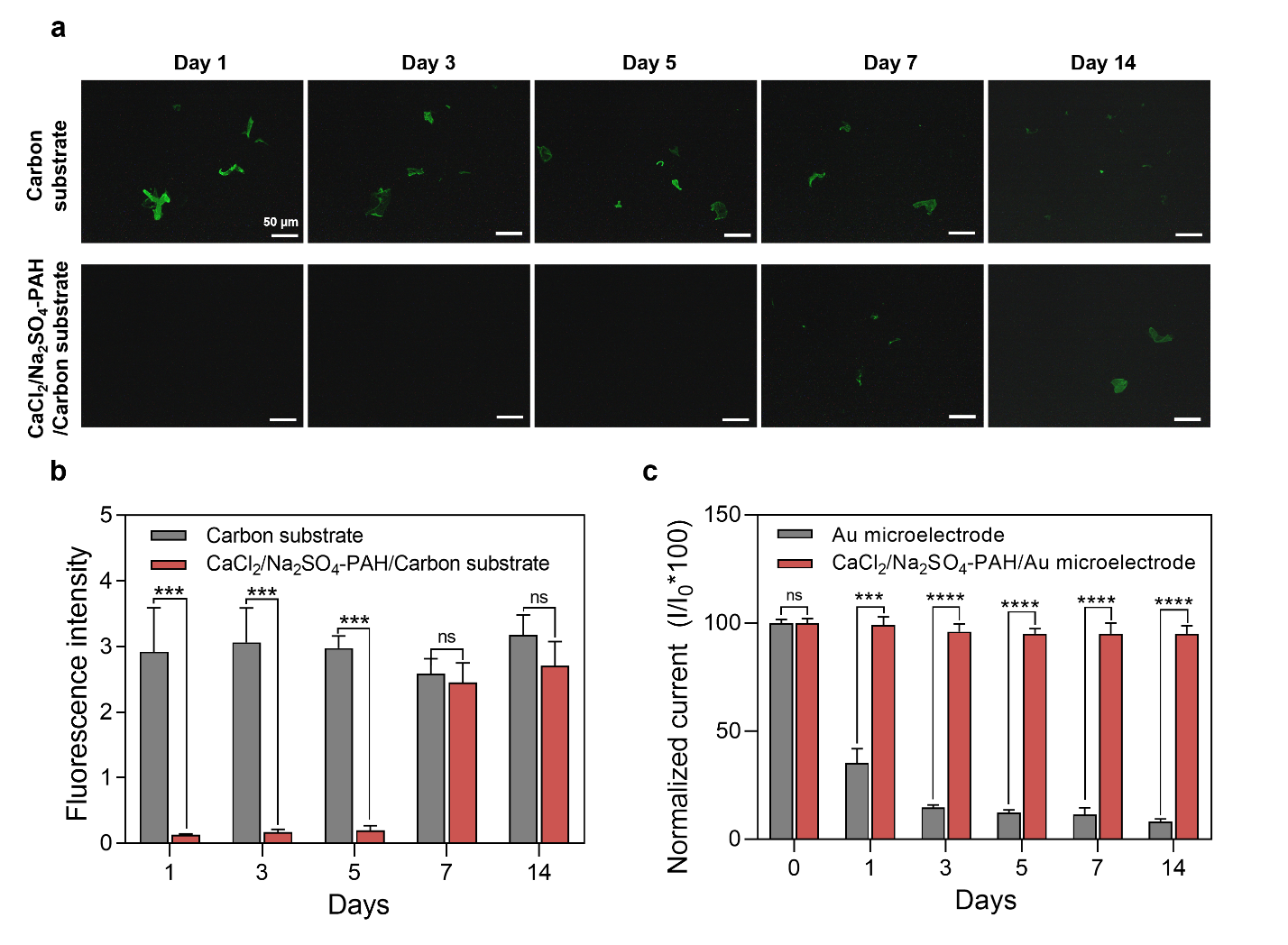


**Figure S35.** a) Representative fluorescence microscopy images of FITC‐labeled bovine serum albumin (FITC–BSA), b) quantitative comparison of FITC–BSA fluorescence intensity between the pristine carbon substrate and CaCl_2_/Na_2_SO_4_–PAH-modified carbon substrate, and c) temporal changes in steady-state currents obtained from linear sweep voltammetry in 5 mM K₃[Fe(CN)₆]/K₄[Fe(CN)₆] (PBS, pH 7.0) containing 5 mg mL⁻¹ BSA using an Au microelectrode (diameter: 12.5 µm) and a CaCl_2_/Na_2_SO_4_–PAH/Au microelectrode. Data are presented as mean ± SD, *n* = 3. (**p* < 0.05, ***p* < 0.01, ****p* < 0.001, and *****p* < 0.0001; ns, not significant).


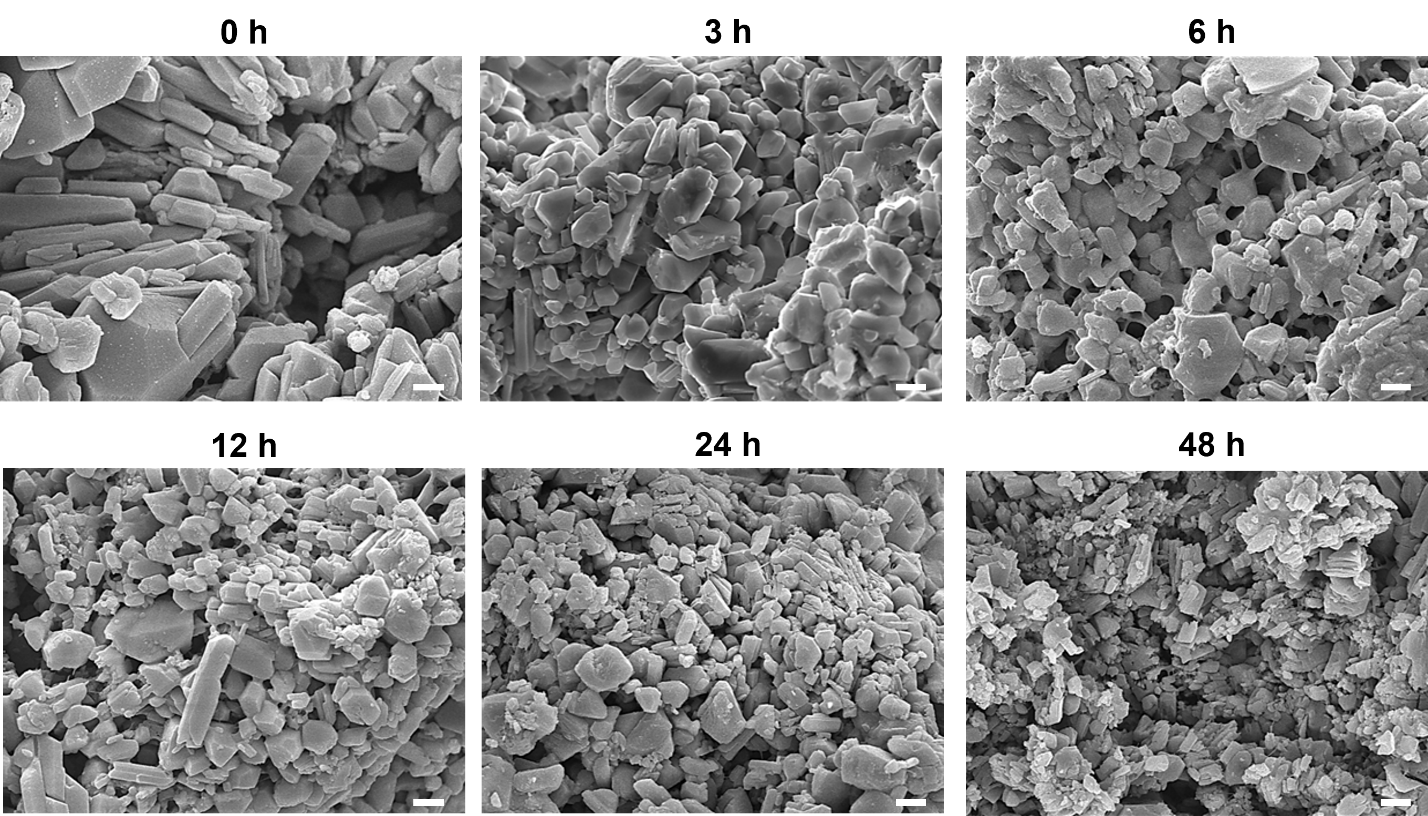


**Figure S36**. Time-resolved SEM images illustrating the morphological evolution of CaSO₄ crystals formed within CaCl_2_/Na_2_SO_4_–PAH during incubation in artificial interstitial fluid (a-ISF) at different time points (0, 3, 6, 12, and 24 h). The hydrogels were sequentially immersed in 2 M CaCl_2_ for 12 h and 1.5 M Na_2_SO_4_ for 24 h. (Scale bar = 1.5 µm).


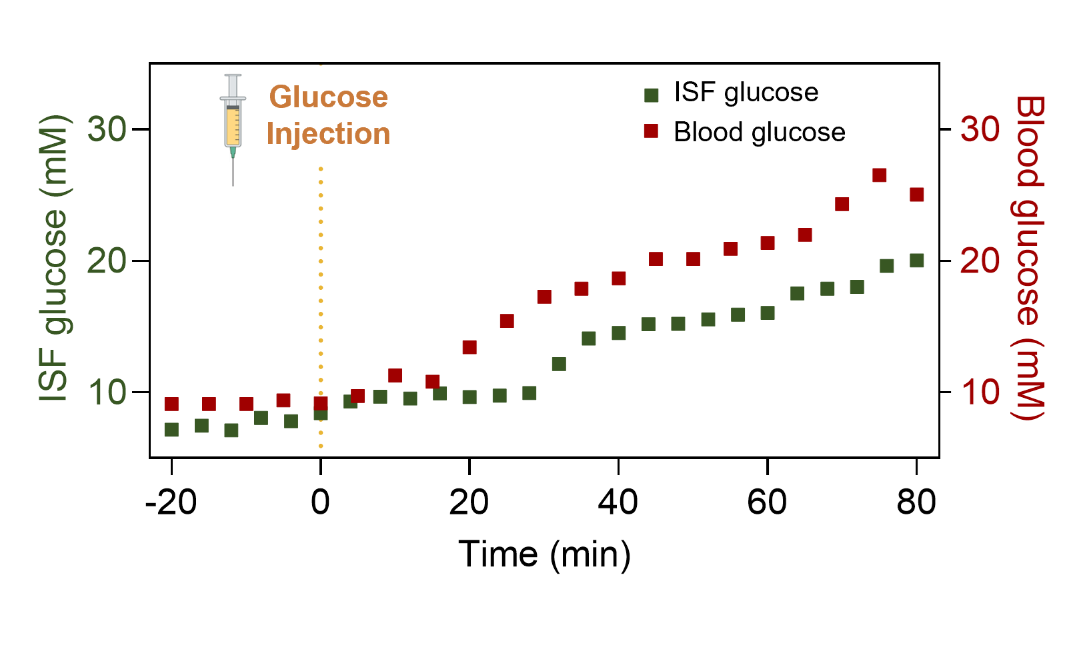


**Figure S37.** Continuous monitoring of interstitial fluid glucose (IG) and blood glucose (BG) in a mouse without insulin injection using CaCl_2_/Na_2_SO_4_–PAH/GO_x_/C-ECH/Pt electrode.


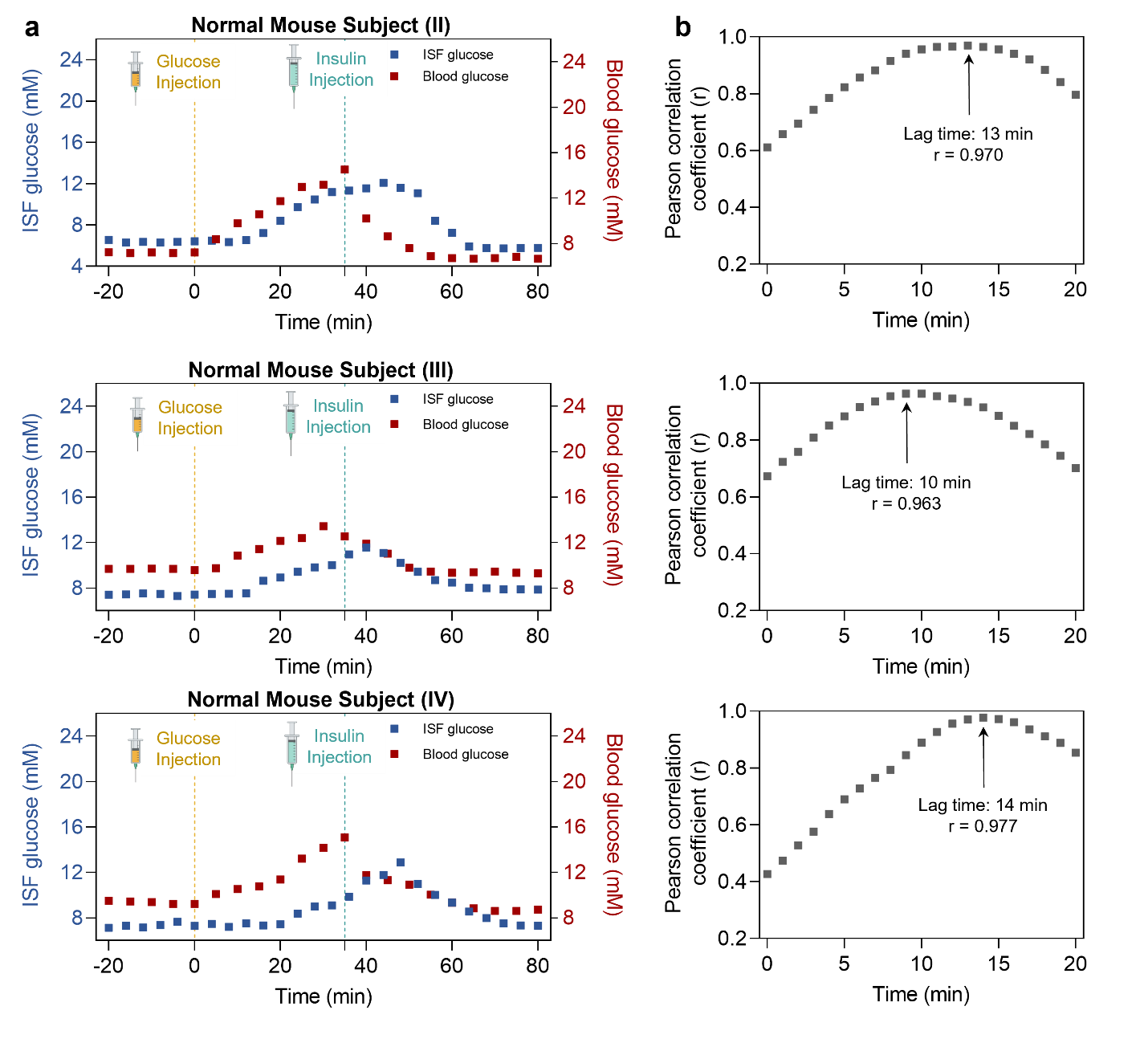


**Figure S38.** a) Results from continuous interstitial fluid glucose (IG) and blood glucose (BG) measurements in normal mice (II–IV) after glucose and insulin injections, and b) corresponding Pearson correlation coefficients (*r*) between IG and BG as a function of lag time.


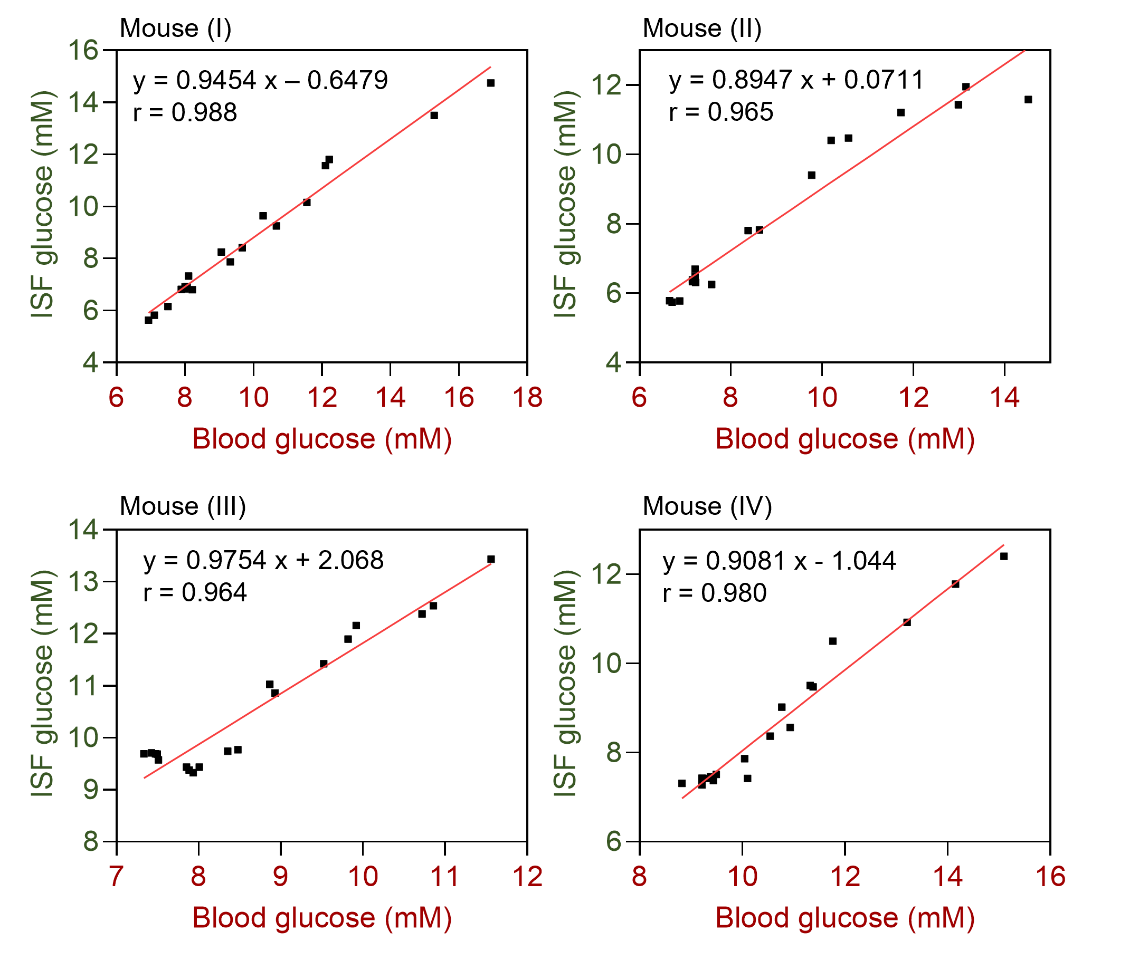


**Figure S39.** Scatter plot showing the relationship between interstitial fluid glucose (IG) and blood glucose (BG) levels of each mouse (I–IV).

Individual mouse data demonstrated strong positive correlations between IG and BG, with Pearson correlation coefficients (*r*) of 0.988, 0.965, 0.964, and 0.980 for mice I, II, III, and IV, respectively (**Figure S39**).


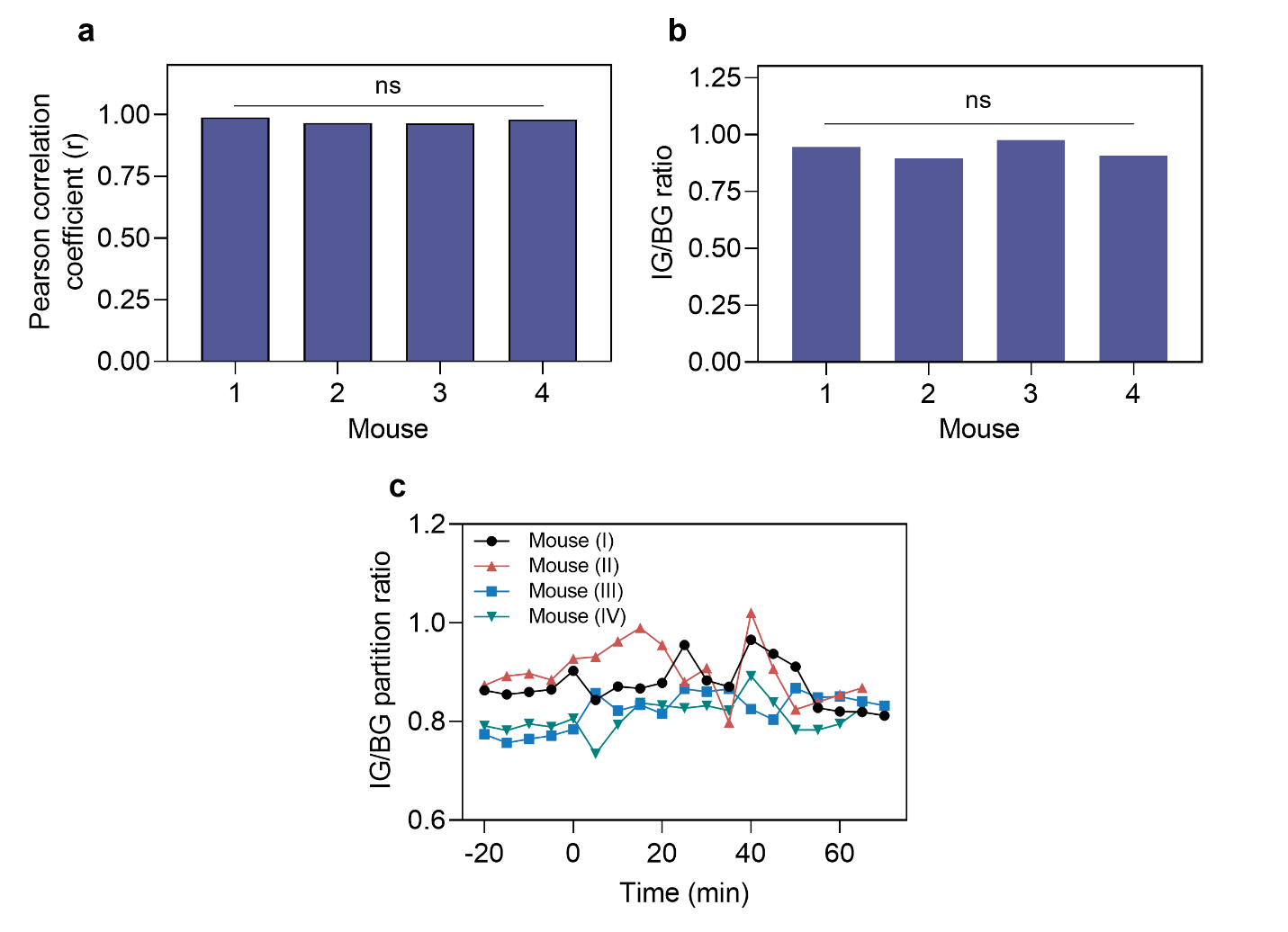


**Figure S40.** a) Pearson correlation coefficients (*r*) across different mice. b) Inter-animal variability and c) time-dependent changes in the IG/BG partition ratio across individual mice with glucose (0 min) – insulin (35 min) injections. (**p* < 0.05, ***p* < 0.01, ****p* < 0.001, and *****p* < 0.0001; ns, not significant).

Pairwise statistical comparisons using the Fisher *r*-to-*z* transformation revealed no significant differences among mice (all *p* > 0.05, **Figure S40a**), indicating that the IG–BG correlation strength was comparable across individuals. This consistency demonstrates that the sensor reliably captures the IG–BG relationship across different animals, ensuring reproducible measurements following appropriate time-lag correction.

After mouse-specific time-lag correction (Mouse I: 9 min, II: 13 min, III: 12 min, and IV: 14 min), both inter-animal variation and the temporal evolution of the IG/BG partition ratio were evaluated (**Figure S40b**). The estimated partition ratios for the four mice were 0.945, 0.895, 0.975, and 0.908 (mean = 0.931; CVs ≈ 3.3%). A linear mixed-effects model revealed significant inter-animal variability (*p* < 0.001) along with a significant main effect of time (*p* < 0.001), but no significant time × mouse interaction (*p* > 0.05), indicating that while mean partition ratios differed across animals, the temporal pattern was conserved.

Specifically, the IG/BG partition ratio transiently increased following glucose administration, reached a postprandial peak, and then partially returned toward baseline. This synchronized trajectory (increase → peak → decline) was consistently observed in all mice, suggesting a conserved physiological response rather than animal-specific fluctuation. This behavior aligns with previous findings showing that interstitial glucose exhibits rate-dependent delays relative to blood glucose, with longer lags during rapid glycemic fluctuations.^[10-13]^ Although absolute ratio levels varied among mice, the preservation of a shared temporal pattern supports pooling of the lag-corrected data to represent a common physiological dynamic.


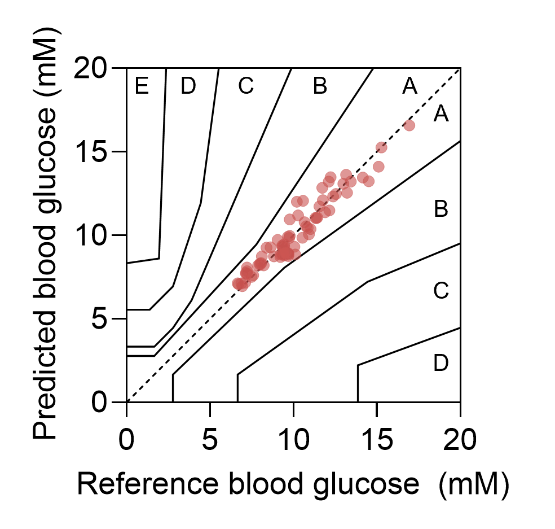


**Figure S41.** Parkes error grid analysis of four normal mice using CaCl_2_/Na_2_SO_4_–PAH/GO_x_/C-ECH/Pt-based glucose biosensors.


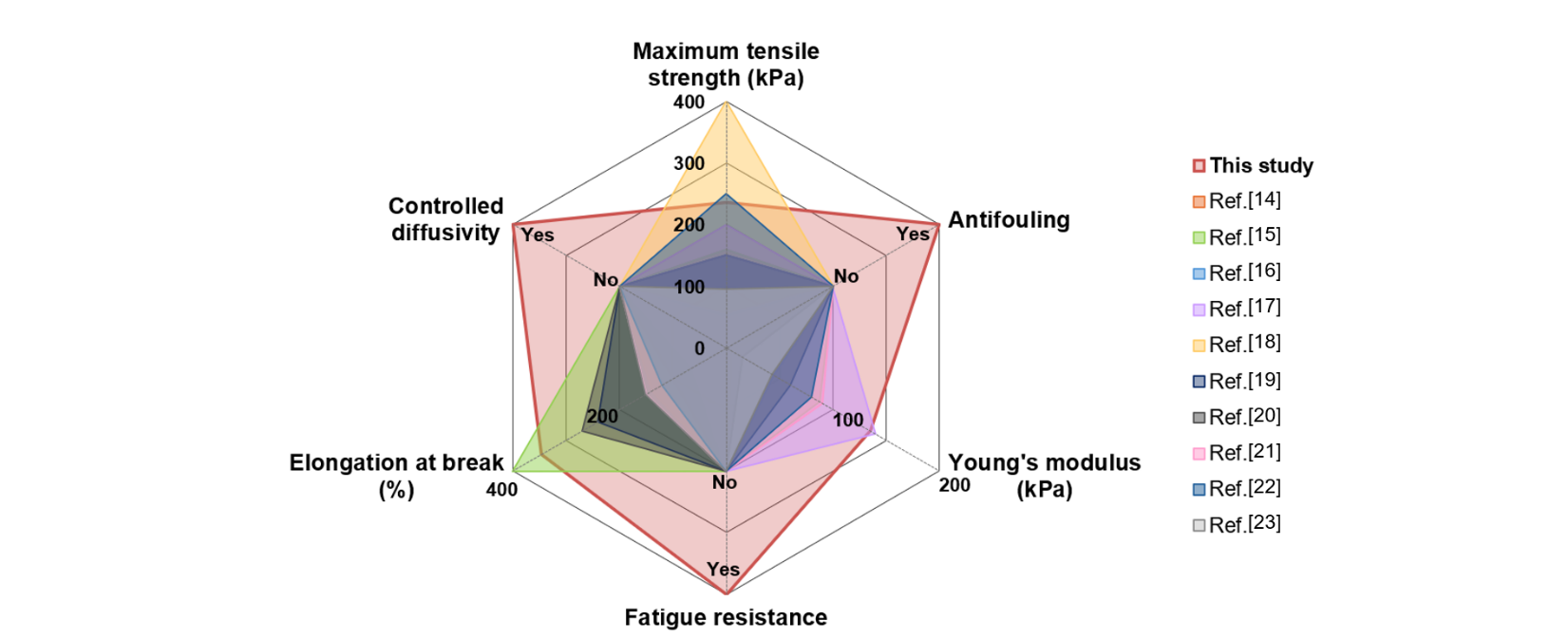


**Figure S42.** Radar map comparing the comprehensive performance of the ISC–PAHs (this study) with previously reported ion-treated or structurally modified PAHs.^[14-23]^

| **Type of hydrogel** | **COO^-^ band area (A_COO⁻_,1500–1750 cm^-1^)** | **C-O-C band area (A_C–O–C_, 950 – 1200 cm^-1^)** | **Area ratio, R (A_C-O-C_/A_COO⁻​​​​_)** |
| --- | --- | --- | --- |
| PAH | 5.25042 | 0.75039 | 0.14291 |
| CaCl_2_–PAH | 5.80816 | 0.81614 | 0.14051 |
| Na_2_SO_4_–PAH | 4.68399 | 6.07023 | 1.29595 |
| Na_2_SO_4_/CaCl_2_–PAH | 3.79871 | 3.82504 | 1.00693 |
| CaCl_2_/Na_2_SO_4_–PAH | 4.71368 | 17.53823 | 3.72070 |

**Table S1.** Summary of the reference band area (COO^-^ band area), integrated C–O–C band area, and the corresponding area ratio (R).

| **Materials** | **Maximum tensile strength (kPa)** | | **Elongation at break (%)** | | **Young’s modulus (kPa)** | | **Ref.** | |
| --- | --- | --- | --- | --- | --- | --- | --- | --- |
| Sodium alginate(SA)/ PVA/PEG-grafted graphene oxide | ≈ 105 – 130 | | ≈ 80 – 100 | | ≈ 0.5 – 10 | | ^[14]^ | |
| PVA–Alginate/FP (Fe³⁺/Protocatechualdehyde dynamic crosslinking) | ≈ 55 – 90 | | ≈ 400 – 517 | | ≈ 34 – 150 | | ^[15]^ | |
| Sodium alginate/polyvinyl alcohol blend | ≈ 35 – 52 | | ≈ 90 – 150 | | ≈ 20 – 45 | | ^[16]^ | |
| Sodium alginate/PVA/PEGDA  /Aloe vera | | ≈ 26 – 77 | ≈ 49 – 70 | | ≈ 40 – 90 | | ^[17]^ | |
| PVA/SA/E–Ag@Ti₃C₂@H(Epigallocatechin gallate–Silver nanoparticle–Ti₃C₂) | ≈ 400 – 450 | | ≈ 140 – 150 | | ≈ 120 – 130 | | ^[18]^ | |
| SA/PVA/Curcumin | ≈ 50 – 200 | | ≈ 220 – 260 | | ≈ 30 – 90 | | ^[19]^ | |
| PVA/CMC(Carboxymethyl cellulose)/SA | ≈ 110 – 330 | | ≈ 250 – 300 | | ≈ 25 – 40 | | ^[20]^ | |
| PVA/SA/Lig–Ag NPs(Lignin silver nanoparticles) | ≈ 120 – 340 | | ≈ 60 – 130 | | ≈ 120 – 240 | | ^[21]^ | |
| PVA/SA/PEG/NaBF₄ | ≈ 50 – 400 | | ≈ 98 – 140 | | ≈ 20 – 180 | | ^[22]^ | |
| Alg/PVA/Graphene oxide(GO) | ≈ 200 – 150 | | ≈ 220 – 260 | | ≈ 100 – 130 | | ^[23]^ | |
| CaCl₂–PAH  Na₂SO₄–PAH  Na₂SO₄/CaCl₂–PAH  CaCl₂/Na₂SO₄–PAH | ≈ 64 | | ≈ 177 | | 67 ± 31 | | This  study | |
|  | ≈ 237 | | ≈ 347 | | 135 ± 9 | |  |  |
|  | ≈ 222 | | ≈ 243 | | 143 ± 5 | |  |  |
|  | ≈ 144 | | ≈ 188 | | 134 ± 15 | |  |  |

**Table S2.** Summary of maximum tensile strength, elongation at break, and Young’s modulus of ion-treated or structurally modified PAHs.^[14-23]^

| **Type of hydrogel** | **G’ (Storage modulus) / Pa** | **Ɛ (Mesh size) / nm** |
| --- | --- | --- |
| PAH | 548.9 ± 58.9 | 16.5 ± 0.7 |
| CaCl_2_–PAH | 68799.7 ± 8194.9 | 4.33 ± 0.2 |
| Na_2_SO_4_–PAH | 9818.0 ± 1259.7 | 7.85 ± 0.4 |
| Na_2_SO_4_/CaCl_2_–PAH | 63071.3 ± 1526.9 | 4.46 ± 0.1 |
| CaCl_2_/Na_2_SO_4_–PAH | 505128.3 ± 46896.8 | 2.66 ± 0.1 |

**Table S3.** Storage modulus (G’) of ISC–PAH and the corresponding RET-derived mesh size (*Ɛ*). Data are presented as mean ± SD, *n* = 3.

| **Type of**  **biosensor** | **Materials/ Platforms** | **Sensitivity**  **/Accuracy** | **Limit of Detection (LOD, mM)** | **Linear Dynamic Range (mM)** | **Mechanical property** | **Ref.** |
| --- | --- | --- | --- | --- | --- | --- |
| Wearable biosensor | Au@MOG hydrogel | 13. 94 mA·mM^-1^ | 0.001 | 0.002 – 0.6 | Not reported  (indirectly mentioned that the hydrogel remained stable under bending) | ^[24]^ |
| Wearable biosensor | Agarose hydrogel/PB–PEDOT/SPCE | 49.65 mA·mM^-1^ | 0.00625 | 0.006 – 0.8 | Not reported  (indirectly discussed as a soft, conformal, and adhesive hydrogel patch maintaining structural integrity) | ^[25]^ |
| Wearable biosensor | PEDOT:PSS conductive hydrogel/PB NPs | 340.1 mA·mM^-1^ | 0.00085 | 0.001 – 3.243 | Not reported  (indirectly mentioned as a highly flexible and stretchable hydrogel) | ^[26]^ |
| Implantable  biosensor | Abbott FreeStyle Libre 3 (Commercial CGM) | (MARD ≈ 9-10 %) | ~2  (clinical lower limit) | 2 – 27 | Not reported | ^[27]^ |
| Implantable  biosensor | Dexcom G6 CGM, Dexcom | (MARD ≈ 8.2 %) | ~2  (clinical lower limit) | 2 – 22 | Not reported | ^[28]^ |
| Implantable  biosensor | Boronic acid-based hydrogel immobilized on a quartz crystal | High linearity (R² = 0.993-0.998) | 1.1 | 1.1 – 33 | Not reported (indirectly discussed as the durability and recoverability of hydrogel) | ^[29]^ |
| Wearable biosensor & Implantable  biosensor | Na₂SO₄–PAH | 1.813 mA·mM^-1^ | 0.0154 | 0.1 – 1.0 | Reported  (Tensile strength ≈ 237 kPa, Elongation ≈ 347%, Young’s modulus = 135 ± 9 kPa) | This  study |
|  | CaCl₂/Na₂SO₄–PAH | 0.07737 mA·mM^-1^ | 1.36 | 2 – 30 | Reported  (Tensile strength ≈ 144 kPa, Elongation ≈ 188%, Young’s modulus = 134 ± 15 kPa) |  |

**Table S4.** Comparative analysis of glucose biosensors: wearable sweat biosensors^[24-26]^ and implantable interstitial fluid (ISF) biosensors.^[27-29]^

**Reference**

[1] Guo J, Wang Y, Cai Y, Zhang H, Li Y, Liu D, "Ni-doping Cu-Prussian blue analogue/carbon nanotubes composite (Ni-CuPBA/CNTs) with 3D electronic channel-rich network structure for capacitive deionization", *Desalination* 2022, 528, 115622.

[2] Vokhmyanina DV, Sharapova OE, Karyakin AA, "Ultra-stable biosensor transducer for continuous monitoring", *Biosensors and Bioelectronics* 2025), 117638.

[3] Sun T, Tsaava T, Peragine J, et al., "Flexible IrOx neural electrode for mouse vagus nerve stimulation", *Acta biomaterialia* 2023, 159, 394-409.

[4] Bjånes DA, Kellis S, Nickl R, et al., "Quantifying physical degradation alongside recording and stimulation performance of 980 intracortical microelectrodes chronically implanted in three humans for 956–2246 days", *medRxiv* 2024),

[5] Yang H, Kang SK, Choi CA, et al., "An iridium oxide reference electrode for use in microfabricated biosensors and biochips", *Lab on a Chip* 2004, 4(1), 42-46.

[6] Shu W, Han C, Wang X, "Prussian blue analogues cathodes for nonaqueous potassium‐ion batteries: past, present, and future", *Advanced Functional Materials* 2024, 34(1), 2309636.

[7] Wessells CD, Peddada SV, Huggins RA, Cui Y, "Nickel hexacyanoferrate nanoparticle electrodes for aqueous sodium and potassium ion batteries", *Nano letters* 2011, 11(12), 5421-5425.

[8] La Count TD, Jajack A, Heikenfeld J, Kasting GB, "Modeling Glucose Transport From Systemic Circulation to Sweat", *Journal of Pharmaceutical Sciences* 2019, 108(1), 364-371.

[9] Cengiz E, Tamborlane WV, "A Tale of Two Compartments: Interstitial Versus Blood Glucose Monitoring", *Diabetes Technology & Therapeutics* 2009, 11(S1), S-11-S-16.

[10] Cobelli C, Schiavon M, Dalla Man C, Basu A, Basu R, "Interstitial Fluid Glucose Is Not Just a Shifted-in-Time but a Distorted Mirror of Blood Glucose: Insight from an In Silico Study", *Diabetes Technology & Therapeutics* 2016, 18(8), 505-511.

[11] Basu A, Dube S, Slama M, et al., "Time Lag of Glucose From Intravascular to Interstitial Compartment in Humans", *Diabetes* 2013, 62(12), 4083-4087.

[12] Kulcu E, Tamada JA, Reach G, Potts RO, Lesho MJ, "Physiological Differences Between Interstitial Glucose and Blood Glucose Measured in Human Subjects", *Diabetes Care* 2003, 26(8), 2405-2409.

[13] Davey RJ, Low C, Jones TW, Fournier PA, "Contribution of an Intrinsic Lag of Continuous Glucose Monitoring Systems to Differences in Measured and Actual Glucose Concentrations Changing at Variable Rates in Vitro", *Journal of Diabetes Science and Technology* 2010, 4(6), 1393-1399.

[14] Mehrjou A, Hadaeghnia M, Ehsani Namin P, Ghasemi I, "Sodium alginate/polyvinyl alcohol semi-interpenetrating hydrogels reinforced with PEG-grafted-graphene oxide", *International Journal of Biological Macromolecules* 2024, 263, 130258.

[15] Zeng H, Tang L, Huang L, et al., "A novel multi-functional PVA- alginate hydrogel with dynamic bond crosslinking for infected wound repair", *Carbohydrate Polymers* 2025, 362, 123636.

[16] Wei Q, Yang R, Sun D, et al., "Design and evaluation of sodium alginate/polyvinyl alcohol blend hydrogel for 3D bioprinting cartilage scaffold: molecular dynamics simulation and experimental method", *Journal of Materials Research and Technology* 2022, 17, 66-78.

[17] Bialik-Wąs K, Pluta K, Malina D, Barczewski M, Malarz K, Mrozek-Wilczkiewicz A, "Advanced SA/PVA-based hydrogel matrices with prolonged release of Aloe vera as promising wound dressings", *Materials Science and Engineering: C* 2021, 120, 111667.

[18] Liang Z, Sang Z, Li Y, Chen Z, Mei X, Ren X, "Enhancing the performance of injectable self-activating PVA-alginate hydrogel by Ag@MXene nanozyme as NIR responsive and photoenhanced antibacterial platform for wound healing", *Carbohydrate Polymers* 2025, 357, 123434.

[19] Hsiao Y-C, Lee L-C, Lin Y-T, et al., "Stretchable polyvinyl alcohol and sodium alginate double network ionic hydrogels for low-grade heat harvesting with ultrahigh thermopower", *Materials Today Energy* 2023, 37, 101383.

[20] Chen K, Zong T, Chen Q, Liu S, Xu L, Zhang D, "Preparation and characterization of polyvinyl alcohol/ sodium alginate/carboxymethyl cellulose composite hydrogels with oriented structure", *Soft Materials* 2022, 20(1), 99-108.

[21] Yu J, Ran F, Li C, et al., "A Lignin Silver Nanoparticles/Polyvinyl Alcohol/Sodium Alginate Hybrid Hydrogel with Potent Mechanical Properties and Antibacterial Activity", *Gels* 2024, 10(4), 240.

[22] Chen Z, Fu X, Chen B, et al., "Tough, Freeze-Resistant, Pressure-Response Gel Polymer Electrolytes with Redox Pairs for Flexible Supercapacitors", *ACS Applied Materials & Interfaces* 2024, 16(19), 24840-24850.

[23] Abouzeid RE, Salama A, El-Fakharany EM, Guarino V, "Mineralized Polyvinyl Alcohol/Sodium Alginate Hydrogels Incorporating Cellulose Nanofibrils for Bone and Wound Healing", *Molecules* 2022, 27(3), 697.

[24] Zhou D, Zhang S, Khan AU, Chen L, Ge G, "A wearable AuNP enhanced metal–organic gel (Au@MOG) sensor for sweat glucose detection with ultrahigh sensitivity", *Nanoscale* 2024, 16(1), 163-170.

[25] Lin P-H, Sheu S-C, Chen C-W, Huang S-C, Li B-R, "Wearable hydrogel patch with noninvasive, electrochemical glucose sensor for natural sweat detection", *Talanta* 2022, 241, 123187.

[26] Xu C, Jiang D, Ge Y, et al., "A PEDOT:PSS conductive hydrogel incorporated with Prussian blue nanoparticles for wearable and noninvasive monitoring of glucose", *Chemical Engineering Journal* 2022, 431, 134109.

[27] Abbott. FreeStyle Libre Continuous Glucose Monitoring System. Updated accessed: August 2025. <https://www.freestylelibre.us/>

[28] Dexcom Inc. . Dexcom Continuous Glucose Monitoring System. Updated accessed: August 2025. <https://www.dexcom.com/>

[29] Dou Q, Hu D, Gao H, et al., "High performance boronic acid-containing hydrogel for biocompatible continuous glucose monitoring", *RSC Advances* 2017, 7(66), 41384-41390.
